# SIRT6 mono-ADP ribosylates KDM2A to locally increase H3K36me2 at DNA damage sites to inhibit transcription and promote repair

**DOI:** 10.18632/aging.103567

**Published:** 2020-06-25

**Authors:** Sarallah Rezazadeh, David Yang, Seyed Ali Biashad, Denis Firsanov, Masaki Takasugi, Michael Gilbert, Gregory Tombline, Natarajan V. Bhanu, Benjamin A. Garcia, Andrei Seluanov, Vera Gorbunova

**Affiliations:** 1 Department of Biology, University of Rochester, Rochester, NY 14627, USA; 2 Department of Biochemistry and Biophysics, Perelman School of Medicine, University of Pennsylvania, Philadelphia, PA 19104, USA

**Keywords:** SIRT6, DNA repair, transcription, genome stability, longevity

## Abstract

When transcribed DNA is damaged, the transcription and DNA repair machineries must interact to ensure successful DNA repair. The mechanisms of this interaction in the context of chromatin are still being elucidated. Here we show that the SIRT6 protein enhances non-homologous end joining (NHEJ) DNA repair by transiently repressing transcription. Specifically, SIRT6 mono-ADP ribosylates the lysine demethylase JHDM1A/KDM2A leading to rapid displacement of KDM2A from chromatin, resulting in increased H3K36me2 levels. Furthermore, we found that through HP1α binding, H3K36me2 promotes subsequent H3K9 tri-methylation. This results in transient suppression of transcription initiation by RNA polymerase II and recruitment of NHEJ factors to DNA double-stranded breaks (DSBs). These data reveal a mechanism where SIRT6 mediates a crosstalk between transcription and DNA repair machineries to promote DNA repair. SIRT6 functions in multiple pathways related to aging, and its novel function coordinating DNA repair and transcription is yet another way by which SIRT6 promotes genome stability and longevity.

## INTRODUCTION

One of the most deleterious forms of DNA damage, DNA double-strand breaks (DSBs) can trigger cell death if left unrepaired. Faulty DSB repair may lead to cancer [1] and contribute to premature aging. DSBs are repaired by two main pathways: non-homologous end-joining (NHEJ), which operates throughout the cell cycle, and homologous recombination (HR) which is limited to S and G2 cell-cycle phases [2]. In addition, if the break occurs in a transcribed euchromatic region, local transcription is transiently silenced in *cis* to allow repair [3–7]. While much progress has been made in studying DSB repair mechanisms, a clear model that includes influences of chromosomal context and transient transcriptional silencing remains incomplete.

Protein posttranslational modifications (PTMs) such as phosphorylation, acetylation, methylation, and ubiquitination play an important role in modulating repair-pathway-choice and efficiency of DSB repair. Histones, the basic protein units of chromatin, are subjected to modifications such as acetylation, phosphorylation and ubiquitylation that alter the properties of chromatin and influence DNA repair. For instance, activation of the apical DDR protein kinases ATM (ataxia telangiectasia mutated), ATR (ATM and Rad3 related) and DNA-PK leads to phosphorylation of the histone variant H2AX on chromatin flanking DSB sites [8]. This phospho-form of H2AX, termed γH2AX, is one of the earliest chromatin markers of DSBs and is critical for the accumulation of repair and signaling proteins, such as 53BP1, into foci at DNA-damage sites. Similarly, ubiquitin E3 ligases RNF8 and RNF168 are recruited to DSB sites and mediate histone H2A and H2AX ubiquitylation that is vital for effective repair [9]. Histone acetylation and methylation, together with the enzymes mediating their addition and removal, have also been implicated in the DDR. While the role of histone acetylation and methylation in transcriptional regulation are well established, their modes of action in DNA repair are less clear [5].

The enrichment of H3K36me at sites of DSBs in mammalian cells suggest a potential role of H3K36me in DNA damage sensing and*/*or repair. Different forms of H3K36me (di- or tri-) may serve as codes for cells to activate different repair mechanisms. For example, in mammalian cells, H3K36me3 facilitates homologous recombination repair [10]. H3K36me3 also precisely localizes the DNA mismatch recognition hMSH3 and hMSH6 proteins onto chromatin [11]. In fission yeast, new results suggest that H3K36me3 is the major form of methylation on H3K36 to mediate tolerance to MMS [12]. Histone H3K36 dimethylation appears to influence NHEJ [13, 14]. During NHEJ, ionizing radiation induces H3K36me2 by the DNA repair protein Metnase (also known as SETMAR), a SET histone methyl transferase domain-containing protein, at DSB sites. H3K36me2 near DSBs can be enhanced through local generation of Fumarate and be counteracted by expression of the KDM2 histone demethylases. Once accumulated, H3K36me2 improves the association of early DNA repair components, including Ku70, with DSBs [14, 15]. Though, the new data highlights the role of H3K36 di-methyl in enhancement of NHEJ repair, the exact mechanism is not known.

Recent reports show that mRNA transcription by RNA polymerase II is repressed in *cis* to DNA DSBs in active genes in an ATM and DNA-PK catalytic subunit-dependent manner [16]. Furthermore, consistent with the idea of transcriptional arrest [5, 17], exclusion of the RNA processing factor THRAP3 (thyroid hormone receptor associated protein 3) from the vicinity of DSBs has also been shown, and this is dependent on the E3-ubiquitin ligases RNF8 and RNF168 [18]. It is known that failure to silence transcription in the immediate proximity of DSBs has a negative impact on DNA repair efficiency [19].

Class III HDACs, also known as Sirtuins, are NAD-dependent enzymes and consist of SIRT1-7 that are closely related to the yeast Sir2 protein [20]. SIRT6 is a histone deacetylase, deacylase and mono-ADP ribosyl transferase involved in diverse processes including metabolism, transcription and the DNA repair [21]. SIRT6 is a longevity gene that extends lifespan when overexpressed [22]. SIRT6 mediates genome stability by enhancing DNA repair [23–26] and silencing repetitive elements [27, 28]. SIRT6 also plays important role in promoting stem cell homeostasis [29, 30].

We previously demonstrated that SIRT6 promotes NHEJ repair by mono-ADP-ribosylating PARP1 [23]. Here we aimed to identify other SIRT6 mono-ADP-ribosylation substrates relevant to DNA repair. Mass spectrometry followed by mutagenesis showed that KDM2A histone demethylase is mono-ADP-ribosylated by SIRT6. KDM2A/B lysine de-methylases work on H3K36me2 to produce mono methyl form of H3K36 [14]. Interestingly, recent data showed that ATM-mediated phosphorylation removes KDM2A from chromatin in response to DNA damage [31]. Here we demonstrate that SIRT6-mediated mono-ADP ribosylation displaces KDM2A from broken chromatin leading to accumulation of H3K36me2 mark. This in turn, promotes H3K9me3 near the break site, leading to inhibition of transcription initiation and improved NHEJ efficiency. Our results suggest that SIRT6 activity is required to prevent transcription through DSBs, pointing to a novel role for SIRT6 in integrating transcription and DNA repair.

## RESULTS

### KDM2A is mono-ADP ribosylated by SIRT6

**Figure 1:**
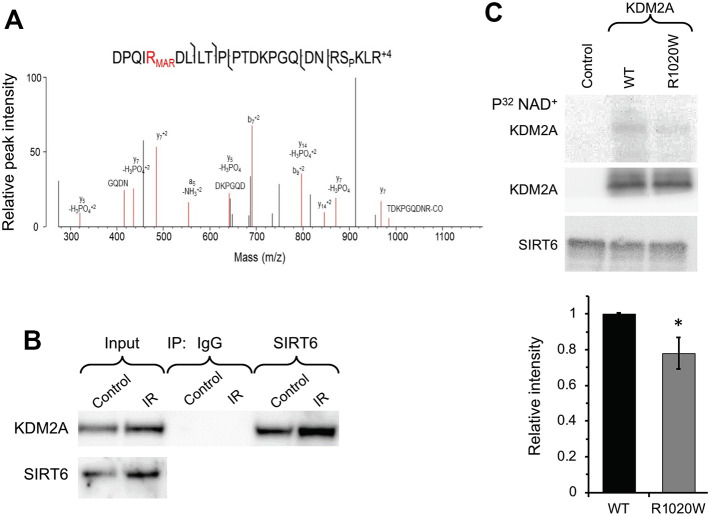
**SIRT6 mono-ADP ribosylates KDM2A on R1019/R1020 (mouse/human).** (**A**) Mass spec analysis of mouse KDM2A mono-ADP ribosylation. MS2 fragment spectrum of a KDM2A peptide obtained from mouse embryonic fibroblast cells supporting R1019 modified by a ribose-phosphate group indicating mono-ADP ribosylation (MAR). Pictured is an assignment showing ribose-phosphate modified R1019. A full compiled report downloaded from Protein Prospector of the MS2 data for this peptide can be found in the Supplementary Data (Supplementary Data 1). (**B**) SIRT6 interacts with KDM2A. coimmunoprecipitation of KDM2A was observed with antibodies directed against SIRT6 before and after irradiation (IR) (the experiment was repeated three times). (**C**) SIRT6 mono-ADP ribosylates KDM2A *in vitro*. KDM2A R1020W mutation significantly decreases SIRT6 mono-ADP ribosylation signal. Top panel. SIRT6 protein was incubated with purified human wild type flag-KDM2A or mutant R1020W proteins in the presence of radiolabeled NAD+. Mono-ADP ribosylation was detected by transfer of the radiolabel to the substrate. Lower panel. Quantification of upper gel. Graph represents mean ± SD of three experiments. *p > 0.05.

SIRT6 is a chromatin regulator which plays critical roles in both gene silencing (20) and DNA repair through PARP1 activation (21). Here we aimed to identify SIRT6 mono-ADP ribosylation substrates involved in DNA repair and transcription using mass spectrometry. Total proteins were extracted from the wild type and SIRT6 knock out mouse embryonic fibroblasts treated with paraquat, and then enriched for mono-ADP-ribosylated peptides using titanium oxide [32]. The eluted samples were subjected to high-resolution bottom-up mass spectroscopy. We found that R1019 (corresponding to R1020 in human) on histone de-methylase KDM2A is mono ADP-ribosylated only in the wild type but not in the SIRT6 knockout cells (Figure 1A). Interestingly, the most frequent mutation in KDM2A found in human cancer is R1020W, which occurs at the frequency 0.6% (http://www.cbioportal.org).

To validate our observation that SIRT6 mono-ADP ribosylates of KDM2A we sought to determine whether SIRT6 and KDM2A interact. Co-immunoprecipitation (Co-IP) indicated that SIRT6 interacts with KDM2A under basal conditions as well as post irradiation (Figure 1B). Consistent with mass spec results, in vitro ribosylation assay using recombinant SIRT6 and wild type or non-ribosylatable human R1020W mutant KDM2A proteins in the presence of P^32^ labeled-NAD^+^ showed ribosylation on wild type KDM2A, and to a lesser degree on KDM2A-R1020W mutant (Figure 1C). Taken together these results suggest that KDM2A is a mono-ADP ribosylation substrate of SIRT6.

### SIRT6-mediated ribosylation of KDM2A displaces KDM2A from chromatin and promotes DNA repair

**Figure 2:**
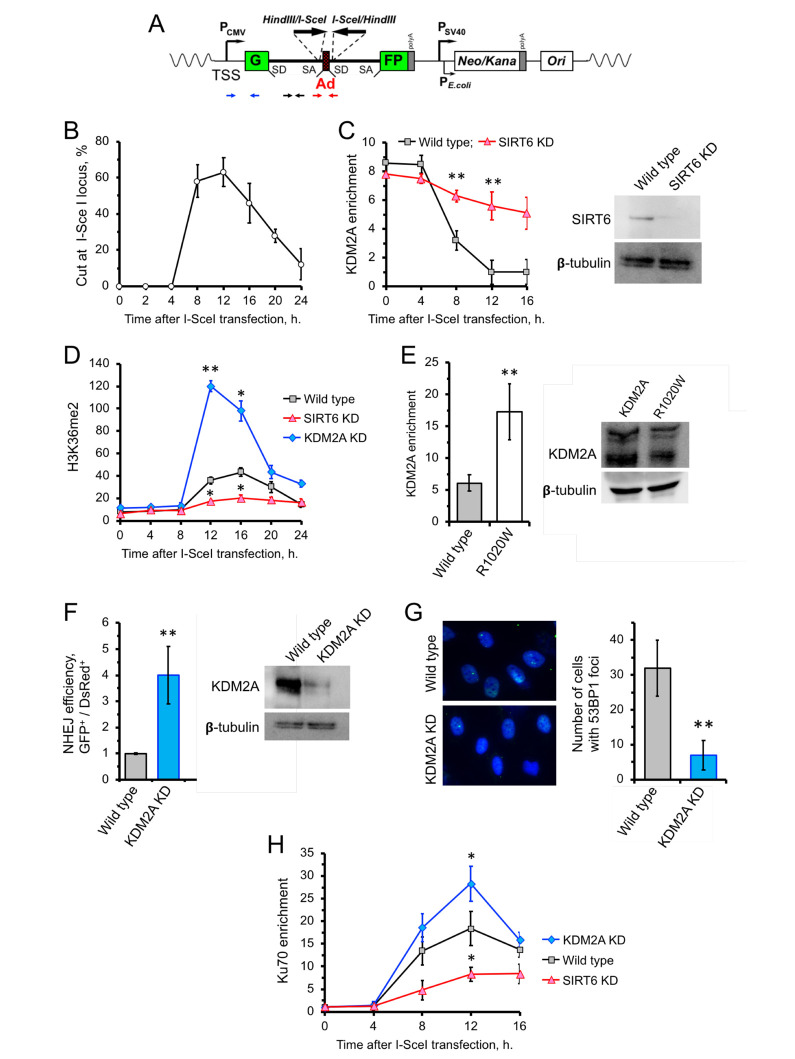
**SIRT6 mediates KDM2A displacement from chromatin to enhance NHEJ.** (**A**) Schematic representation of the GFP-based NHEJ reporter, which represents an RNA Pol II-transcribed gene. DSBs are introduced by transient expression of I-SceI enzyme. The positions of the primer used in this study are indicated by small arrows below: primers for quantification of the preRNA (small black arrows); quantification of DNA DSBs (small red arrows), chromatin IP (blue arrows). (**B**) Quantification of the cutting efficiency at different time points after transfection with I-SceI vector. DSBs were quantified as a ratio between the product obtained with primers amplifying across the break (red arrows) to a PCR product from a gene away from DSB site (Supplementary Figure 2). The experiment was repeated three times. (**C**) KDM2A dissociation from broken chromatin is SIRT6-dependent. Time-course chromatin-IP was performed using antibody against endogenous KDM2A in human skin fibroblasts harboring chromosomally-integrated NHEJ GFP reporter with I-SceI sites. Western blot shows depletion of SIRT6. (**D**) H3K36me2 accumulation post DSB induction is SIRT6-dependent. ChIP analysis of H3K36me2, at different time points post transfection with I-SceI vector using antibody specific for H3K36me2. qPCR was performed using primers positioned 50 bp downstream of TSS. (**E**) Non-ribosylatable KDM2A R1020W mutant bids to DSBs more efficiently than the wild type protein. Human skin fibroblasts carrying I-SceI reporter cassette were transfected with wild type or mutant (R1020W) KDM2A and I-SceI encoding vectors. At 12 hours post transfection cells were harvested followed by ChIP-qPCR with antibodies against FLAG. Western blot showing the levels of wild type and mutant FLAG-KDM2A proteins in cells after transfection. (**F**) Cells depleted of KDM2A have enhanced NHEJ repair efficiency. Cells were treated with siRNA to KDM2A four days before transfection with I-SceI. NHEJ efficiency was measured by reactivation of the GFP reporter normalized to transfection efficiency (DsRed) 48 h after I-SceI transfection. Western blot shows KDM2A depletion. (**G**) Downregulation of KDM2A reduces the number of 53BP1 foci. KDM2A knocked down cells were grown to confluency and fixed for IF experiment using antibody against 53BP1. On the right; quantification of the IF image obtained from 100 nuclei. All experiments were repeated at least three times. (**H**) Ku70 recruitment to DSB site requires SIRT6, and is counteracted by KDM2A. Time course of ChIP experiment using antibody against Ku70. The experiment was repeated three times. *p < 0.05; **p <0.01.

Besides its role in transcription, H3K36me2 also been reported to be involved in DNA damage response. Particularly, upon double- stranded DNA break, H3K36me2 levels drastically increase proximal to the break site [15, 31]. This raises the possibility that SIRT6 inhibits KDM2A to enhance enrichment of H3K36me2. Consistently, we observed enhanced level of H3K36me2 in cells overexpressing SIRT6 (Supplementary Figure 1A). This effect could be mediated either through inhibition of KDM2A enzymatic activity or KDM2A displacement from chromatin. To test this, we took advantage of the hTERT-immortalized human fibroblast NHEJ reporter cell line carrying a construct containing a GFP with a recognition site for the I-SceI endonuclease (Figure 2A). Transfection of the NHEJ reporter cells with I-SceI resulted in cleavage of the I-SceI site, as demonstrated by qPCR (Figure 2B). We then used ChIP to study the kinetics of KDM2A near DNA-break sites. Induction of DSBs by I-*Sce*I in GFP-reporter cells resulted in a concomitant dissociation of KDM2A from chromatin near the DNA break, with the lowest levels of KDM2A on chromatin occurring at 12 h post I-SceI transfection (Figure 2C), which is the time of maximum I-SceI cutting (Figure 2B). Such a pattern, however, was attenuated in cells depleted of SIRT6 by shRNA (SIRT6 KD) (Figure 2C). This suggests that SIRT6 is required for displacement of KDM2A from broken euchromatin upon DNA damage. The departure of KDM2A from chromatin upon DSB induction was associated with robust increase in H3K36me2 level 12 h post I-SceI transfection (Figure 2D). In the SIRT6 KD cells, however, there was no significant increase in H3K36me2 at all time points tested (Figure 2D). The strongest increase in H3K36me2 mark was observed in KDM2A shRNA-depleted cells (Figure 2D). We then examined whether mono-ADP ribosylation is required for KDM2 displacement. KDM2A R1020W mutant showed 3-fold higher enrichment on chromatin near I-SceI cut site at 12 h following I-SceI transfection (Figure 2E).

We next tested the effect of KDM2A knockdown on the efficiency of DNA repair by NHEJ. NHEJ reporter cells contain GFP-based cassette where I-SceI sites are flanking a “killer” exon inserted in the GFP gene (Figure 2A). In the presence of the killer exon GFP gene is inactive, while I-SceI digestion followed by removal of the exon and NHEJ event reactivates the GFP. NHEJ reporter cells were transfected with I-SceI and DsRed (to normalize for transfection efficiency). Repair efficiency was calculated as a ratio of GFP+/dsRed+ cells. We observed enhanced NHEJ repair efficiency in cells with KDM2A knockdown (Figure 2F). This suggests that KDM2A has negative impact on DNA NHEJ repair in transcribed chromatin. Furthermore, KDM2A knockdown cells has significantly fewer spontaneous 53BP1 foci which serve as a marker of DSBs (Figure 2G). Collectively, these results indicate that KDM2A negatively regulates NHEJ, and downregulation or displacement of KDM2A from chromatin promotes NHEJ in transcribed chromatin.

To test whether mono-ADP ribosylation of KDM2A affects its demethylase activity, we pre-incubated the KDM2A protein with SIRT6 and NAD^+^. There was no detectable difference in de-methylation activity of KDM2A in the presence or absence of SIRT6 and NAD^+^ (Supplementary Figure 1B). This result suggests that mono-ADP ribosylaion acts to remove KDM2A from chromatin but does not inhibit its enzymatic activity.

### H3K36me2 enhances DSB repair factors recruitment to the broken euchromatin

Previous studies showed that there is a direct correlation between H3K36me2 and Ku70 enrichment post DSB induction (14,15). We then examined the status of Ku70 recruitment near the *I-SceI* cut site post DSB induction in cells depleted of KDM2A using a different set of primers which specifically target within 300 bp of the DSB. We found that downregulation of KDM2A results in enhancement of H3K36me2 deposition and Ku70 recruitment (Figure 2H). To further test if the Ku70 recruitment also depends on SIRT6, we depleted the cells of SIRT6 protein by siRNA. After induction of DSB through *I-SceI* transfection we detected significantly less Ku70 on the DSB site in SIRT6 KD cells comparing to control cells (Figure 2H). Together these results suggest that SIRT6 enhances Ku70 recruitment to the broken chromatin and inhibition of KDM2A facilitates this event possibly by increasing the H3K36me2 level.

### H3K36me2 suppresses transcription initiation on broken chromatin

KDM2A is enriched on CpG islands, which ensures maintenance of the promoter in an open, transcriptionally permissive mode [33–37]. Since we observed the enrichment of H3K36me2 on the TSS after DSB induction concomitant with KDM2A displacement, we hypothesized that H3K36me2 is responsible for the suppression of transcription initiation of damaged genes. To investigate the dynamics of RNA polymerase II (RNA Pol II) machinery in the presence of DSBs we preformed ChIP using antibody that specifically recognizes initiating RNA Pol II and RT-qPCR.

**Figure 3:**
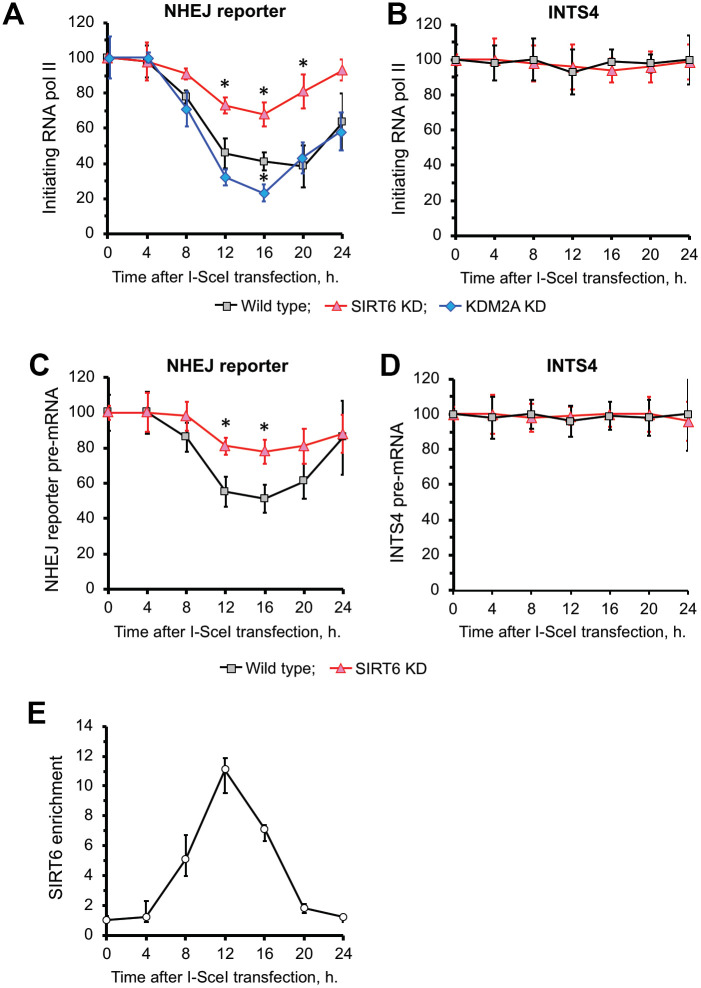
**SIRT6 is required for inhibition of transcription upon induction of DNA DSB.** (**A**) SIRT6 is required for RNA Pol II displacement upon DSB. Time course ChIP analysis of initiating (S5 phosphorylated) RNA Pol II levels on the TSS region of the GFP in NHEJ reporter upon DSB induction. (**B**) Time course ChIP analysis of initiating RNA Pol II levels on the TSS region of INTS4 gene, which does not undergo a DSB (negative control to **A**). (**C**) qRT-PCR analysis of GFP premature RNA expression at different time points after transfection with I-SceI vector. (**D**) qRT-PCR analysis of INTS4 premature RNA expression (control for **C**). (**E**) SIRT6 recruitment to DSB peaks within 12 h post DSB induction. Chromatin IP of endogenous SIRT6 in skin fibroblasts. Chromatin samples were analyzed by qPCR using primers flanking I-SceI cut sites with antibodies against SIRT6 or IgG. The positions of primers in NHEJ reporter and INTS4 gene are shown in Figures 2A and Supplementary Figure 2. The experiments were repeated three times. *p < 0.05.

We observed a reduction of RNA Pol II (Figure 3A) as well as nascent RNA levels of *GFP* transcribed from NHEJ reporter (Figure 3C) upon DSB induction. There was no change in RNA Pol II levels or expression on a control gene *INTS4* (see Supplementary Figure 2 for location of primers) which is an RNA Pol II-transcribed gene not subjected to DSB (Figure 3B, D). Reduced RNA Pol II levels at DSB site were associated with recruitment of SIRT6 (Figure 3E). Upon completion of repair at 24 h (Figure 2B) we observed restoration of transcription (Figure 3A) associated with SIRT6 departure from chromatin (Figure 3E) and reduction in H3K36me2 levels (Figure 2D). Cumulatively these results suggest that SIRT6 promotes increase in H3K36me2 levels and inhibition of transcription upon DSB induction.

### Transient chromatin condensation follows the transcriptional suppression

**Figure 4:**
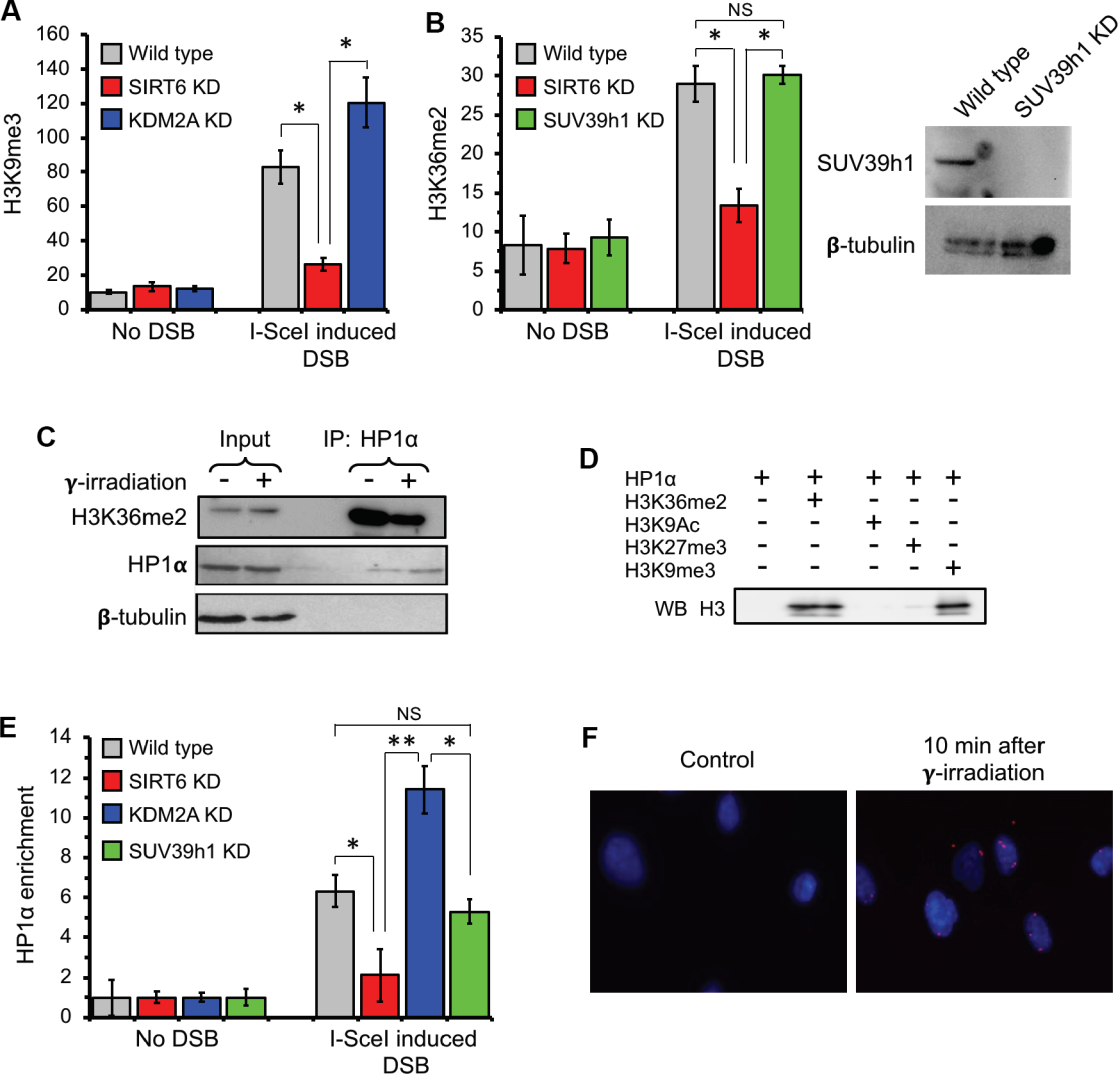
**H3K36me2 recruits HP1α and promotes deposition of H3K9me3 at DSB locus in SIRT6-dependent manner.** (**A**) SIRT6, but not KDM2A, is required for deposition of H3K9me3 at DSB locus. ChIP assay was performed 12 h post transfection with I-SceI vector using antibody against H3K9me3. (**B**) SIRT6, but not SUV39h1, is required for accumulation of H3K36me2 at DSB locus. Western blot shows depletion of SUV39h1. (**C**) HP1α binds H3K36me2 *in vivo*. Human skin fibroblast cells were irradiated with 3 Gy of γ-irradiation. Cell lysate was immunoprecipitated within 10 minutes after irradiation with antibody against HP1α and blotted against H3K36me2 (Top panel), HP1α (middle) and β-tubulin (lower panel). (**D**) HP1α binds H3K36me2 *in vitro*. Mono nucleosomes carrying either H3K36me2, H3K27me3, H3K9me3 or H3K9Ac groups were incubated with HP1α and immunoprecipitated with antibody against HP1α and blotted with H3 antibodies. (**E**) SIRT6, but not SUV39h1, is required for HP1α recruitment to DSB locus. (**F**) HP1α interacts with 53BP1 after γ-irradiation. Proximity ligation assay (PLA) was performed using antibodies against HP1α and 53BP1 and the PLA signal was detected within 10 minutes after irradiation. NS; not significant. All experiments were repeated three times. *p < 0.05; **p <0.01.

Formation of transcriptionally silenced micro environment is ensured by deposition of heterochromatin marks near the DSB region [5, 7, 17, 38–40]. To test if the absence of SIRT6 leads to failure in formation of the condensed chromatin we performed ChIP experiments post I-SceI transfection. We observed a substantial increase in H3K9me3 levels post DSB induction close to the transcription start site (Figure 4A). Importantly, this was strongly attenuated in SIRT6 knockdown cells.

To test whether SIRT6 is required for H3K9me3 deposition and suppression of initiating RNA Pol II proximal to the broken ends, ChIP-qPCR was performed upon DSB induction in wild type cells and cells depleted of either SIRT6 or SUV39h1. DSB resulted in reduced RNA Pol II abundancy near the DSB site (Supplementary Figure 3A) which coincided with higher level of H3K9me3 deposition (Figure 4A). The effect on RNA Pol II abundancy was abrogated by SIRT6 and SUV39h1 KD (Supplementary Figure 3A). This data provides a potential link between SIRT6-mediated displacement of KDM2A, local increase in H3K36me2, and deposition of H3K9me3 deposition near the DSB sites.

### HP1α is the reader for H3K36me2 and facilitates H3K9 tri-methylation

As H3K36me2 is increased on euchromatin upon DSB concomitantly with an increase in H3K9me3 and both are SIRT6 dependent, we hypothesized that H3K36me2 initiates the transient formation of silent chromatin near DSB site. We then set out to find the reader which recognizes H3K36me2 mark and facilitate spreading of the heterochromatin.

Heterochromatin protein 1α (HP1α) is a known reader for H3K9me3. Binding of this protein is sufficient to enhance a positive feedback loop for further spreading of the heterochromatin mark and establishment of the condensed chromatin through recruitment of SUV39h1 methyl transferase [41–44]. Co-IP showed that HP1α interacts with H3K36me2 in vivo (Figure 4C). To test whether HP1α and H3K36me2 interaction is direct, we performed *in vitro* IP using mono-nucleosomes carrying di-methyl groups on the lysine 36 and HP1α recombinant proteins. We used H3K9me3 as a positive control and H3K9Ac and H3K27me3 as negative controls. We observed HP1α protein biding to H3K36me2, which was as strong as the binding to H3K9me3. We did observe binding of HP1 α to H3K27me3 or H3K9Ac, suggesting the specificity of the HP1α towards the H3K36me2 and H3K9me3 histone marks (Figure 4D).

The association of HP1α to H3K36me2 predicts recruitment of this protein to the broken transcribing chromatin. HP1α was detectable at a significant level post DSB induction at the TSS in the wild type but not SIRT6 knock down cells (Figure 4E). Interestingly, shRNA-mediated depletion of KDM2A, which leads to enhancement of H3K36me2, resulted in enhancement of the HP1α binding (Figure 4E). This data suggests that HP1α is recruited to the broken chromatin, and this is facilitated by H3K36me2. Remarkably, downregulation of H3K9me3 deposition through knock down of SUV39h1 did not influence recruitment of HP1α (Figure 4E). This result provides support for HP1α being a H3K36me2 reader and being recruited to the chromatin independent of H3K9me3 status. It also suggests that H3K36me2 nucleosome binding by HP1α facilitates further deposition of the H3K9me3.

HP1α V26M mutation has been shown to completely abrogate HP1α capacity to bind to H3K9me2/3 and subsequently disrupts its gene silencing function [45]. When we co-transfected the NHEJ reporter cell lines with either HP1α wild type or HP1α V26M mutant and I-SceI, consistent with our observation of H3K9me3 deposition following DSB, we observed that HP1α mutation abolishes the spreading of H3K9me3 mark on the DSB site by 30-fold (Supplementary Figure 3B) suggesting that HP1α is required for spreading the H3K9me3 mark around DSB sites.

To visualize HP1α binding to the DNA break sites we took advantage of a proximity ligation assay (PLA). PLA allows *in situ* detection of proteins localized in close proximity (<40 nm). Through PLA, we observed HP1α interaction with 53BP1, localized in nuclear regions of the cell (Figure 4F) 10 minutes after irradiation. This result indicates that HP1α recruitment is an early event in DNA damage response.

## DISCUSSION

Here, we describe a mechanism where SIRT6 facilitates DSB repair of transcribed chromatin by transiently silencing transcription upon DNA DSB repair. We show that SIRT6 mono-ADP ribosylates KDM2A on LRR domain at R1020 and this is required for dissociation of the enzyme from chromatin after DNA damage without affecting the de-methylation activity of KDM2A. Leucine rich repeat (LRR), one of the main domains of KDM2A, has been suggested to be important for inter-molecular interaction with the transcriptional factors [46, 47].

**Figure 5:**
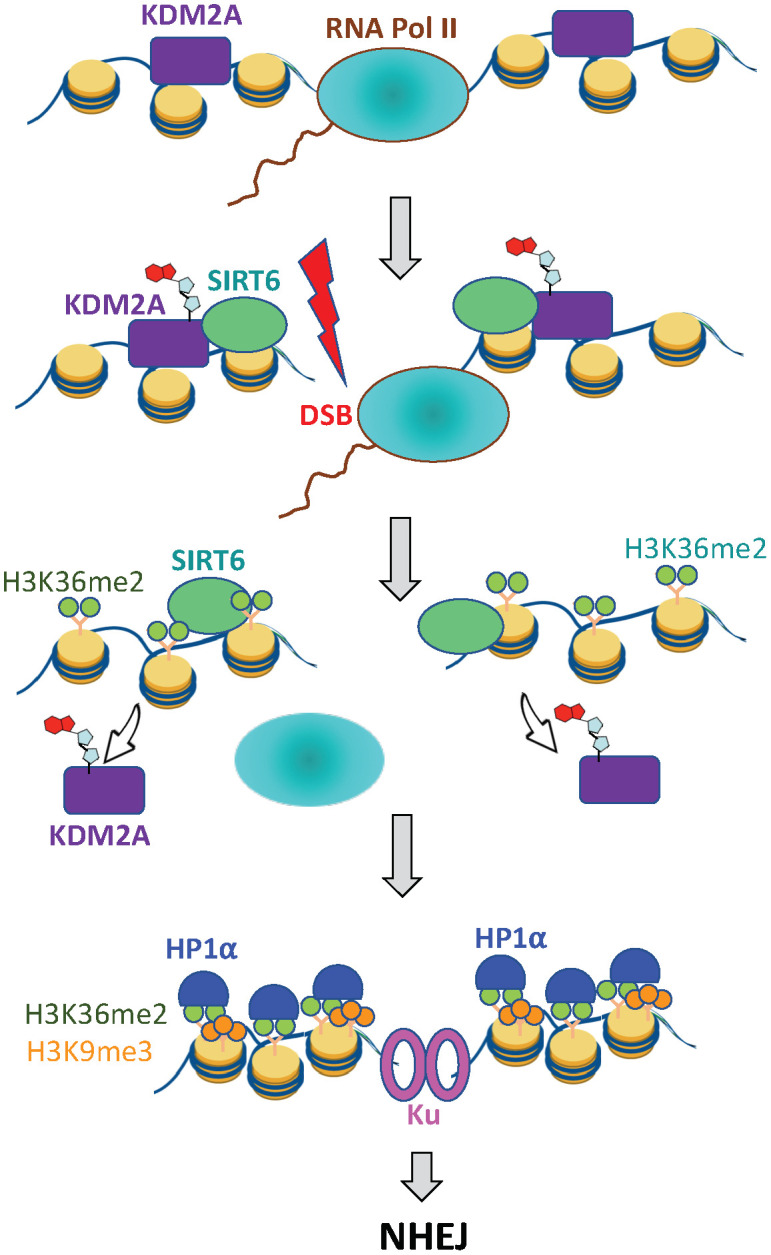
**SIRT6 prevents collision between transcription and DSB repair machineries.** Under basal conditions KDM2A demethylates H3K36 at transcription start site. Upon DNA damage SIRT6, is recruited to the DSB locus and mono-ADP ribosylates KDM2A leading to its displacement from chromatin. This leads to accumulation of H3K36me2 marks around the DSB site, which recruits HP1α and promotes deposition of H3K9me3 mark leading to local chromatin compaction. As a result, transcription is paused and DNA repair by NHEJ ensues.

We propose a model where displacement of the KDM2A at DSBs increases H3K36me2 methylation on TSS near the DSB. This promotes recruitment of additional methyl transferases, as well as HP1α, which then methylate H3K9 on nucleosomes upstream from the DSB. This process leads to cycles of H3K36 di- and H3K9 tri-methylation, which catalyzes the transient spreading of heterochromatin. This model is similar to the original mechanism of heterochromatin spreading, in which an initial nucleation event positions HP1α complexes containing H3K9 methyltransferases on the euchromatin and subsequent cycles of H3K9 methylation and loading of HP1α complexes (including SUV39h1) result in the spreading and stabilization of heterochromatin [43, 44, 48]. In this way, an initial nucleation event at DSBs can spread H3K36me2, this results in H3K9me3 deposition along the chromatin domains flanking the DSB, leading to the rapid formation of repressive chromatin at the DSB locus (Figure 5).

SIRT6 plays a central role in DSB repair acting as both early sensor of DSBs [26] and by orchestrating the recruitment of downstream repair factors and chromatin remodelers [23, 25, 49]. Both deacetylase and ADP-ribosylase activities of SIRT6 facilitate DNA repair [21]. Previously, we reported that SIRT6 mono-ADP ribosylation enhances PARP1 activity and promotes DSB repair [23]. Here we identified an additional mechanism by which SIRT6 facilitates DSB repair by mono-ADP ribosylating KDM2A. The two SIRT6 enzymatic activities may act in a concerted fashion and provide a local environment that is conducive to the DNA repair machinery. That is, SIRT6 deacetylation of H3K9ac and/or H3K56ac may occur as SIRT6 is also locally mono-ADP ribosylating PARP1 and KDM2A.

SIRT6 has recently been implicated in restraining of transcription under basal conditions in the absence of DNA damage [50]. There SIRT6 binds Pol II and prevents the release of the negative elongation factor (NELF), thus stabilizing Pol II promoter-proximal pausing [50].

Transcriptional silencing through methylation of H3K36 also been reported in *Saccharomyces cerevisiae* [51]. SET2/H3K36me3 is enriched at DSBs, and loss of SET2, the only methyltransferase for H3K36 in yeast, results in altered chromatin architecture and inappropriate resection during G1 near break sites. Additionally, null mutation in SET2 leads to spurious transcription and shortening of lifespan in yeast [52]. Deletion of the H3K36 di/tri demethylase Rph1, on the other hand, extended the yeast lifespan which was attributed to the ability of the demethylase mutant to suppress spurious cryptic transcripts [52]. Consistent with observations in yeast, loss of the worm SET2 homolog, MET-1, shortens lifespan, whereas loss of the demethylase JMJD-2 has pro-longevity effects suggesting a conserved regulatory pathway [52].

Our results are consistent with H3K36me2 serving a protective role in preserving transcriptional fidelity. Accumulation of H3K36me2 at DSB sites leads to local transcriptional silencing which prevents the conflict between transcription and DNA repair. This early event is followed by enhanced recruitment of Ku complex to the processed chromatin and promotes DNA repair.

In summary, we demonstrate that SIRT6 promotes DNA repair of transcribed genes by preventing a collision between transcription and DNA repair by a novel mechanism involving mono-ADP ribosylation of KDM2A. This may represent a conserved mechanism of longevity as longevity associated genes are enriched for processes related to DNA repair [53]. Activating SIRT6 has been shown to extend lifespan [22], and coordination between transcription and DNA repair may be yet another SIRT6 function beneficial for genome maintenance and lifespan.

## MATERIALS AND METHODS

### Cell lines and treatments

We obtained mouse embryonic fibroblasts (MEFs) from SIRT6 wild type and knockout embryos and immortalized them by using a standard 3T3 protocol. MEFs were grown in Dulbecco’s Modified Eagle’s Medium (DMEM) supplemented with sodium pyruvate, nonessential amino acids, antibiotics (penicillin and streptomycin) and 15% fetal bovine serum. The media was replaced with Minimum Essential Eagle’s Medium (MEM) supplemented with nonessential amino acids, antibiotics (penicillin and streptomycin), and 15% fetal bovine serum before adding the hydrogen peroxide. Oxidative stress was induced by noncytotoxic levels of hydrogen peroxide or paraquat. Cell treatments included paraquat (500 μM), gamma irradiation (3 Gy). All compounds were purchased from Sigma. Paraquat was dissolved in PBS.

### Western blot and antibodies

Cells were harvested at growing stage by regular trypsinization. Cell pellets were re-suspended in 1X RIPA buffer (Cell Signaling) with the volume proportional their cell number followed by vortex, then another volume of 2X Laemmli buffer (Bio-Rad) were added to the cell lysate and boiled for 10 minutes. The following antibodies were used in this study: anti-RNA polymerase II CTD (ab5131), anti-DNAPKcs pS2056 (ab18192), anti-KDM2A (SIGMA SAB2501818), anti-H3K36 di-methyl (ab9049), anti-ORC2 (anti-SIRT6 (ab62739) from Abcam. Anti SIRT6 (12486) from Cell Signaling.

### Plasmids

pCMV-kdm2a-N-terminal FLAG vector was purchased from Genecopoeia (Cat# EX-E1503-Lv102). The mutagenesis was performed using Q5 Site Directed Mutagensis Kit (NEB Cat # E0554S) with primers designed against R1020. For mammalian expression 5 μg of plasmid was incubated with Fugene 6 reagent (E2691) and the mix was used for transfection into 293 cells. The cell lysate was then used for purification by Anti-FLAG M2 Immunoprecipitation kit (SIGMA M8823) according to manufacturer protocol.

### Protein pull-down assay

Protein immunoprecipitations were performed as previously (9). Briefly, MEF cells were harvested and lysed in lysis buffer (20 mM Hepes pH 8.0, 0.2 mM EDTA, 5% glycerol, 150 mM NaCl and 1% NP40) at 4 ^o^C for 10 minutes and sonicated 30 second at 50% duty. The cleared lysate was incubated overnight with 5 μg of antibodies against the indicated proteins, added 25 ul of protein A agarose beads and rotated for another 1 hour. After washing 5 times with the same buffer, the IP was eluted with 2x sample buffer (Laemmli buffer: β-mercapto ethanol=950:50) by boiling for 10 minutes. The eluted samples were spun down and the supernatants were collected for western blotting with the indicated antibodies.

### Retroviral production

Retrovirus was produced by co-transfecting HEK293T cells with pSMP plasmid encoding shRNA against luciferase or Suv39h1 (37) and the packaging plasmids pUMVC and pVSVG. Culture medium containing the recombinant retroviruses was harvested 2 days post transfection, and the virus was concentrated by precipitation with polyethylene glycol 8000. The virus was supplemented with polybrene and incubated with MJ cells for 16 h.

### Chromatin immunoprecipitation and qPCR Detection

ChIP assays were performed as described previously (Mao). Briefly, 3x10^6^ cell equivalents were used for each immunoprecipitation. Of the sample, 1.7% was removed for use as an input control. ChIP was performed following manufacturer’s protocol (Abcam ab500) using antibodies towards KU70 (Abcam EPR4027), SIRT6 (Abcam ab62739), H3K36me2 (Abcam ab9049), phosphorylated RNA polymerase C-terminal domain (ab5131, Abcam) or a control rabbit IgG (ab46540). ChIP-QPCR was performed using Green Master mix (BIORAD 1725270). Primers for b-actin were described previously (Young 2011). Primers that targeted the region flanking the I-SceI cut site was also used (see Supplementary Table 1 for primer sequences). Standard curves were generated for each amplicon using purified human genomic DNA (Clontech). Each experiment was performed at least in triplicate. To quantify KDM2A kinetics on the site of I-SceI cut, quantitative PCR was performed to amplify the region 200 to 500 nt downstream of the TSS site.

### RNA purification and RT-qPCR

Total RNA was isolated from cultured cells at 70% confluency using QIAGEN RNasey mini kit according to manufacturer’s instruction. cDNA was then synthesized using iscript reverse transcriptase (BIORAD 1708840). Real-time quantitative PCR analysis was performed in 20 uL reaction using SYBR Green Master mix (BIORAD 1725270) and CFX Real Time PCR system (BIORAD). Primer efficiency was verified by linear regression to standard curve using 2, 1, ½, ¼, 1/8, 1/16, 1/32, 1/64 and1/128 dilution. Reactions were carried out in quadruplicate and values were standardized to housekeeping genes actin, 18S rRNA, and GAPDH. To normalize values obtained in the samples, control actin Ct values were subtracted from the target gene Ct values for each sample (ΔC_T_) then, Ct of the unstimulated sample was subtracted from ΔC_T_ of the stimulated sample (ΔΔC_T_). The relative levels of target gene were calculated as 2 ^–ΔΔCT^.

### Ribosylation assay

*In vitro* mono-ADP ribosylation assay was conducted as reported previously [23]. Briefly, 5ug of bacterially purified SIRT6 was incubated with 5 μg of the mammalian purified target flag-KDM2A (wild type and R1020W) substrate for 15 minutes at 37 ^o^C.

### Demethylation assay

*In vitro* demethylation assay was conducted as reported previously [54]. Briefly, 100 ng purified active KDM2A (active motif Cat # 31485) was incubated with 100 ng of the synthetic H3K36me2 nucleosome (Epicypher 16-0319) substrate for 15 minutes at 37°.

### Chromatin extraction

Chromatin extraction was performed as previously described [25]. Cells were collected and washed in PBS, and re-suspended in 2-5 pellet volumes of lysis buffer (10mM HEPES pH 7.4, 10mM KCl, 0.05% NP-40 and protease, deacetylase and phosphatase inhibitors). Samples were incubated 20 min on ice, and centrifuged at 14,000 rpm at 4°C for 10 min. The supernatants containing the cytoplasmic proteins were removed and kept in a separate tube. Cell pellets were re-suspended with 2-5 volumes of 0.2N HCl and incubated 20 min on ice, then centrifuged at 14,000 rpm at 4°C for 10 min. Supernatants were kept and neutralized with an equal volume of 1M Tris-HCl pH 8.

### siRNA and plasmid transfection

Mouse embryonic fibroblast (MEF) cells were plated at 500,000 cells per well in 10 cm dishes and grown for at least 48 h in Eagle’s minimum essential medium (EMEM) with Earl’s salts supplemented with 15% fetal calf serum, non-essential amino acids, 1mM sodium pyruvate, and penicillin-streptomycin. Transfection was perfumed using Amaxa Nucleofector machine (Lonza) and the NHDF transfection solution. At 24 h post transfection, the medium was aspirated and replaced with MEM with Earl’s salts supplemented with 15% fetal calf serum, non-essential amino acids, and penicillin-streptomycin. At 16 h after media replacement, cells were treated with Hydrogen peroxide for certain times. Experiments were performed in triplicate, and error bars represent the standard error of the mean.

For siRNA transfection MEF cells were plated at 500,000 cells per well in 10 cm dishes and grown for at least 48 h in Eagle’s minimum essential medium (EMEM) with Earl’s salts supplemented with 15% fetal calf serum, non-essential amino acids, 1mM sodium pyruvate, and penicillin-streptomycin. Cells at 70% confluency were then transfected with 25 nM of indicated siRNA using Amaxa Nucleofector machine (Lonza) and the NHDF transfection solution. All siRNAs were purchased from Dharmacon. Second round of transfection was performed at 48 h. after 24 h the medium was aspirated and replaced with MEM with Earl’s salts supplemented with 15% fetal calf serum, non-essential amino acids, and penicillin-streptomycin. At 16 h after media replacement, cells were treated with Hydrogen peroxide for certain times. Experiments were performed in triplicate, and error bars represent the standard error of the mean.

### Titanium oxide enrichment of ADP-ribosylated peptides

We previously found that SIRT6 ADP-ribosylation activity appears to be as critical as its deacetylase activity in promoting DNA repair [23]. Due to their low abundance, the Leung and Hottiger groups have employed macrodomains to initially enrich for ADP-ribosylated peptides in a “shotgun”-type of approach from whole cells extracts [55]. A potential limitation of this approach is that the macrodomain may impart a bias due to its specificity for only certain residues being modified [56]. Given that ADP ribosylation may occur on several residues, and that a SIRT6 target bias has not been defined, we wanted to employ a method that would be robust in its capacity for enrichment, yet not be biased in ADP-ribosylation sites that could be identified. We chose to employ a phospho-peptide enrichment strategy since the phosphate groups of ribosylated peptides were already show to be enriched by TiO_2_ beads [57]. We employed TiO_2_ beads to enrich for both phosphorylated (and ADP-ribosylated) peptides as previously described [58] with slight modification. We employed mouse embryonic fibroblast (MEF) cells derived from either wild-type or SIRT6^-/-^ mice since any SIRT6-dependent ADP-ribosylation would be absert in the latter. Four T150 flasks grown to approximately 75% confluency were rinsed twice with PBS before trypsin to be sure to avoid carry-over from growth media. Cells from 4 flasks were removed/lysed directly in a total of 3ml of lysis buffer (75 mM NaCl, 3% SDS, 1 mM NaF, 1 mM beta-glycerophosphate, 1 mM sodium orthovanadate, 10 mM sodium pyrophosphate, 1 mM PMSF and 1X Roche Complete Mini EDTA free protease inhibitors in 50 mM HEPES, pH 8.5, and removal from the flask was facilitated by a cell scraper. Lysis was enhanced by passing through a 21gauge needle 20 times followed by homogenization in a Dounce homogenizer, all the while keeping the samples on ice. Next, lysates were sonicated (Branson) with 5x 20 sec pulses at 40% output keeping on ice for one min in between each pulse. Cellular debris was removed by centrifugation at 14,000 rpm for 10 min. The protein concentration of the cell lysates was determined using the BCA assay (Thermo Scientific) after the samples were diluted 10-fold to lower the SDS concentration. Next, extracted proteins were reduced by adding freshly prepared DTT to 10 mM final concentration and samples were incubated at 50°C for 1h. Proteins were allowed to cool briefly on ice and then alkylated by adding iodoacetamide to 15 mM final concentrations and incubated in the dark (wrapped in foil and placed in drawer) for 1h at room temperature (RT). Unreacted iodoacetamide was quenched by adding and additional 10 mM DTT. Next, proteins were precipitated (and SDS removed) with methanol-chloroform. Precipitated pellets were let air-dry overnight in hood at RT. Proteins were resuspended in 1 ml of 50 mM ammonium bicarbonate buffer (pH7.0) by vortexing for several minutes in 30min incremental incubations at 55°C until the pellet appeared to be largely dissolved by the loss of visual aggregates (3h). Samples were allowed to remain at 4°C overnight to help them dissolve further. Proteins were digested with 50 γg of MS-grade trypsin (Thermo-Pierce 100 μg dried trypsin was resuspended in 100μl of 50mM acetic acid and 50μl was added) and incubated on a rotator at 37°C overnight (16-24h). The following morning an additional 50 γg of trypsin was added and incubation continued at 37°C for an additional 8 h. Trypsin was quenched by adding 6ul of 100% TFA (tri-fluoroacetic acid). Peptides were desalted by dividing the sample into 3 480 mg Sep-pak C-18 columns (Waters) to be sure of the capacity. After elution from Sep-pak column with 70% acetonitrile (ACN), peptides were dried in a speedvac in low-bind tubes and care was taken not to over-dry the samples to avoid loss.

Peptides were re-suspended in 0.5 mL of 5% ACN made with MS-grade H_2_O and vortexed for 5 min at RT. The yield of peptides was determined using a peptide fluorometric quantitation assay (Thermo Pierce). Approximately 5 mg was employed for the TiO_2_ enrichment. Samples were adjusted ~1.8ml with a final concentration of 2 M lactic acid and 50% ACN by adding concentrated stocks of each and vortexed for 5 min at RT. Samples were centrifuges at 13,000rpm for 5 min and supernatant was taken leaving behind ~20 γL of sample. Prior to adding to the extract TiO_2_ were prepared by washing sequentially with 1.5 ml of binding buffer: (2M lactic acid, 50% ACN), Elution buffer (50mM KHxPO4, pH10) followed by a final wash with binding buffer. A 4:1 ratio of TiO_2_ beads:peptide was employed for each enrichment. After peptides were mixed with the TiO_2_ beads, the samples were vortexed for 1 h at RT. Samples were centrifuged at 3,000 rpm for 2 min and the unbound fraction (supernatant) was removed and saved. Next, samples were washed three times each in 0.5 ml of binding buffer, then 50% ACN;0.1%TFA and vortexed for 5 min at RT. Each time the samples were centrifuged at 3,000 rpm and the supernatant was removed. ADP-ribosylated and phospho-peptides were eluted with two consecutive 0.5 mL washes with elution buffer and they were pooled. Samples were desalted again with a single SepPak C-18 column (480mg) and dried by speed vac as previously. Peptides were resuspended in 5% ACN, 0.1% formic acid (FA) and subsequently analyzed by nano-electropsray LC/MS/MS on Q-Exactive instrument (Thermo). A 90 min ACN gradient was applied with data-dependent acquisition. Raw files were converted to MGF files and analyzed with Protein Prospector (UCSF).

### DNA double-strand break detection assay

Primers flanking the I-SceI site were used to determine the extent of I-SceI cutting by real-time PCR. Human skin fibroblast cells with stably integrated GFP reporter cassette were mock electroporated or electroporated with I-Sce-I plasmid to the induce DSBs. At different time points after I-Sce-I induction, DNA was extracted and adjusted to equal concentrations. PCR reactions were carried out with CFX Real Time PCR system (BIORAD) and SYBR Green Master mix (BIORAD 1725270). The extent of I-SceI cutting was calculated as the percentage reduction of zero time point after normalization of values to internal control. Quantitative PCR was performed to amplify the region 7 to 315 nt downstream of the I-SceI site with the following primers 5’CCTGAAGATTTGGGGGATTGTGCTTC3’ and 5’CTTGGAAACACCCATGTTGAAATATC3’. As an internal control, primers for the sequence 2 kb away from the I-SceI were included in the PCR analysis.

### Proximity ligation assay

Proximity Ligation Assays (PLA). The mouse/rabbit red starter Duolink kit (Sigma-Aldrich) was used for this experiment. Human skin fibroblast cells were seeded at 3×10^4^ cells per well. The cells were fixed and permeabilized. After permeabilization the cells were incubated in the blocking buffer (provided with the kit) for 1hr at 37 °C in a humidified chamber. Next, cells were incubated with the primary antibodies diluted in the antibody diluents for 1hr at room temperature. Primary antibodies used were mouse anti-HP1α (1:2000; EMD), rabbit anti-yH2AX (1:50; Cell Signaling Technologies), rabbit anti-Suv39h1 (1:800; Abcam). Cells were then washed in Buffer A (supplied with the kit) 2 times for 5min each and incubated with the PLA probes for one hour at 37 °C in a humidified chamber. Cells were washed 2 times for 5min each in Buffer A before the ligation step at 37 °C for one hour in a humid chamber. Following 2 times of 2 min washes, cells were incubated with the amplification mix for 2hr at 37 °C in a darkened humidified chamber. Cells were washed with 1× Buffer B (supplied with the kit) 2 times for 10minutes followed by a 1min wash with 0.01× Buffer B before mounting on cover slips using the mounting media supplied with the kit.

### Mass spectrometry of histones

Histones were extracted, propionylated (pre- and post-digestion), trypsin digested, and processed for mass spectrometry as described previously [59]. NanoLC/Ms/MS was performed as described [60] using a Q-Exactive MS (Thermo) instrument run in data-independent acquisition mode to facilitate quantitation of rare species. The abundance of each peptide was determined from the area of the peak for each histone peptide based upon retention times, as well as parental (MS1) and fragmentation (MS2) m/z values to differentiate those isobaric species which also co-elute during chromatography. Parameters for all peptides were established with stable isotope labeled synthetic standards. Relative abundance of each peptide was calculated with EpiProfile [61], with 10 ppm tolerance for extracting the ion chromatogram. The reported relative fraction of a modified form was calculated by dividing the abundance of a specific modified form by the sum of all forms of the same given peptide, which was considered as 1.

## Supplementary Material

Supplementary Figures

Supplementary Table 1

Supplementary Data 1
