# Supplementary material for: SIRT6 mono-ADP ribosylates KDM2A to locally increase H3K36me2 at DNA damage sites to inhibit transcription and promote repair
Source: Aging (Albany NY). 2020 Jun 25;12(12):11165–84. doi: 10.18632/aging.103567 (PMC7343504; doi:10.18632/aging.103567)
Supplement: Supplementary Figures [file aging-12-103567-s003..pdf]

SUPPLEMENTARY FIGURES

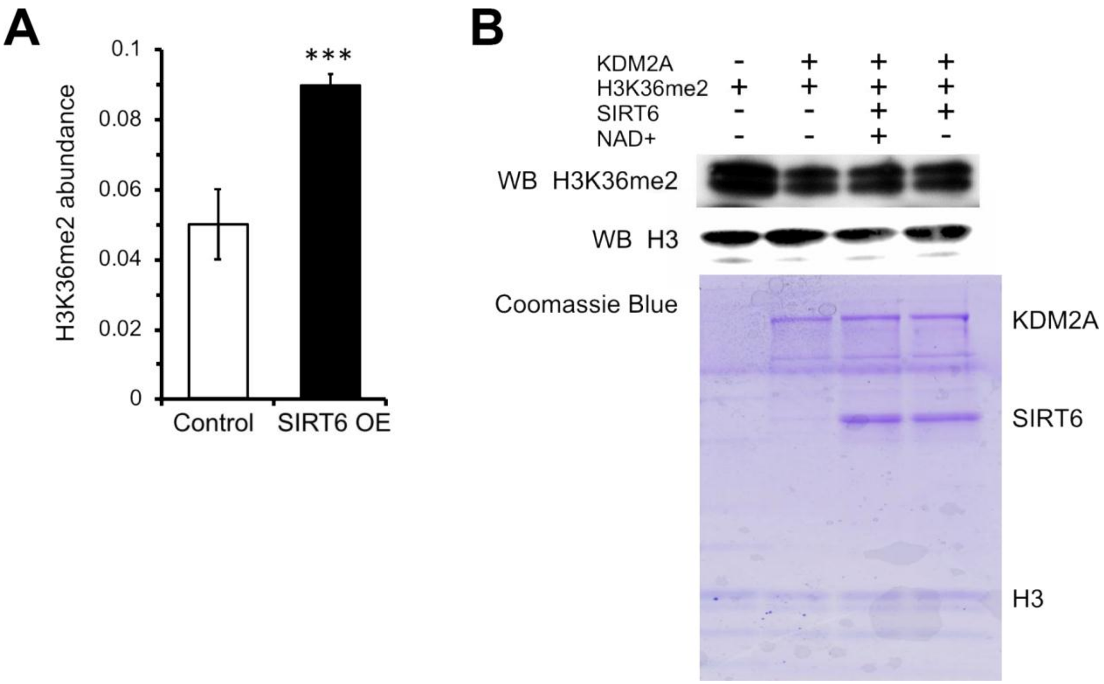

Supplementary Figure 1. The effect of SIRT6 on H3K36 methylation *in vivo* and *in vitro*. (**A**) SIRT6 overexpression (OE) enhances H3K36me2 *in vivo*. Human skin fibroblasts were transduced with SIRT6 or control lentivirus. Cells were harvested after 21 population doublings for histone extraction. MS quantification of total histone modifications showed enrichment of H3K36me2 in cells over expressing SIRT6. ***p < 0.001. (**B**) SIRT6 does not affect KDM2A demethylation activity. (Upper) 100 ng of KDM2A was incubated 100 ng of SIRT6 and 50 ng of designer H3K36me2 mono nucleosomes in the presence or absence of NAD^+ for 1 hour. The reaction was run on the SDS PAGE gel and blotted with total H3 or H3K36me2 antibodies. (Lower) Gel run as above stained with Coomassie blue.

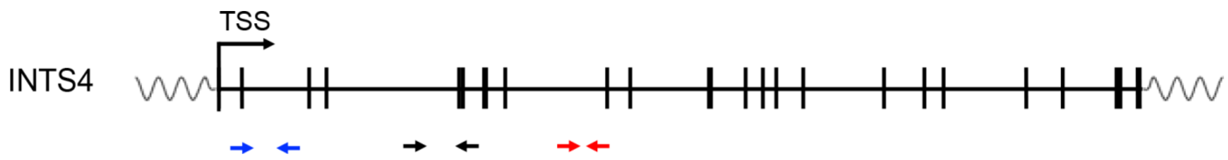

Supplementary Figure 2. Schematic representation of INTS4 (control gene), an RNA Pol II-transcribed gene that does not harbor an ISce-I cleavage site. The primers for quantification of the preRNA (small black arrows), DNA (small red arrows), chromatin IP (blue arrows) are indicated.

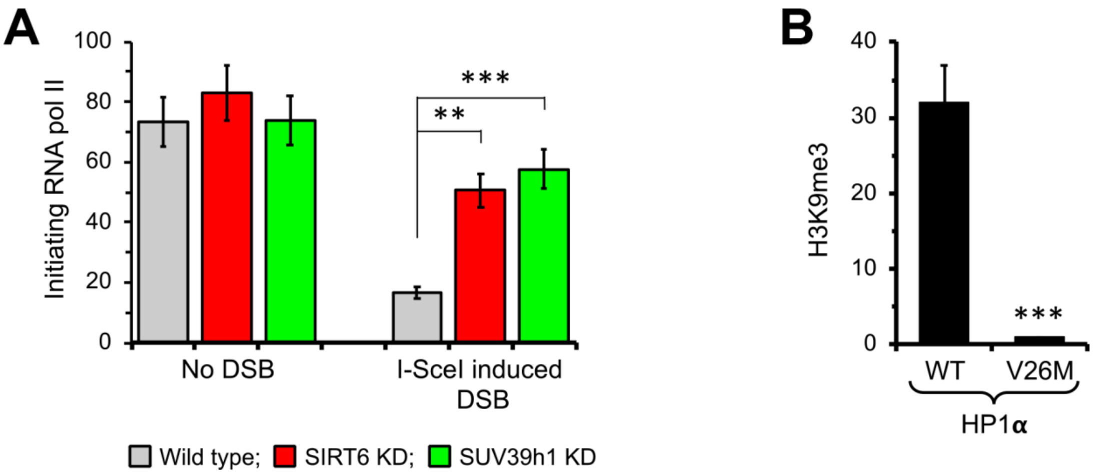

Supplementary Figure 3. (**A**) DSB inhibits initiating RNA polymerase II in SIRT6- and SUV39h1-dependent manner. ChIP assay was performed using anti-S5 antibody 12 h post DSB induction with I-SceI vector. (**B**) HP1α V26M mutant prevents H3K9me3 accumulation at DSB locus. Cells were transfected with I-SceI vector and wild type or mutant flag-HP1α. Cells were then fixed, and ChIP was performed 12 hours post transfection. Bar graph shows enrichment of H3K9me3 on the broken chromatin in cells expressing different HP1α alleles versus uncut cells. The experiments were repeated three times. **$p < 0.01$; ***$p < 0.001$.
