# Supplementary material for: SIRT6 mono-ADP ribosylates KDM2A to locally increase H3K36me2 at DNA damage sites to inhibit transcription and promote repair
Source: Aging (Albany NY). 2020 Jun 25;12(12):11165–84. doi: 10.18632/aging.103567 (PMC7343504; doi:10.18632/aging.103567)
Supplement: Supplementary Table 1 [file aging-12-103567-s001..pdf]

SUPPLEMENTARY TABLE

Supplementary Table 1. Primers used in the study.

I-Sce1cut-f	CCTGAAGATTTGGGGGATTGTGCTTC
I-Sce1cut-r	CTTGGAAACACCCATGTTGAAATATC
INTS4cut-f	GTGGCCCAAGTAGGTCAGAG
INTS4cut-r	GACAAGGCATTTGGCTACCT
GFPexpf	GAAACATTCTTGGACACA
GFPexpr	ATTTTGAAGTTAACTTT
INTSexpf	TGCTTGCAATTGGCACTAAG
INTSexpf	GGACATCTCTGGCAGCTAGG
GFP-TSS-CHIP-f (+25)	TTCACTGGAGTTGTCC
GFP-TSS-CHIP-r (+250)	TCATGCCGTTTCATATGATC
