# Supplementary material for: SIRT6 mono-ADP ribosylates KDM2A to locally increase H3K36me2 at DNA damage sites to inhibit transcription and promote repair
Source: Aging (Albany NY). 2020 Jun 25;12(12):11165–84. doi: 10.18632/aging.103567 (PMC7343504; doi:10.18632/aging.103567)
Supplement: Supplementary Data 1 [file aging-12-103567-s002..pdf]

DPQIR(ADP-Ribosyl)DLLTPPTDKPGQDNRS(Phospho)KLR^{+4}

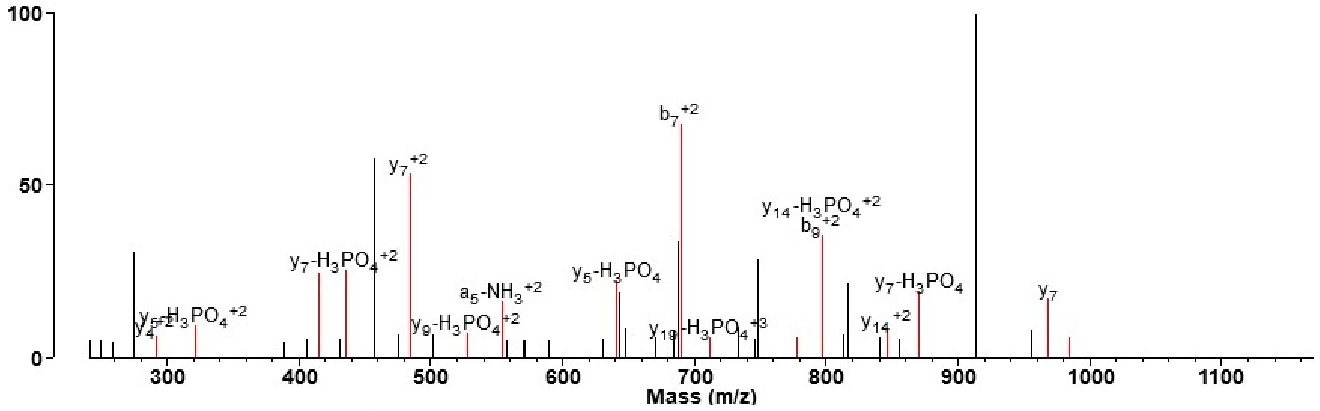

<< < > >> >< <> All **Mass** ☐ **Ser** ☑ **Loss** ☑ **Imm** ☐ **Int** ☐

Max Intensity: **19310**

Num Matched: **16/47 (66.0% unmatched)** Matched Intensity: **43.2%** Matched Series Intensity: **43.2%**

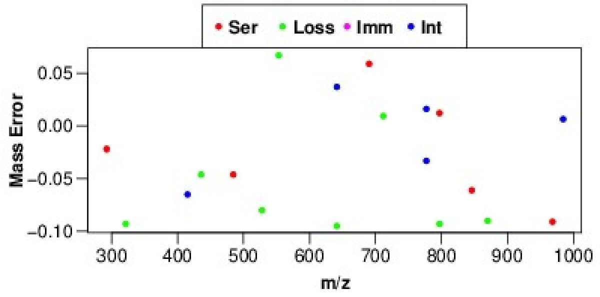

	N Term	Sequence	C Term	N Loss
☑		DPQIR(ADP-Ribosyl)DLLTPPTDKPGQDNRS(Phospho)KLR		

[+] Additional Sequences

Max Charge 4 Count Basic AA **Max Losses** 1 **Multi Z Internal** ☐ **Max Internal Len** 200

Max MSMS Pks 100 **Frag Tol** 0.1 Da **Cal** ☐ **Cal Tol (Da)** 0.1

Filter m/z **to** **z** **to**

MS-Product **Output** HTML

[+] Ion Types

Elemental Composition: C132 H221 N43 O55 P3

MH^{+1}(av)	MH^{+1}(mono)	MH^{+2}(av)	MH^{+2}(mono)	MH^{+3}(av)	MH^{+3}(mono)	MH^{+4}(av)	MH^{+4}(mono)
3383.4042	3381.5026	1692.2058	1691.2549	1128.4730	1127.8390	846.6066	846.1311

[–] Peak Matches

**241.0824**	**250.1193**	**259.0389**	**274.1886**	**292.1295**	**321.1205**	**389.2150**	**406.1742**	**415.0922**	**431.2366**
				y_4^{+2}(-0.022)	y_5-$H_3PO_4^{+2}$(-0.093)			GQDN(-0.065)	
**435.7027**	**457.2296**	**475.2429**	**484.6913**	**502.2736**	**528.2088**	**553.7555**	**557.2502**	**569.7750**	**571.7965**
y_7-$H_3PO_4^{+2}$(-0.046)			y_7^{+2}(-0.046)		y_9-$H_3PO_4^{+2}$(-0.080)	a_5-NH_3^{+2}(0.067)			
**589.2954**	**630.3351**	**641.3258**	**643.6716**	**647.8013**	**670.8206**	**683.9941**	**687.3570**	**689.6689**	**690.3145**
		DKPGQD(0.037)<br>y_5-H_3PO_4(-0.095)							b_7^{+2}(0.059)
**711.7278**	**733.8455**	**746.3691**	**748.3827**	**777.3447**	**797.3332**	**813.6766**	**816.3976**	**840.3340**	**846.3536**
y_{19}-$H_3PO_4^{+3}$(0.0092)				DNRS(Phospho)KL-NH_3(0.016)<br>TPPTDKPG-NH_3(-0.033)	b_9^{+2}(0.012)<br>y_{14}-$H_3PO_4^{+2}$(-0.093)				y_{14}^{+2}(-0.061)
**855.3483**	**870.4001**	**913.4510**	**956.0721**	**968.3766**	**984.4920**	**2097.0225**			

	y_7-H_3PO_4(-0.090)			y_7(-0.091)	TDKPGQDNR-CO(0.0063)	

[–] Main Sequence Ions

b-H_3PO_4	b-$H_3PO_4^{+2}$	b	b^{+2}				y	y^{+2}	y-H_3PO_4	y-$H_3PO_4^{+2}$
---	---	---	---	1	**D**	24	---	---	---	---
---	---	*213.0870*	---	2	**P**	23	*3266.4756*	1633.7414	*3168.4987*	1584.7530
---	---	341.1456	---	3	**Q**	22	*3169.4228*	1585.2151	*3071.4460*	1536.2266
---	---	454.2296	---	4	**I**	21	*3041.3643*	1521.1858	*2943.3874*	1472.1973
---	---	1151.3918	576.1996	5	**R(ADP-Ribosyl)**	20	*2928.2802*	1464.6437	*2830.3033*	1415.6553
---	---	1266.4188	633.7130	6	**D**	19	*2231.1180*	1116.0626	*2133.1411*	1067.0742
---	---	1379.5029	690.2551	7	**L**	18	*2116.0910*	1058.5492	2018.1141	1009.5607
---	---	1492.5869	746.7971	8	**L**	17	2003.0070	1002.0071	1905.0301	953.0187
---	---	1593.6346	797.3209	9	**T**	16	1889.9229	945.4651	1791.9460	896.4766
---	---	1690.6874	845.8473	10	**P**	15	1788.8752	894.9413	1690.8983	845.9528
---	---	1787.7401	894.3737	11	**P**	14	1691.8225	846.4149	1593.8456	797.4264
---	---	1888.7878	944.8975	12	**T**	13	1594.7697	797.8885	1496.7928	748.9000
---	---	2003.8147	1002.4110	13	**D**	12	1493.7220	747.3647	1395.7451	698.3762
---	---	*2131.9097*	1066.4585	14	**K**	11	1378.6951	689.8512	1280.7182	640.8627
---	---	*2228.9625*	1114.9849	15	**P**	10	1250.6001	625.8037	1152.6232	576.8153
---	---	*2285.9839*	1143.4956	16	**G**	9	1153.5474	577.2773	1055.5705	528.2889
---	---	*2414.0425*	1207.5249	17	**Q**	8	1096.5259	548.7666	998.5490	499.7781
---	---	*2529.0695*	1265.0384	18	**D**	7	968.4673	484.7373	870.4904	435.7489
---	---	*2643.1124*	1322.0598	19	**N**	6	853.4404	427.2238	755.4635	378.2354
---	---	*2799.2135*	1400.1104	20	**R**	5	739.3974	370.2024	641.4206	321.2139
2868.2350	1434.6211	*2966.2119*	1483.6096	21	**S(Phospho)**	4	583.2963	292.1518	485.3194	243.1634
2996.3299	1498.6686	*3094.3068*	1547.6570	22	**K**	3	416.2980	*208.6526*	---	---
3109.4140	1555.2106	*3207.3909*	1604.1991	23	**L**	2	288.2030	*144.6051*	---	---
---	---	---	---	24	**R**	1	*175.1190*	*88.0631*	---	---

[–] All Sequence Ions

**MH**	*3381.5026*	1691.2549	1127.8390	846.1311
**MH-H_3PO_4**	*3283.5257*	1642.2665	1095.1801	821.6369
**MH-H_2O**	*3363.4920*	1682.2496	1121.8355	841.6285
**MH-NH_3**	*3364.4760*	1682.7416	1122.1635	841.8745

**Low Mass**	88.0393	70.0651<br>126.0550	84.0444<br>101.0709<br>129.0659	86.0964	70.0651<br>87.0917<br>100.0869<br>112.0869	88.0393	86.0964	86.0964	74.0600	70.0651<br>126.0550	70.0651<br>126.0550	74.0600	88.0393	84.0808<br>101.1073<br>129.1022<br>126.0913	70.0651<br>126.0550	---	84.0444<br>101.0709<br>129.0659	88.0393	87.0553	70.0651<br>87.0917<br>100.0869<br>112.0869	60.0444	84.0808<br>101.1073<br>129.1022<br>126.0913	86.0964	70.0651<br>87.0917<br>100.0869<br>112.0869
**N-terminal**																								
a-NH_3	---	---	296.1241	409.2082	1106.3704	1221.3973	1334.4814	1447.5655	1548.6131	1645.6659	1742.7187	1843.7663	1958.7933	2086.8882	*2183.9410*	*2240.9625*	*2369.0210*	*2484.0480*	*2598.0909*	*2754.1920*	*2921.1904*	*3049.2854*	*3162.3694*	---
a-NH_3^{+2}	---	---	---	---	553.6888	611.2023	667.7443	724.2864	774.8102	823.3366	871.8630	922.3868	979.9003	1043.9478	1092.4741	1120.9849	1185.0142	1242.5276	1299.5491	1377.5997	1461.0988	1525.1463	1581.6883	---
a-NH_3^{+3}	---	---	---	---	---	---	---	---	---	---	---	---	---	696.3009	728.6519	747.6590	790.3452	828.6875	866.7018	918.7355	974.4016	1017.1000	1054.7947	---
a-NH_3^{+4}	---	---	---	---	---	---	---	---	---	---	---	---	---	---	---	---	---	---	---	689.3035	731.0531	763.0768	791.3478	---
a-H_2O	---	*167.0815*	295.1401	408.2241	1105.3864	1220.4133	1333.4974	1446.5814	1547.6291	1644.6819	1741.7346	1842.7823	1957.8093	2085.9042	*2182.9570*	*2239.9785*	*2368.0370*	*2483.0640*	*2597.1069*	*2753.2080*	*2920.2064*	*3048.3013*	*3161.3854*	---

$a-H_2O^{+2}$	---	---	---	---	553.1968	610.7103	667.2523	723.7944	774.3182	822.8446	871.3710	921.8948	979.4083	1043.4558	1091.9821	1120.4929	1184.5222	1242.0356	1299.0571	1377.1076	1460.6068	1524.6543	1581.1963	---
$a-H_2O^{+3}$	---	---	---	---	---	---	---	---	---	---	---	---	---	695.9729	728.3238	747.3310	790.0172	828.3595	866.3738	918.4075	974.0736	1016.7720	1054.4667	---
$a-H_2O^{+4}$	---	---	---	---	---	---	---	---	---	---	---	---	---	---	---	---	---	---	---	689.0575	730.8071	762.8308	791.1018	---
$a-H_3PO_4$	---	---	---	---	---	---	---	---	---	---	---	---	---	---	---	---	---	---	---	---	2840.2400	2968.3350	3081.4191	---
$a-H_3PO_4^{+2}$	---	---	---	---	---	---	---	---	---	---	---	---	---	---	---	---	---	---	---	---	1420.6237	1484.6711	1541.2132	---
$a-H_3PO_4^{+3}$	---	---	---	---	---	---	---	---	---	---	---	---	---	---	---	---	---	---	---	---	947.4182	990.1165	1027.8112	---
$a-H_3PO_4^{+4}$	---	---	---	---	---	---	---	---	---	---	---	---	---	---	---	---	---	---	---	---	710.8155	742.8392	771.1102	---
a	---	185.0921	313.1506	426.2347	1123.3969	1238.4239	1351.5079	1464.5920	1565.6397	1662.6924	1759.7452	1860.7929	1975.8198	2103.9148	2200.9676	2257.9890	2386.0476	2501.0745	2615.1175	2771.2186	2938.2169	3066.3119	3179.3960	---
a^{+2}	---	---	---	---	562.2021	619.7156	676.2576	732.7996	783.3235	831.8499	880.3762	930.9001	988.4136	1052.4610	1100.9874	1129.4981	1193.5274	1251.0409	1308.0624	1386.1129	1469.6121	1533.6596	1590.2016	---
a^{+3}	---	---	---	---	---	---	---	---	---	---	---	---	---	701.9764	734.3274	753.3345	796.0207	834.3630	872.3773	924.4110	980.0772	1022.7755	1060.4702	---
a^{+4}	---	---	---	---	---	---	---	---	---	---	---	---	---	---	---	---	---	---	---	693.5601	735.3097	767.3334	795.6044	---
$b-NH_3$	---	---	324.1190	437.2031	1134.3653	1249.3922	1362.4763	1475.5604	1576.6080	1673.6608	1770.7136	1871.7613	1986.7882	2114.8832	2211.9359	2268.9574	2397.0160	2512.0429	2626.0858	2782.1869	2949.1853	3077.2803	3190.3643	---
$b-NH_3^{+2}$	---	---	---	---	567.6863	625.1998	681.7418	738.2838	788.8077	837.3340	885.8604	936.3843	993.8977	1057.9452	1106.4716	1134.9823	1199.0116	1256.5251	1313.5466	1391.5971	1475.0963	1539.1438	1595.6858	---
$b-NH_3^{+3}$	---	---	---	---	---	---	---	---	---	---	---	---	---	705.6326	737.9835	756.9906	799.6768	838.0192	876.0335	928.0672	983.7333	1026.4316	1064.1263	---
$b-NH_3^{+4}$	---	---	---	---	---	---	---	---	---	---	---	---	---	---	---	---	---	---	---	696.3022	738.0518	770.0755	798.3465	---
$b-H_2O$	---	195.0764	323.1350	436.2191	1133.3813	1248.4082	1361.4923	1474.5764	1575.6240	1672.6768	1769.7296	1870.7772	1985.8042	2113.8991	2210.9519	2267.9734	2396.0319	2511.0589	2625.1018	2781.2029	2948.2013	3076.2963	3189.3803	---
$b-H_2O^{+2}$	---	---	---	---	567.1943	624.7077	681.2498	737.7918	788.3157	836.8420	885.3684	935.8923	993.4057	1057.4532	1105.9796	1134.4903	1198.5196	1256.0331	1313.0545	1391.1051	1474.6043	1538.6518	1595.1938	---
$b-H_2O^{+3}$	---	---	---	---	---	---	---	---	---	---	---	---	---	705.3046	737.6555	756.6626	799.3488	837.6911	875.7055	927.7392	983.4053	1026.1036	1063.7983	---
$b-H_2O^{+4}$	---	---	---	---	---	---	---	---	---	---	---	---	---	---	---	---	---	---	---	696.0562	737.8058	769.8295	798.1005	---
$b-H_3PO_4$	---	---	---	---	---	---	---	---	---	---	---	---	---	---	---	---	---	---	---	---	2868.2350	2996.3299	3109.4140	---
$b-H_3PO_4^{+2}$	---	---	---	---	---	---	---	---	---	---	---	---	---	---	---	---	---	---	---	---	1434.6211	1498.6686	1555.2106	---
$b-H_3PO_4^{+3}$	---	---	---	---	---	---	---	---	---	---	---	---	---	---	---	---	---	---	---	---	956.7498	999.4482	1037.1428	---
$b-H_3PO_4^{+4}$	---	---	---	---	---	---	---	---	---	---	---	---	---	---	---	---	---	---	---	---	717.8142	749.8379	778.1090	---
b	---	213.0870	341.1456	454.2296	1151.3918	1266.4188	1379.5029	1492.5869	1593.6346	1690.6874	1787.7401	1888.7878	2003.8147	2131.9097	2228.9625	2285.9839	2414.0425	2529.0695	2643.1124	2799.2135	2966.2119	3094.3068	3207.3909	---
b^{+2}	---	---	---	---	576.1996	633.7130	690.2551	746.7971	797.3209	845.8473	894.3737	944.8975	1002.4110	1066.4585	1114.9849	1143.4956	1207.5249	1265.0384	1322.0598	1400.1104	1483.6096	1547.6570	1604.1991	---
b^{+3}	---	---	---	---	---	---	---	---	---	---	---	---	---	711.3081	743.6590	762.6662	805.3524	843.6947	881.7090	933.7427	989.4088	1032.1071	1069.8018	---
b^{+4}	---	---	---	---	---	---	---	---	---	---	---	---	---	---	---	---	---	---	---	700.5588	742.3084	774.3322	802.6032	---
$b+H_2O$	---	---	---	---	---	---	---	---	---	---	---	---	---	---	---	---	---	---	---	---	---	3112.3174	3225.4014	---
$b+H_2O^{+2}$	---	---	---	---	---	---	---	---	---	---	---	---	---	---	---	---	---	---	---	---	---	1556.6623	1613.2044	---
$b+H_2O^{+3}$	---	---	---	---	---	---	---	---	---	---	---	---	---	---	---	---	---	---	---	---	---	1038.1106	1075.8053	---
$b+H_2O^{+4}$	---	---	---	---	---	---	---	---	---	---	---	---	---	---	---	---	---	---	---	---	---	778.8348	807.1058	---

	1	2	3	4	5	6	7	8	9	10	11	12	13	14	15	16	17	18	19	20	21	22	23	24	
-	D	P	Q	I	R(ADP-Ribosyl)	D	L	L	T	P	P	T	D	K	P	G	Q	D	N	R	S(Phospho)	K	L	R	-
	24	23	22	21	20	19	18	17	16	15	14	13	12	11	10	9	8	7	6	5	4	3	2	1	

C-terminal

y	---	3266.4756	3169.4228	3041.3643	2928.2802	2231.1180	2116.0910	2003.0070	1889.9229	1788.8752	1691.8225	1594.7697	1493.7220	1378.6951	1250.6001	1153.5474	1096.5259	968.4673	853.4404	739.3974	583.2963	416.2980	288.2030	175.1190
y^{+2}	---	1633.7414	1585.2151	1521.1858	1464.6437	1116.0626	1058.5492	1002.0071	945.4651	894.9413	846.4149	797.8885	747.3647	689.8512	625.8037	577.2773	548.7666	484.7373	427.2238	370.2024	292.1518	208.6526	144.6051	88.0631
y^{+3}	---	1089.4967	1057.1458	1014.4596	976.7649	744.3775	706.0352	668.3405	630.6458	596.9633	564.6123	532.2614	498.5789	460.2365	417.5382	385.1873	366.1801	323.4940	285.1516	247.1373	195.1036	139.4375	---	---
y^{+4}	---	817.3744	793.1112	761.0965	732.8255	558.5350	529.7782	501.5072	473.2362	447.9743	423.7111	399.4479	374.1860	345.4292	313.4055	289.1423	274.8869	242.8723	214.1156	185.6048	---	---	---	---
$y-NH_3$	---	3249.4491	3152.3963	3024.3377	2911.2537	2214.0914	2099.0645	1985.9804	1872.8964	1771.8487	1674.7959	1577.7432	1476.6955	1361.6685	1233.5736	1136.5208	1079.4993	951.4408	836.4138	722.3709	566.2698	399.2714	271.1765	158.0924
$y-NH_3^{+2}$	---	1625.2282	1576.7018	1512.6725	1456.1305	1107.5494	1050.0359	993.4939	936.9518	886.4280	837.9016	789.3752	738.8514	681.3379	617.2904	568.7640	540.2533	476.2240	418.7106	361.6891	283.6385	200.1394	136.0919	79.5498
$y-NH_3^{+3}$	---	1083.8212	1051.4703	1008.7841	971.0894	738.7020	700.3597	662.6650	624.9703	591.2877	558.9368	526.5859	492.9033	454.5610	411.8627	379.5118	360.5046	317.8184	279.4761	241.4618	189.4281	133.7620	---	---
$y-NH_3^{+4}$	---	813.1177	788.8545	756.8399	728.5689	554.2783	525.5216	497.2506	468.9795	443.7176	419.4544	395.1912	369.9293	341.1726	309.1489	284.8857	270.6303	238.6156	209.8589	181.3482	---	---	---	---

y-H_2O	---	3248.4650	3151.4123	3023.3537	2910.2696	2213.1074	2098.0805	1984.9964	1871.9124	1770.8647	1673.8119	1576.7591	1475.7115	1360.6845	1232.5896	1135.5368	1078.5153	950.4568	---	---	---	---	---	---
y-H_2O^{+2}	---	1624.7362	1576.2098	1512.1805	1455.6385	1107.0573	1049.5439	993.0018	936.4598	885.9360	837.4096	788.8832	738.3594	680.8459	616.7984	568.2720	539.7613	475.7320	---	---	---	---	---	---
y-H_2O^{+3}	---	1083.4932	1051.1423	1008.4561	970.7614	738.3740	700.0317	662.3370	624.6423	590.9597	558.6088	526.2579	492.5753	454.2330	411.5347	379.1838	360.1766	317.4904	---	---	---	---	---	---
y-H_2O^{+4}	---	812.8717	788.6085	756.5939	728.3229	554.0323	525.2756	497.0046	468.7335	443.4716	419.2084	394.9452	369.6833	340.9266	308.9028	284.6397	270.3843	238.3696	---	---	---	---	---	---
y-H_3PO_4	---	3168.4987	3071.4460	2943.3874	2830.3033	2133.1411	2018.1141	1905.0301	1791.9460	1690.8983	1593.8456	1496.7928	1395.7451	1280.7182	1152.6232	1055.5705	998.5490	870.4904	755.4635	641.4206	485.3194	---	---	---
y-$H_3PO_4^{+2}$	---	1584.7530	1536.2266	1472.1973	1415.6553	1067.0742	1009.5607	953.0187	896.4766	845.9528	797.4264	748.9000	698.3762	640.8627	576.8153	528.2889	499.7781	435.7489	378.2354	321.2139	243.1634	---	---	---
y-$H_3PO_4^{+3}$	---	1056.8378	1024.4868	981.8006	944.1060	711.7185	673.3762	635.6815	597.9869	564.3043	531.9534	499.6025	465.9199	427.5776	384.8793	352.5283	333.5212	290.8350	252.4927	214.4784	162.4447	---	---	---
y-$H_3PO_4^{+4}$	---	792.8801	768.6169	736.6023	708.3313	534.0407	505.2840	477.0130	448.7420	423.4800	399.2169	374.9537	349.6917	320.9350	288.9113	264.6481	250.3927	218.3781	189.6213	161.1106	---	---	---	---

[–] Internal Ions

Internal Sequence	b	a	b-NH_3	b-H_2O
PG	155.0815	127.0866	---	---
GQ	186.0873	158.0924	169.0608	---
PP	195.1128	167.1179	---	---
PT	199.1077	171.1128	---	181.0972
TP	199.1077	171.1128	---	181.0972
LT	215.1390	187.1441	---	197.1285
TD	217.0819	189.0870	---	199.0713
PQ	226.1186	198.1237	209.0921	---
KP	226.1550	198.1601	209.1285	---
LL	227.1754	199.1805	---	---
DL	229.1183	201.1234	---	211.1077
DN	230.0771	202.0822	213.0506	212.0666
QI	242.1499	214.1550	225.1234	---
KL	242.1863	214.1914	225.1598	---
QD	244.0928	216.0979	227.0662	226.0822
DK	244.1292	216.1343	227.1026	226.1186
NR	271.1513	243.1564	254.1248	---
PGQ	283.1401	255.1452	266.1135	---
KPG	283.1765	255.1816	266.1499	---
S(Phospho)K	296.1006	268.1057	279.0740	278.0900
TPP	296.1605	268.1656	---	278.1499
PPT	296.1605	268.1656	---	278.1499
GQD	301.1143	273.1193	284.0877	283.1037
LTP	312.1918	284.1969	---	294.1812
PTD	314.1347	286.1397	---	296.1241
RS(Phospho)	324.1067	296.1118	307.0802	306.0962
LLT	328.2231	300.2282	---	310.2125
PQI	339.2027	311.2078	322.1761	---
DKP	341.1819	313.1870	324.1554	323.1714
DLL	342.2023	314.2074	---	324.1918
TDK	345.1769	317.1819	328.1503	327.1663
QDN	358.1357	330.1408	341.1092	340.1252
DNR	386.1783	358.1833	369.1517	368.1677
TPPT	397.2082	369.2132	---	379.1976
PGQD	398.1670	370.1721	381.1405	380.1565
DKPG	398.2034	370.2085	381.1769	380.1928
S(Phospho)KL	409.1847	381.1897	392.1581	391.1741
LTPP	409.2445	381.2496	---	391.2340
PPTD	411.1874	383.1925	---	393.1769

**KPGQ**	411.2350	383.2401	394.2085	---
**GQDN**	415.1572	387.1623	398.1306	397.1466
**LLTP**	425.2758	397.2809	---	407.2653
**NRS(Phospho)**	438.1497	410.1548	421.1231	420.1391
**TDKP**	442.2296	414.2347	425.2031	424.2191
**PTDK**	442.2296	414.2347	425.2031	424.2191
**DLLT**	443.2500	415.2551	---	425.2395
**RS(Phospho)K**	452.2017	424.2068	435.1752	434.1911
**TDKPG**	499.2511	471.2562	482.2245	481.2405
**LTPPT**	510.2922	482.2973	---	492.2817
**PGQDN**	512.2100	484.2150	495.1834	494.1994
**TPPTD**	512.2351	484.2402	---	494.2245
**QDNR**	514.2368	486.2419	497.2103	496.2263
**LLTPP**	522.3286	494.3337	---	504.3180
**KPGQD**	526.2620	498.2671	509.2354	508.2514
**DKPGQ**	526.2620	498.2671	509.2354	508.2514
**PTDKP**	539.2824	511.2875	522.2558	521.2718
**PPTDK**	539.2824	511.2875	522.2558	521.2718
**DLLTP**	540.3028	512.3079	---	522.2922
**DNRS(Phospho)**	553.1766	525.1817	536.1501	535.1661
**RS(Phospho)KL**	565.2858	537.2909	548.2592	547.2752
**NRS(Phospho)K**	566.2446	538.2497	549.2181	548.2341
**GQDNR**	571.2583	543.2634	554.2318	553.2477
**PTDKPG**	596.3039	568.3089	579.2773	578.2933
**LLTPPT**	623.3763	595.3814	---	605.3657
**LTPPTD**	625.3192	597.3243	---	607.3086
**TDKPGQ**	627.3097	599.3148	610.2831	609.2991
**PPTDKP**	636.3352	608.3402	619.3086	618.3246
**DLLTPP**	637.3556	609.3606	---	619.3450
**KPGQDN**	640.3049	612.3100	623.2784	622.2944
**TPPTDK**	640.3301	612.3352	623.3035	622.3195
**DKPGQD**	641.2889	613.2940	624.2624	623.2784
**PGQDNR**	668.3111	640.3161	651.2845	650.3005
**NRS(Phospho)KL**	679.3287	651.3338	662.3022	661.3181
**QDNRS(Phospho)**	681.2352	653.2403	664.2086	663.2246
**DNRS(Phospho)K**	681.2716	653.2767	664.2450	663.2610
**PPTDKPG**	693.3566	665.3617	676.3301	675.3461
**PTDKPGQ**	724.3624	696.3675	707.3359	706.3519
**TPPTDKP**	737.3828	709.3879	720.3563	719.3723
**GQDNRS(Phospho)**	738.2567	710.2617	721.2301	720.2461
**DLLTPPT**	738.4032	710.4083	---	720.3927
**LLTPPTD**	738.4032	710.4083	---	720.3927
**TDKPGQD**	742.3366	714.3417	725.3101	724.3260
**LTPPTDK**	753.4141	725.4192	736.3876	735.4036
**DKPGQDN**	755.3319	727.3369	738.3053	737.3213
**DNRS(Phospho)KL**	794.3556	766.3607	777.3291	776.3451
**TPPTDKPG**	794.4043	766.4094	777.3777	776.3937
**KPGQDNR**	796.4060	768.4111	779.3795	778.3955
**QDNRS(Phospho)K**	809.3302	781.3352	792.3036	791.3196
**IR(ADP-Ribosyl)**	811.2536	783.2586	794.2270	---
**R(ADP-Ribosyl)D**	813.1964	785.2015	796.1699	795.1859
**PPTDKPGQ**	821.4152	793.4203	804.3886	803.4046

PGQDNRS(Phospho)	835.3094	807.3145	818.2829	817.2989
PTDKPGQD	839.3894	811.3945	822.3628	821.3788
LTPPTDKP	850.4669	822.4720	833.4403	832.4563
DLLTPPTD	853.4302	825.4353	---	835.4196
TDKPGQDN	856.3795	828.3846	839.3530	838.3690
GQDNRS(Phospho)K	866.3516	838.3567	849.3251	848.3411
LLTPPTDK	866.4982	838.5033	849.4716	848.4876
LTPPTDKPG	907.4884	879.4934	890.4618	889.4778
DKPGQDNR	911.4330	883.4381	894.4064	893.4224
QDNRS(Phospho)KL	922.4142	894.4193	905.3877	904.4037
TPPTDKPGQ	922.4629	894.4680	905.4363	904.4523
R(ADP-Ribosyl)DL	926.2805	898.2856	909.2540	908.2699
IR(ADP-Ribosyl)D	926.2805	898.2856	909.2540	908.2699
PPTDKPGQD	936.4421	908.4472	919.4156	918.4316
QIR(ADP-Ribosyl)	939.3121	911.3172	922.2856	---
PTDKPGQDN	953.4323	925.4374	936.4058	935.4217
PGQDNRS(Phospho)K	963.4044	935.4095	946.3778	945.3938
KPGQDNRS(Phospho)	963.4044	935.4095	946.3778	945.3938
LLTPPTDKP	963.5510	935.5560	946.5244	945.5404
GQDNRS(Phospho)KL	979.4357	951.4408	962.4091	961.4251
DLLTPPTDK	981.5251	953.5302	964.4986	963.5146
TDKPGQDNR	1012.4806	984.4857	995.4541	994.4701
LLTPPTDKPG	1020.5724	992.5775	1003.5459	1002.5619
LTPPTDKPGQ	1035.5469	1007.5520	1018.5204	1017.5364
PQIR(ADP-Ribosyl)	1036.3649	1008.3700	1019.3384	---
TPPTDKPGQD	1037.4898	1009.4949	1020.4633	1019.4793
R(ADP-Ribosyl)DLL	1039.3646	1011.3697	1022.3380	1021.3540
IR(ADP-Ribosyl)DL	1039.3646	1011.3697	1022.3380	1021.3540
PPTDKPGQDN	1050.4851	1022.4901	1033.4585	1032.4745
QIR(ADP-Ribosyl)D	1054.3391	1026.3442	1037.3125	1036.3285
PGQDNRS(Phospho)KL	1076.4884	1048.4935	1059.4619	1058.4779
DKPGQDNRS(Phospho)	1078.4313	1050.4364	1061.4048	1060.4208
DLLTPPTDKP	1078.5779	1050.5830	1061.5514	1060.5673
KPGQDNRS(Phospho)K	1091.4993	1063.5044	1074.4728	1073.4888
PTDKPGQDNR	1109.5334	1081.5385	1092.5069	1091.5228
DLLTPPTDKPG	1135.5994	1107.6045	1118.5728	1117.5888
R(ADP-Ribosyl)DLLT	1140.4122	1112.4173	1123.3857	1122.4017
LLTPPTDKPGQ	1148.6310	1120.6361	1131.6045	1130.6204
LTPPTDKPGQD	1150.5739	1122.5790	1133.5473	1132.5633
PQIR(ADP-Ribosyl)D	1151.3918	1123.3969	1134.3653	1133.3813
TPPTDKPGQDN	1151.5327	1123.5378	1134.5062	1133.5222
IR(ADP-Ribosyl)DLL	1152.4486	1124.4537	1135.4221	1134.4381
QIR(ADP-Ribosyl)DL	1167.4231	1139.4282	1150.3966	1149.4126
TDKPGQDNRS(Phospho)	1179.4790	1151.4841	1162.4525	1161.4684
KPGQDNRS(Phospho)KL	1204.5834	1176.5885	1187.5569	1186.5728
DKPGQDNRS(Phospho)K	1206.5263	1178.5314	1189.4997	1188.5157
PPTDKPGQDNR	1206.5862	1178.5913	1189.5596	1188.5756
R(ADP-Ribosyl)DLLTP	1237.4650	1209.4701	1220.4385	1219.4544
IR(ADP-Ribosyl)DLLT	1253.4963	1225.5014	1236.4698	1235.4857
LLTPPTDKPGQD	1263.6579	1235.6630	1246.6314	1245.6474
DLLTPPTDKPGQ	1263.6579	1235.6630	1246.6314	1245.6474
PQIR(ADP-Ribosyl)DL	1264.4759	1236.4810	1247.4494	1246.4653

**LTPPTDKPGQDN**	1264.6168	1236.6219	1247.5903	1246.6062
**PTDKPGQDNRS(Phospho)**	1276.5318	1248.5369	1259.5052	1258.5212
**QIR(ADP-Ribosyl)DLL**	1280.5072	1252.5123	1263.4807	1262.4966
**TDKPGQDNRS(Phospho)K**	1307.5740	1279.5791	1290.5474	1289.5634
**TPPTDKPGQDNR**	1307.6339	1279.6389	1290.6073	1289.6233
**DKPGQDNRS(Phospho)KL**	1319.6104	1291.6154	1302.5838	1301.5998
**R(ADP-Ribosyl)DLLTPP**	1334.5178	1306.5229	1317.4912	1316.5072
**IR(ADP-Ribosyl)DLLTP**	1350.5491	1322.5542	1333.5225	1332.5385
**PPTDKPGQDNRS(Phospho)**	1373.5845	1345.5896	1356.5580	1355.5740
**PQIR(ADP-Ribosyl)DLL**	1377.5600	1349.5651	1360.5334	1359.5494
**LLTPPTDKPGQDN**	1377.7009	1349.7060	1360.6743	1359.6903
**DLLTPPTDKPGQD**	1378.6849	1350.6900	1361.6583	1360.6743
**QIR(ADP-Ribosyl)DLLT**	1381.5549	1353.5600	1364.5283	1363.5443
**PTDKPGQDNRS(Phospho)K**	1404.6267	1376.6318	1387.6002	1386.6162
**TDKPGQDNRS(Phospho)KL**	1420.6580	1392.6631	1403.6315	1402.6475
**LTPPTDKPGQDNR**	1420.7179	1392.7230	1403.6914	1402.7074
**R(ADP-Ribosyl)DLLTPPT**	1435.5655	1407.5705	1418.5389	1417.5549
**IR(ADP-Ribosyl)DLLTPP**	1447.6018	1419.6069	1430.5753	1429.5913
**TPPTDKPGQDNRS(Phospho)**	1474.6322	1446.6373	1457.6057	1456.6216
**QIR(ADP-Ribosyl)DLLTP**	1478.6077	1450.6127	1461.5811	1460.5971
**PQIR(ADP-Ribosyl)DLLT**	1478.6077	1450.6127	1461.5811	1460.5971
**DLLTPPTDKPGQDN**	1492.7278	1464.7329	1475.7013	1474.7172
**PPTDKPGQDNRS(Phospho)K**	1501.6795	1473.6846	1484.6529	1483.6689
**PTDKPGQDNRS(Phospho)KL**	1517.7108	1489.7159	1500.6842	1499.7002
**LLTPPTDKPGQDNR**	1533.8020	1505.8071	1516.7754	1515.7914
**IR(ADP-Ribosyl)DLLTPPT**	1548.6495	1520.6546	1531.6230	1530.6390
**R(ADP-Ribosyl)DLLTPPTD**	1550.5924	1522.5975	1533.5658	1532.5818
**PQIR(ADP-Ribosyl)DLLTP**	1575.6604	1547.6655	1558.6339	1557.6499
**QIR(ADP-Ribosyl)DLLTPP**	1575.6604	1547.6655	1558.6339	1557.6499
**LTPPTDKPGQDNRS(Phospho)**	1587.7163	1559.7214	1570.6897	1569.7057
**TPPTDKPGQDNRS(Phospho)K**	1602.7272	1574.7323	1585.7006	1584.7166
**PPTDKPGQDNRS(Phospho)KL**	1614.7636	1586.7686	1597.7370	1596.7530
**DLLTPPTDKPGQDNR**	1648.8289	1620.8340	1631.8024	1630.8184
**IR(ADP-Ribosyl)DLLTPPTD**	1663.6765	1635.6815	1646.6499	1645.6659
**PQIR(ADP-Ribosyl)DLLTPP**	1672.7132	1644.7183	1655.6866	1654.7026
**QIR(ADP-Ribosyl)DLLTPPT**	1676.7081	1648.7132	1659.6815	1658.6975
**R(ADP-Ribosyl)DLLTPPTDK**	1678.6874	1650.6924	1661.6608	1660.6768
**LLTPPTDKPGQDNRS(Phospho)**	1700.8003	1672.8054	1683.7738	1682.7898
**TPPTDKPGQDNRS(Phospho)KL**	1715.8112	1687.8163	1698.7847	1697.8007
**LTPPTDKPGQDNRS(Phospho)K**	1715.8112	1687.8163	1698.7847	1697.8007
**PQIR(ADP-Ribosyl)DLLTPPT**	1773.7609	1745.7659	1756.7343	1755.7503
**R(ADP-Ribosyl)DLLTPPTDKP**	1775.7401	1747.7452	1758.7136	1757.7296
**QIR(ADP-Ribosyl)DLLTPPTD**	1791.7350	1763.7401	1774.7085	1773.7245
**IR(ADP-Ribosyl)DLLTPPTDK**	1791.7714	1763.7765	1774.7449	1773.7609
**DLLTPPTDKPGQDNRS(Phospho)**	1815.8273	1787.8324	1798.8007	1797.8167
**LTPPTDKPGQDNRS(Phospho)KL**	1828.8953	1800.9004	1811.8688	1810.8847
**LLTPPTDKPGQDNRS(Phospho)K**	1828.8953	1800.9004	1811.8688	1810.8847
**R(ADP-Ribosyl)DLLTPPTDKPG**	1832.7616	1804.7667	1815.7350	1814.7510
**PQIR(ADP-Ribosyl)DLLTPPTD**	1888.7878	1860.7929	1871.7613	1870.7772
**IR(ADP-Ribosyl)DLLTPPTDKP**	1888.8242	1860.8293	1871.7976	1870.8136
**QIR(ADP-Ribosyl)DLLTPPTDK**	1919.8300	1891.8351	1902.8035	1901.8194
**LLTPPTDKPGQDNRS(Phospho)KL**	1941.9794	1913.9845	1924.9528	1923.9688

**DLLTPPTDKPGQDNRS(Phospho)K**	1943.9222	1915.9273	1926.8957	1925.9117
**IR(ADP-Ribosyl)DLLTPPTDKPG**	1945.8456	1917.8507	1928.8191	1927.8351
**R(ADP-Ribosyl)DLLTPPTDKPGQ**	1960.8202	1932.8252	1943.7936	1942.8096
**QIR(ADP-Ribosyl)DLLTPPTDKP**	2016.8828	1988.8878	1999.8562	1998.8722
**PQIR(ADP-Ribosyl)DLLTPPTDK**	2016.8828	1988.8878	1999.8562	1998.8722
**DLLTPPTDKPGQDNRS(Phospho)KL**	2057.0063	2029.0114	2039.9798	2038.9957
**IR(ADP-Ribosyl)DLLTPPTDKPGQ**	2073.9042	2045.9093	2056.8777	2055.8937
**QIR(ADP-Ribosyl)DLLTPPTDKPG**	2073.9042	2045.9093	2056.8777	2055.8937
**R(ADP-Ribosyl)DLLTPPTDKPGQD**	2075.8471	2047.8522	2058.8206	2057.8365
**PQIR(ADP-Ribosyl)DLLTPPTDKP**	*2113.9355*	2085.9406	2096.9090	2095.9250
**PQIR(ADP-Ribosyl)DLLTPPTDKPG**	*2170.9570*	*2142.9621*	*2153.9304*	*2152.9464*
**IR(ADP-Ribosyl)DLLTPPTDKPGQD**	*2188.9312*	*2160.9363*	*2171.9046*	*2170.9206*
**R(ADP-Ribosyl)DLLTPPTDKPGQDN**	*2189.8900*	*2161.8951*	*2172.8635*	*2171.8795*
**QIR(ADP-Ribosyl)DLLTPPTDKPGQ**	*2201.9628*	*2173.9679*	*2184.9363*	*2183.9522*
**PQIR(ADP-Ribosyl)DLLTPPTDKPGQ**	*2299.0156*	*2271.0207*	*2281.9890*	*2281.0050*
**IR(ADP-Ribosyl)DLLTPPTDKPGQDN**	*2302.9741*	*2274.9792*	*2285.9475*	*2284.9635*
**QIR(ADP-Ribosyl)DLLTPPTDKPGQD**	*2316.9897*	*2288.9948*	*2299.9632*	*2298.9792*
**R(ADP-Ribosyl)DLLTPPTDKPGQDNR**	*2345.9911*	*2317.9962*	*2328.9646*	*2327.9806*
**PQIR(ADP-Ribosyl)DLLTPPTDKPGQD**	*2414.0425*	*2386.0476*	*2397.0160*	*2396.0319*
**QIR(ADP-Ribosyl)DLLTPPTDKPGQDN**	*2431.0327*	*2403.0378*	*2414.0061*	*2413.0221*
**IR(ADP-Ribosyl)DLLTPPTDKPGQDNR**	*2459.0752*	*2431.0803*	*2442.0487*	*2441.0646*
**R(ADP-Ribosyl)DLLTPPTDKPGQDNRS(Phospho)**	*2512.9895*	*2484.9946*	*2495.9630*	*2494.9789*
**PQIR(ADP-Ribosyl)DLLTPPTDKPGQDN**	*2528.0854*	*2500.0905*	*2511.0589*	*2510.0749*
**QIR(ADP-Ribosyl)DLLTPPTDKPGQDNR**	*2587.1338*	*2559.1389*	*2570.1072*	*2569.1232*
**IR(ADP-Ribosyl)DLLTPPTDKPGQDNRS(Phospho)**	*2626.0736*	*2598.0787*	*2609.0470*	*2608.0630*
**R(ADP-Ribosyl)DLLTPPTDKPGQDNRS(Phospho)K**	*2641.0845*	*2613.0896*	*2624.0579*	*2623.0739*
**PQIR(ADP-Ribosyl)DLLTPPTDKPGQDNR**	*2684.1865*	*2656.1916*	*2667.1600*	*2666.1760*
**QIR(ADP-Ribosyl)DLLTPPTDKPGQDNRS(Phospho)**	*2754.1321*	*2726.1372*	*2737.1056*	*2736.1216*
**IR(ADP-Ribosyl)DLLTPPTDKPGQDNRS(Phospho)K**	*2754.1685*	*2726.1736*	*2737.1420*	*2736.1580*
**R(ADP-Ribosyl)DLLTPPTDKPGQDNRS(Phospho)KL**	*2754.1685*	*2726.1736*	*2737.1420*	*2736.1580*
**PQIR(ADP-Ribosyl)DLLTPPTDKPGQDNRS(Phospho)**	*2851.1849*	*2823.1900*	*2834.1584*	*2833.1743*
**IR(ADP-Ribosyl)DLLTPPTDKPGQDNRS(Phospho)KL**	*2867.2526*	*2839.2577*	*2850.2260*	*2849.2420*
**QIR(ADP-Ribosyl)DLLTPPTDKPGQDNRS(Phospho)K**	*2882.2271*	*2854.2322*	*2865.2006*	*2864.2165*
**PQIR(ADP-Ribosyl)DLLTPPTDKPGQDNRS(Phospho)K**	*2979.2799*	*2951.2850*	*2962.2533*	*2961.2693*
**QIR(ADP-Ribosyl)DLLTPPTDKPGQDNRS(Phospho)KL**	*2995.3112*	*2967.3163*	*2978.2846*	*2977.3006*
**PQIR(ADP-Ribosyl)DLLTPPTDKPGQDNRS(Phospho)KL**	*3092.3639*	*3064.3690*	*3075.3374*	*3074.3534*

[–] Theoretical Peak Table

60.0444	**S**	C2 H6 N1 O1	736.6023^{+4}	**y_{21}-$H_3PO_4^{+4}$**	C118 H198 N39 O45 P2	1351.5079	**a_7**	C50 H81 N16 O24 P2
70.0651	**P**	C4 H8 N1	737.3213	**DKPGQDN-H_2O**	C30 H45 N10 O12	1353.5600	**QIR(ADP-Ribosyl)DLLT-CO**	C51 H87 N16 O23 P2
70.0651	**R**	C4 H8 N1	737.3828	**TPPTDKP**	C33 H53 N8 O11	1355.5740	**PPTDKPGQDNRS(Phospho)-H_2O**	C53 H84 N18 O22 P1
74.0600	**T**	C3 H8 N1 O1	737.6555^{+3}	**b_{15}-H_2O^{+3}**	C90 H142 N25 O36 P2	1356.5580	**PPTDKPGQDNRS(Phospho)-NH_3**	C53 H83 N17 O23 P1
79.5498^{+2}	**y_1-NH_3^{+2}**	C6 H12 N3 O2	737.7918^{+2}	**b_8-H_2O^{+2}**	C57 H90 N17 O25 P2	1359.5494	**PQIR(ADP-Ribosyl)DLL-H_2O**	C53 H85 N16 O22 P2
84.0444	**Q**	C4 H6 N1 O1	737.8058^{+4}	**b_{21}-H_2O^{+4}**	C114 H182 N36 O50 P3	1359.6903	**LLTPPTDKPGQDN-H_2O**	C60 H95 N16 O20
84.0808	**K**	C5 H10 N1	737.9835^{+3}	**b_{15}-NH_3^{+3}**	C90 H141 N24 O37 P2	1360.5334	**PQIR(ADP-Ribosyl)DLL-NH_3**	C53 H84 N15 O23 P2
86.0964	**I**	C5 H12 N1	738.0518^{+4}	**b_{21}-NH_3^{+4}**	C114 H181 N35 O51 P3	1360.6743	**LLTPPTDKPGQDN-NH_3**	C60 H94 N15 O21
86.0964	**L**	C5 H12 N1	738.2567	**GQDNRS(Phospho)**	C24 H41 N11 O14 P1	1360.6743	**DLLTPPTDKPGQD-H_2O**	C60 H94 N15 O21
87.0553	**N**	C3 H7 N2 O1	738.2838^{+2}	**b_8-NH_3^{+2}**	C57 H89 N16 O26 P2	1360.6845	**y_{11}-H_2O**	C53 H95 N21 O19 P1
87.0917	**R**	C4 H11 N2	738.3053	**DKPGQDN-NH_3**	C30 H44 N9 O13	1361.4923	**b_7-H_2O**	C51 H79 N16 O24 P2

88.0393	**D**	C3 H6 N1 O2	738.3594^{+2}	**y_{12}-H_2O^{+2}**	C57 H100 N22 O22 P1	1361.6583	**DLLTPPTDKPGQD-NH_3**	C60 H93 N14 O22
88.0631^{+2}	**y_1^{+2}**	C6 H15 N4 O2	738.3740^{+3}	**y_{19}-H_2O^{+3}**	C91 H155 N29 O33 P1	1361.6685	**y_{11}-NH_3**	C53 H94 N20 O20 P1
100.0869	**R**	C4 H10 N3	738.4032	**DLLTPPT**	C34 H56 N7 O11	1362.4763	**b_7-NH_3**	C51 H78 N15 O25 P2
101.0709	**Q**	C4 H9 N2 O1	738.4032	**LLTPPTD**	C34 H56 N7 O11	1363.5443	**QIR(ADP-Ribosyl)DLLT-H_2O**	C52 H85 N16 O23 P2
101.1073	**K**	C5 H13 N2	738.7020^{+3}	**y_{19}-NH_3^{+3}**	C91 H154 N28 O34 P1	1364.5283	**QIR(ADP-Ribosyl)DLLT-NH_3**	C52 H84 N15 O24 P2
112.0869	**R**	C5 H10 N3	738.8514^{+2}	**y_{12}-NH_3^{+2}**	C57 H99 N21 O23 P1	1373.5845	**PPTDKPGQDNRS(Phospho)**	C53 H86 N18 O23 P1
126.0550	**P**	C6 H8 N1 O2	739.3974	**y_5**	C27 H56 N12 O10 P1	1376.6318	**PTDKPGQDNRS(Phospho)K-CO**	C53 H91 N19 O22 P1
126.0913	**K**	C7 H12 N1 O1	742.3084^{+4}	**b_{21}^{+4}**	C114 H184 N36 O51 P3	1377.1076^{+2}	**a_{20}-H_2O^{+2}**	C110 H176 N35 O44 P2
127.0866	**PG-CO**	C6 H11 N2 O1	742.3366	**TDKPGQD**	C30 H48 N9 O13	1377.5600	**PQIR(ADP-Ribosyl)DLL**	C53 H87 N16 O23 P2
129.0659	**Q**	C5 H9 N2 O2	742.8392^{+4}	**a_{22}-$H_3PO_4^{+4}$**	C119 H193 N38 O47 P2	1377.5997^{+2}	**a_{20}-NH_3^{+2}**	C110 H175 N34 O45 P2
129.1022	**K**	C6 H13 N2 O1	743.6590^{+3}	**b_{15}^{+3}**	C90 H144 N25 O37 P2	1377.7009	**LLTPPTDKPGQDN**	C60 H97 N16 O21
133.7620^{+3}	**y_3-NH_3^{+3}**	C18 H35 N6 O4	744.3775^{+3}	**y_{19}^{+3}**	C91 H157 N29 O34 P1	1378.6849	**DLLTPPTDKPGQD**	C60 H96 N15 O22
136.0919^{+2}	**y_2-NH_3^{+2}**	C12 H23 N4 O3	746.7971^{+2}	**b_8^{+2}**	C57 H92 N17 O26 P2	1378.6951	**y_{11}**	C53 H97 N21 O20 P1
139.4375^{+3}	**y_3^{+3}**	C18 H38 N7 O4	747.3310^{+3}	**a_{16}-H_2O^{+3}**	C91 H145 N26 O36 P2	1379.5029	**b_7**	C51 H81 N16 O25 P2
144.6051^{+2}	**y_2^{+2}**	C12 H26 N5 O3	747.3647^{+2}	**y_{12}^{+2}**	C57 H102 N22 O23 P1	1381.5549	**QIR(ADP-Ribosyl)DLLT**	C52 H87 N16 O24 P2
155.0815	**PG**	C7 H11 N2 O2	747.6590^{+3}	**a_{16}-NH_3^{+3}**	C91 H144 N25 O37 P2	1386.1129^{+2}	**a_{20}^{+2}**	C110 H178 N35 O45 P2
158.0924	**GQ-CO**	C6 H12 N3 O2	748.9000^{+2}	**y_{13}-$H_3PO_4^{+2}$**	C61 H106 N23 O21	1386.6162	**PTDKPGQDNRS(Phospho)K-H_2O**	C54 H89 N19 O22 P1
158.0924	**y_1-NH_3**	C6 H12 N3 O2	749.8379^{+4}	**b_{22}-$H_3PO_4^{+4}$**	C120 H193 N38 O48 P2	1387.6002	**PTDKPGQDNRS(Phospho)K-NH_3**	C54 H88 N18 O23 P1
161.1106^{+4}	**y_5-$H_3PO_4^{+4}$**	C27 H53 N12 O6	753.3345^{+3}	**a_{16}^{+3}**	C91 H147 N26 O37 P2	1391.1051^{+2}	**b_{20}-H_2O^{+2}**	C111 H176 N35 O45 P2
162.4447^{+3}	**y_4-$H_3PO_4^{+3}$**	C21 H41 N8 O5	753.4141	**LTPPTDK**	C34 H57 N8 O11	1391.5971^{+2}	**b_{20}-NH_3^{+2}**	C111 H175 N34 O46 P2
167.0815	**a_2-H_2O**	C8 H11 N2 O2	755.3319	**DKPGQDN**	C30 H47 N10 O13	1392.6631	**TDKPGQDNRS(Phospho)KL-CO**	C54 H95 N19 O22 P1
167.1179	**PP-CO**	C9 H15 N2 O1	755.4635	**y_6-H_3PO_4**	C31 H59 N14 O8	1392.7230	**LTPPTDKPGQDNR-CO**	C59 H98 N19 O20
169.0608	**GQ-NH_3**	C7 H9 N2 O3	756.5939^{+4}	**y_{21}-H_2O^{+4}**	C118 H199 N39 O48 P3	1395.7451	**y_{12}-H_3PO_4**	C57 H99 N22 O19
171.1128	**PT-CO**	C8 H15 N2 O2	756.6626^{+3}	**b_{16}-H_2O^{+3}**	C92 H145 N26 O37 P2	1400.1104^{+2}	**b_{20}^{+2}**	C111 H178 N35 O46 P2
171.1128	**TP-CO**	C8 H15 N2 O2	756.8399^{+4}	**y_{21}-NH_3^{+4}**	C118 H198 N38 O49 P3	1402.6475	**TDKPGQDNRS(Phospho)KL-H_2O**	C55 H93 N19 O22 P1
175.1190	**y_1**	C6 H15 N4 O2	756.9906^{+3}	**b_{16}-NH_3^{+3}**	C92 H144 N25 O38 P2	1402.7074	**LTPPTDKPGQDNR-H_2O**	C60 H96 N19 O20
181.0972	**PT-H_2O**	C9 H13 N2 O2	761.0965^{+4}	**y_{21}^{+4}**	C118 H201 N39 O49 P3	1403.6315	**TDKPGQDNRS(Phospho)KL-NH_3**	C55 H92 N18 O23 P1
181.0972	**TP-H_2O**	C9 H13 N2 O2	762.6662^{+3}	**b_{16}^{+3}**	C92 H147 N26 O38 P2	1403.6914	**LTPPTDKPGQDNR-NH_3**	C60 H95 N18 O21
181.3482^{+4}	**y_5-NH_3^{+4}**	C27 H53 N11 O10 P1	762.8308^{+4}	**a_{22}-H_2O^{+4}**	C119 H194 N38 O50 P3	1404.6267	**PTDKPGQDNRS(Phospho)K**	C54 H91 N19 O23 P1
185.0921	**a_2**	C8 H13 N2 O3	763.0768^{+4}	**a_{22}-NH_3^{+4}**	C119 H193 N37 O51 P3	1407.5705	**R(ADP-Ribosyl)DLLTPPT-CO**	C54 H89 N16 O24 P2
185.6048^{+4}	**y_5^{+4}**	C27 H56 N12 O10 P1	766.3607	**DNRS(Phospho)KL-CO**	C28 H53 N11 O12 P1	1415.6553^{+2}	**y_{20}-$H_3PO_4^{+2}$**	C112 H187 N38 O44 P2
186.0873	**GQ**	C7 H12 N3 O3	766.4094	**TPPTDKPG-CO**	C34 H56 N9 O11	1417.5549	**R(ADP-Ribosyl)DLLTPPT-H_2O**	C55 H87 N16 O24 P2
187.1441	**LT-CO**	C9 H19 N2 O2	767.3334^{+4}	**a_{22}^{+4}**	C119 H196 N38 O51 P3	1418.5389	**R(ADP-Ribosyl)DLLTPPT-NH_3**	C55 H86 N15 O25 P2
189.0870	**TD-CO**	C7 H13 N2 O4	768.4111	**KPGQDNR-CO**	C31 H54 N13 O10	1419.6069	**IR(ADP-Ribosyl)DLLTPP-CO**	C56 H93 N16 O23 P2
189.4281^{+3}	**y_4-NH_3^{+3}**	C21 H41 N7 O9 P1	768.6169^{+4}	**y_{22}-$H_3PO_4^{+4}$**	C123 H206 N41 O47 P2	1420.6237^{+2}	**a_{21}-$H_3PO_4^{+2}$**	C113 H181 N36 O46 P2
189.6213^{+4}	**y_6-$H_3PO_4^{+4}$**	C31 H59 N14 O8	769.8295^{+4}	**b_{22}-H_2O^{+4}**	C120 H194 N38 O51 P3	1420.6580	**TDKPGQDNRS(Phospho)KL**	C55 H95 N19 O23 P1
195.0764	**b_2-H_2O**	C9 H11 N2 O3	770.0755^{+4}	**b_{22}-NH_3^{+4}**	C120 H193 N37 O52 P3	1420.7179	**LTPPTDKPGQDNR**	C60 H98 N19 O21
195.1036^{+3}	**y_4^{+3}**	C21 H44 N8 O9 P1	771.1102^{+4}	**a_{23}-$H_3PO_4^{+4}$**	C125 H204 N39 O48 P2	1429.5913	**IR(ADP-Ribosyl)DLLTPP-H_2O**	C57 H91 N16 O23 P2
195.1128	**PP**	C10 H15 N2 O2	774.3182^{+2}	**a_9-H_2O^{+2}**	C60 H97 N18 O26 P2	1430.5753	**IR(ADP-Ribosyl)DLLTPP-NH_3**	C57 H90 N15 O24 P2
197.1285	**LT-H_2O**	C10 H17 N2 O2	774.3322^{+4}	**b_{22}^{+4}**	C120 H196 N38 O52 P3	1434.6211^{+2}	**b_{21}-$H_3PO_4^{+2}$**	C114 H181 N36 O47 P2
198.1237	**PQ-CO**	C9 H16 N3 O2	774.8102^{+2}	**a_9-NH_3^{+2}**	C60 H96 N17 O27 P2	1435.5655	**R(ADP-Ribosyl)DLLTPPT**	C55 H89 N16 O25 P2
198.1601	**KP-CO**	C10 H20 N3 O1	776.3451	**DNRS(Phospho)KL-H_2O**	C29 H51 N11 O12 P1	1446.5814	**a_8-H_2O**	C56 H90 N17 O24 P2
199.0713	**TD-H_2O**	C8 H11 N2 O4	776.3937	**TPPTDKPG-H_2O**	C35 H54 N9 O11	1446.6373	**TPPTDKPGQDNRS(Phospho)-CO**	C56 H93 N19 O24 P1

199.1077	TP	C9 H15 N2 O3	777.3291	DNRS(Phospho)KL-NH_3	C29 H50 N10 O13 P1	1447.5655	a_8-NH_3	C56 H89 N16 O25 P2
199.1077	PT	C9 H15 N2 O3	777.3777	TPPTDKPG-NH_3	C35 H53 N8 O12	1447.6018	IR(ADP-Ribosyl)DLLTPP	C57 H93 N16 O24 P2
199.1805	LL-CO	C11 H23 N2 O1	778.1090^{+4}	b_{23}-$H_3PO_4^{+4}$	C126 H204 N39 O49 P2	1450.6127	QIR(ADP-Ribosyl)DLLTP-CO	C56 H94 N17 O24 P2
200.1394^{+2}	y_3-NH_3^{+2}	C18 H35 N6 O4	778.3955	KPGQDNR-H_2O	C32 H52 N13 O10	1450.6127	PQIR(ADP-Ribosyl)DLLT-CO	C56 H94 N17 O24 P2
201.1234	DL-CO	C9 H17 N2 O3	778.8348^{+4}	b_{22}+H_2O^{+4}	C120 H198 N38 O53 P3	1455.6385^{+2}	y_{20}-H_2O^{+2}	C112 H188 N38 O47 P3
202.0822	DN-CO	C7 H12 N3 O4	779.3795	KPGQDNR-NH_3	C32 H51 N12 O11	1456.1305^{+2}	y_{20}-NH_3^{+2}	C112 H187 N37 O48 P3
208.6526^{+2}	y_3^{+2}	C18 H38 N7 O4	781.3352	QDNRS(Phospho)K-CO	C27 H50 N12 O13 P1	1456.6216	TPPTDKPGQDNRS(Phospho)-H_2O	C57 H91 N19 O24 P1
209.0921	PQ-NH_3	C10 H13 N2 O3	783.2586	IR(ADP-Ribosyl)-CO	C26 H45 N10 O14 P2	1457.6057	TPPTDKPGQDNRS(Phospho)-NH_3	C57 H90 N18 O25 P1
209.1285	KP-NH_3	C11 H17 N2 O2	783.3235^{+2}	a_9^{+2}	C60 H99 N18 O27 P2	1460.5971	PQIR(ADP-Ribosyl)DLLT-H_2O	C57 H92 N17 O24 P2
209.8589^{+4}	y_6-NH_3^{+4}	C31 H59 N13 O12 P1	785.2015	R(ADP-Ribosyl)D-CO	C24 H39 N10 O16 P2	1460.5971	QIR(ADP-Ribosyl)DLLTP-H_2O	C57 H92 N17 O24 P2
211.1077	DL-H_2O	C10 H15 N2 O3	788.3157^{+2}	b_9-H_2O^{+2}	C61 H97 N18 O27 P2	1460.6068^{+2}	a_{21}-H_2O^{+2}	C113 H182 N36 O49 P3
212.0666	DN-H_2O	C8 H10 N3 O4	788.6085^{+4}	y_{22}-H_2O^{+4}	C123 H207 N41 O50 P3	1461.0988^{+2}	a_{21}-NH_3^{+2}	C113 H181 N35 O50 P3
213.0506	DN-NH_3	C8 H9 N2 O5	788.8077^{+2}	b_9-NH_3^{+2}	C61 H96 N17 O28 P2	1461.5811	PQIR(ADP-Ribosyl)DLLT-NH_3	C57 H91 N16 O25 P2
213.0870	b_2	C9 H13 N2 O4	788.8545^{+4}	y_{22}-NH_3^{+4}	C123 H206 N40 O51 P3	1461.5811	QIR(ADP-Ribosyl)DLLTP-NH_3	C57 H91 N16 O25 P2
214.1156^{+4}	y_6^{+4}	C31 H62 N14 O12 P1	788.8832^{+2}	y_{13}-H_2O^{+2}	C61 H107 N23 O24 P1	1464.5920	a_8	C56 H92 N17 O25 P2
214.1550	QI-CO	C10 H20 N3 O2	789.3752^{+2}	y_{13}-NH_3^{+2}	C61 H106 N22 O25 P1	1464.6437^{+2}	y_{20}^{+2}	C112 H190 N38 O48 P3
214.1914	KL-CO	C11 H24 N3 O1	790.0172^{+3}	a_{17}-H_2O^{+3}	C96 H153 N28 O38 P2	1464.7329	DLLTPPTDKPGQDN-CO	C63 H102 N17 O23
214.4784^{+3}	y_5-$H_3PO_4^{+3}$	C27 H53 N12 O6	790.3452^{+3}	a_{17}-NH_3^{+3}	C96 H152 N27 O39 P2	1469.6121^{+2}	a_{21}^{+2}	C113 H184 N36 O50 P3
215.1390	LT	C10 H19 N2 O3	791.1018^{+4}	a_{23}-H_2O^{+4}	C125 H205 N39 O51 P3	1472.1973^{+2}	y_{21}-$H_3PO_4^{+2}$	C118 H198 N39 O45 P2
216.0979	QD-CO	C8 H14 N3 O4	791.3196	QDNRS(Phospho)K-H_2O	C28 H48 N12 O13 P1	1473.6846	PPTDKPGQDNRS(Phospho)K-CO	C58 H98 N20 O23 P1
216.1343	DK-CO	C9 H18 N3 O3	791.3478^{+4}	a_{23}-NH_3^{+4}	C125 H204 N38 O52 P3	1474.5764	b_8-H_2O	C57 H90 N17 O25 P2
217.0819	TD	C8 H13 N2 O5	792.3036	QDNRS(Phospho)K-NH_3	C28 H47 N11 O14 P1	1474.6043^{+2}	b_{21}-H_2O^{+2}	C114 H182 N36 O50 P3
218.3781^{+4}	y_7-$H_3PO_4^{+4}$	C35 H64 N15 O11	792.8801^{+4}	y_{23}-$H_3PO_4^{+4}$	C128 H213 N42 O48 P2	1474.6322	TPPTDKPGQDNRS(Phospho)	C57 H93 N19 O25 P1
225.1234	QI-NH_3	C11 H17 N2 O3	793.1112^{+4}	y_{22}^{+4}	C123 H209 N41 O51 P3	1474.7172	DLLTPPTDKPGQDN-H_2O	C64 H100 N17 O23
225.1598	KL-NH_3	C12 H21 N2 O2	793.4203	PPTDKPGQ-CO	C35 H57 N10 O11	1475.0963^{+2}	b_{21}-NH_3^{+2}	C114 H181 N35 O51 P3
226.0822	QD-H_2O	C9 H12 N3 O4	794.2270	IR(ADP-Ribosyl)-NH_3	C27 H42 N9 O15 P2	1475.5604	b_8-NH_3	C57 H89 N16 O26 P2
226.1186	DK-H_2O	C10 H16 N3 O3	794.3556	DNRS(Phospho)KL	C29 H53 N11 O13 P1	1475.7013	DLLTPPTDKPGQDN-NH_3	C64 H99 N16 O24
226.1186	PQ	C10 H16 N3 O3	794.4043	TPPTDKPG	C35 H56 N9 O12	1475.7115	y_{12}-H_2O	C57 H100 N22 O22 P1
226.1550	KP	C11 H20 N3 O2	795.1859	R(ADP-Ribosyl)D-H_2O	C25 H37 N10 O16 P2	1476.6955	y_{12}-NH_3	C57 H99 N21 O23 P1
227.0662	QD-NH_3	C9 H11 N2 O5	795.6044^{+4}	a_{23}^{+4}	C125 H207 N39 O52 P3	1478.6077	PQIR(ADP-Ribosyl)DLLT	C57 H94 N17 O25 P2
227.1026	DK-NH_3	C10 H15 N2 O4	796.0207^{+3}	a_{17}^{+3}	C96 H155 N28 O39 P2	1478.6077	QIR(ADP-Ribosyl)DLLTP	C57 H94 N17 O25 P2
227.1754	LL	C12 H23 N2 O2	796.1699	R(ADP-Ribosyl)D-NH_3	C25 H36 N9 O17 P2	1483.6096^{+2}	b_{21}^{+2}	C114 H184 N36 O51 P3
229.1183	DL	C10 H17 N2 O4	796.4060	KPGQDNR	C32 H54 N13 O11	1483.6689	PPTDKPGQDNRS(Phospho)K-H_2O	C59 H96 N20 O23 P1
230.0771	DN	C8 H12 N3 O5	797.3209^{+2}	b_9^{+2}	C61 H99 N18 O28 P2	1484.6529	PPTDKPGQDNRS(Phospho)K-NH_3	C59 H95 N19 O24 P1
238.3696^{+4}	y_7-H_2O^{+4}	C35 H65 N15 O14 P1	797.4264^{+2}	y_{14}-$H_3PO_4^{+2}$	C66 H113 N24 O22	1484.6711^{+2}	a_{22}-$H_3PO_4^{+2}$	C119 H193 N38 O47 P2
238.6156^{+4}	y_7-NH_3^{+4}	C35 H64 N14 O15 P1	797.8885^{+2}	y_{13}^{+2}	C61 H109 N23 O25 P1	1489.7159	PTDKPGQDNRS(Phospho)KL-CO	C59 H102 N20 O23 P1
241.4618^{+3}	y_5-NH_3^{+3}	C27 H53 N11 O10 P1	798.1005^{+4}	b_{23}-H_2O^{+4}	C126 H205 N39 O52 P3	1492.5869	b_8	C57 H92 N17 O26 P2
242.1499	QI	C11 H20 N3 O3	798.3465^{+4}	b_{23}-NH_3^{+4}	C126 H204 N38 O53 P3	1492.7278	DLLTPPTDKPGQDN	C64 H102 N17 O24
242.1863	KL	C12 H24 N3 O2	799.3488^{+3}	b_{17}-H_2O^{+3}	C97 H153 N28 O39 P2	1493.7220	y_{12}	C57 H102 N22 O23 P1
242.8723^{+4}	y_7^{+4}	C35 H67 N15 O15 P1	799.6768^{+3}	b_{17}-NH_3^{+3}	C97 H152 N27 O40 P2	1496.7928	y_{13}-H_3PO_4	C61 H106 N23 O21
243.1564	NR-CO	C9 H19 N6 O2	802.6032^{+4}	b_{23}^{+4}	C126 H207 N39 O53 P3	1498.6686^{+2}	b_{22}-$H_3PO_4^{+2}$	C120 H193 N38 O48 P2
243.1634^{+2}	y_4-$H_3PO_4^{+2}$	C21 H41 N8 O5	803.4046	PPTDKPGQ-H_2O	C36 H55 N10 O11	1499.7002	PTDKPGQDNRS(Phospho)KL-H_2O	C60 H100 N20 O23 P1
244.0928	QD	C9 H14 N3 O5	804.3886	PPTDKPGQ-NH_3	C36 H54 N9 O12	1500.6842	PTDKPGQDNRS(Phospho)KL-NH_3	C60 H99 N19 O24 P1

244.1292	**DK**	C10 H18 N3 O4	805.3524^{+3}	**b_{17}^{+3}**	C97 H155 N28 O40 P2	1501.6795	**PPTDKPGQDNRS(Phospho)K**	C59 H98 N20 O24 P1
247.1373^{+3}	**y_5^{+3}**	C27 H56 N12 O10 P1	807.1058^{+4}	**$b_{23}+H_2O^{+4}$**	C126 H209 N39 O54 P3	1505.8071	**LLTPPTDKPGQDNR-CO**	C65 H109 N20 O21
250.3927^{+4}	**$y_8-H_3PO_4^{+4}$**	C40 H72 N17 O13	807.3145	**PGQDNRS(Phospho)-CO**	C28 H48 N12 O14 P1	1512.1805^{+2}	**$y_{21}-H_2O^{+2}$**	C118 H199 N39 O48 P3
252.4927^{+3}	**$y_6-H_3PO_4^{+3}$**	C31 H59 N14 O8	809.3302	**QDNRS(Phospho)K**	C28 H50 N12 O14 P1	1512.6725^{+2}	**$y_{21}-NH_3^{+2}$**	C118 H198 N38 O49 P3
254.1248	**NR-NH_3**	C10 H16 N5 O3	811.2536	**IR(ADP-Ribosyl)**	C27 H45 N10 O15 P2	1515.7914	**LLTPPTDKPGQDNR-H_2O**	C66 H107 N20 O21
255.1452	**PGQ-CO**	C11 H19 N4 O3	811.3945	**PTDKPGQD-CO**	C34 H55 N10 O13	1516.7754	**LLTPPTDKPGQDNR-NH_3**	C66 H106 N19 O22
255.1816	**KPG-CO**	C12 H23 N4 O2	812.8717^{+4}	**$y_{23}-H_2O^{+4}$**	C128 H214 N42 O51 P3	1517.7108	**PTDKPGQDNRS(Phospho)KL**	C60 H102 N20 O24 P1
264.6481^{+4}	**$y_9-H_3PO_4^{+4}$**	C42 H75 N18 O14	813.1177^{+4}	**$y_{23}-NH_3^{+4}$**	C128 H213 N41 O52 P3	1520.6546	**IR(ADP-Ribosyl)DLLTPPT-CO**	C60 H100 N17 O25 P2
266.1135	**PGQ-NH_3**	C12 H16 N3 O4	813.1964	**R(ADP-Ribosyl)D**	C25 H39 N10 O17 P2	1521.1858^{+2}	**y_{21}^{+2}**	C118 H201 N39 O49 P3
266.1499	**KPG-NH_3**	C13 H20 N3 O3	817.2989	**PGQDNRS(Phospho)-H_2O**	C29 H46 N12 O14 P1	1522.5975	**R(ADP-Ribosyl)DLLTPPTD-CO**	C58 H94 N17 O27 P2
268.1057	**S(Phospho)K-CO**	C8 H19 N3 O5 P1	817.3744^{+4}	**y_{23}^{+4}**	C128 H216 N42 O52 P3	1524.6543^{+2}	**$a_{22}-H_2O^{+2}$**	C119 H194 N38 O50 P3
268.1656	**PPT-CO**	C13 H22 N3 O3	818.2829	**PGQDNRS(Phospho)-NH_3**	C29 H45 N11 O15 P1	1525.1463^{+2}	**$a_{22}-NH_3^{+2}$**	C119 H193 N37 O51 P3
268.1656	**TPP-CO**	C13 H22 N3 O3	821.3788	**PTDKPGQD-H_2O**	C35 H53 N10 O13	1530.6390	**IR(ADP-Ribosyl)DLLTPPT-H_2O**	C61 H98 N17 O25 P2
270.3843^{+4}	**$y_8-H_2O^{+4}$**	C40 H73 N17 O16 P1	821.4152	**PPTDKPGQ**	C36 H57 N10 O12	1531.6230	**IR(ADP-Ribosyl)DLLTPPT-NH_3**	C61 H97 N16 O26 P2
270.6303^{+4}	**$y_8-NH_3^{+4}$**	C40 H72 N16 O17 P1	821.6369^{+4}	**$MH-H_3PO_4^{+4}$**	C132 H218 N43 O51 P2	1532.5818	**R(ADP-Ribosyl)DLLTPPTD-H_2O**	C59 H92 N17 O27 P2
271.1513	**NR**	C10 H19 N6 O3	822.3628	**PTDKPGQD-NH_3**	C35 H52 N9 O14	1533.5658	**R(ADP-Ribosyl)DLLTPPTD-NH_3**	C59 H91 N16 O28 P2
271.1765	**y_2-NH_3**	C12 H23 N4 O3	822.4720	**LTPPTDKP-CO**	C38 H64 N9 O11	1533.6596^{+2}	**a_{22}^{+2}**	C119 H196 N38 O51 P3
273.1193	**GQD-CO**	C10 H17 N4 O5	822.8446^{+2}	**$a_{10}-H_2O^{+2}$**	C65 H104 N19 O27 P2	1533.8020	**LLTPPTDKPGQDNR**	C66 H109 N20 O22
274.8869^{+4}	**y_8^{+4}**	C40 H75 N17 O17 P1	823.3366^{+2}	**$a_{10}-NH_3^{+2}$**	C65 H103 N18 O28 P2	1536.2266^{+2}	**$y_{22}-H_3PO_4^{+2}$**	C123 H206 N41 O47 P2
278.0900	**S(Phospho)K-H_2O**	C9 H17 N3 O5 P1	825.4353	**DLLTPPTD-CO**	C37 H61 N8 O13	1538.6518^{+2}	**$b_{22}-H_2O^{+2}$**	C120 H194 N38 O51 P3
278.1499	**TPP-H_2O**	C14 H20 N3 O3	828.3595^{+3}	**$a_{18}-H_2O^{+3}$**	C100 H158 N29 O41 P2	1539.1438^{+2}	**$b_{22}-NH_3^{+2}$**	C120 H193 N37 O52 P3
278.1499	**PPT-H_2O**	C14 H20 N3 O3	828.3846	**TDKPGQDN-CO**	C33 H54 N11 O14	1541.2132^{+2}	**$a_{23}-H_3PO_4^{+2}$**	C125 H204 N39 O48 P2
279.0740	**S(Phospho)K-NH_3**	C9 H16 N2 O6 P1	828.6875^{+3}	**$a_{18}-NH_3^{+3}$**	C100 H157 N28 O42 P2	1547.6291	**a_9-H_2O**	C60 H97 N18 O26 P2
279.4761^{+3}	**$y_6-NH_3^{+3}$**	C31 H59 N13 O12 P1	831.8499^{+2}	**a_{10}^{+2}**	C65 H106 N19 O28 P2	1547.6570^{+2}	**b_{22}^{+2}**	C120 H196 N38 O52 P3
283.1037	**GQD-H_2O**	C11 H15 N4 O5	832.4563	**LTPPTDKP-H_2O**	C39 H62 N9 O11	1547.6655	**QIR(ADP-Ribosyl)DLLTPP-CO**	C61 H101 N18 O25 P2
283.1401	**PGQ**	C12 H19 N4 O4	833.4403	**LTPPTDKP-NH_3**	C39 H61 N8 O12	1547.6655	**PQIR(ADP-Ribosyl)DLLTP-CO**	C61 H101 N18 O25 P2
283.1765	**KPG**	C13 H23 N4 O3	834.3630^{+3}	**a_{18}^{+3}**	C100 H160 N29 O42 P2	1548.6131	**a_9-NH_3**	C60 H96 N17 O27 P2
283.6385^{+2}	**$y_4-NH_3^{+2}$**	C21 H41 N7 O9 P1	835.3094	**PGQDNRS(Phospho)**	C29 H48 N12 O15 P1	1548.6495	**IR(ADP-Ribosyl)DLLTPPT**	C61 H100 N17 O26 P2
284.0877	**GQD-NH_3**	C11 H14 N3 O6	835.4196	**DLLTPPTD-H_2O**	C38 H59 N8 O13	1550.5924	**R(ADP-Ribosyl)DLLTPPTD**	C59 H94 N17 O28 P2
284.1969	**LTP-CO**	C14 H26 N3 O3	836.4138	**y_6-NH_3**	C31 H59 N13 O12 P1	1555.2106^{+2}	**$b_{23}-H_3PO_4^{+2}$**	C126 H204 N39 O49 P2
284.6397^{+4}	**$y_9-H_2O^{+4}$**	C42 H76 N18 O17 P1	836.8420^{+2}	**$b_{10}-H_2O^{+2}$**	C66 H104 N19 O28 P2	1556.6623^{+2}	**$b_{22}+H_2O^{+2}$**	C120 H198 N38 O53 P3
284.8857^{+4}	**$y_9-NH_3^{+4}$**	C42 H75 N17 O18 P1	837.3340^{+2}	**$b_{10}-NH_3^{+2}$**	C66 H103 N18 O29 P2	1557.6499	**QIR(ADP-Ribosyl)DLLTPP-H_2O**	C62 H99 N18 O25 P2
285.1516^{+3}	**y_6^{+3}**	C31 H62 N14 O12 P1	837.4096^{+2}	**$y_{14}-H_2O^{+2}$**	C66 H114 N24 O25 P1	1557.6499	**PQIR(ADP-Ribosyl)DLLTP-H_2O**	C62 H99 N18 O25 P2
286.1397	**PTD-CO**	C12 H20 N3 O5	837.6911^{+3}	**$b_{18}-H_2O^{+3}$**	C101 H158 N29 O42 P2	1558.6339	**QIR(ADP-Ribosyl)DLLTPP-NH_3**	C62 H98 N17 O26 P2
288.2030	**y_2**	C12 H26 N5 O3	837.9016^{+2}	**$y_{14}-NH_3^{+2}$**	C66 H113 N23 O26 P1	1558.6339	**PQIR(ADP-Ribosyl)DLLTP-NH_3**	C62 H98 N17 O26 P2
288.9113^{+4}	**$y_{10}-H_3PO_4^{+4}$**	C47 H82 N19 O15	838.0192^{+3}	**$b_{18}-NH_3^{+3}$**	C101 H157 N28 O43 P2	1559.7214	**LTPPTDKPGQDNRS(Phospho)-CO**	C62 H104 N20 O25 P1
289.1423^{+4}	**y_9^{+4}**	C42 H78 N18 O18 P1	838.3567	**GQDNRS(Phospho)K-CO**	C29 H53 N13 O14 P1	1565.6397	**a_9**	C60 H99 N18 O27 P2
290.8350^{+3}	**$y_7-H_3PO_4^{+3}$**	C35 H64 N15 O11	838.3690	**TDKPGQDN-H_2O**	C34 H52 N11 O14	1569.7057	**LTPPTDKPGQDNRS(Phospho)-H_2O**	C63 H102 N20 O25 P1
292.1518^{+2}	**y_4^{+2}**	C21 H44 N8 O9 P1	838.5033	**LLTPPTDK-CO**	C39 H68 N9 O11	1570.6897	**LTPPTDKPGQDNRS(Phospho)-NH_3**	C63 H101 N19 O26 P1
294.1812	**LTP-H_2O**	C15 H24 N3 O3	839.3530	**TDKPGQDN-NH_3**	C34 H51 N10 O15	1574.7323	**TPPTDKPGQDNRS(Phospho)K-CO**	C62 H105 N21 O25 P1
295.1401	**a_3-H_2O**	C13 H19 N4 O4	839.3894	**PTDKPGQD**	C35 H55 N10 O14	1575.6240	**b_9-H_2O**	C61 H97 N18 O27 P2
296.1006	**S(Phospho)K**	C9 H19 N3 O6 P1	841.6285^{+4}	**$MH-H_2O^{+4}$**	C132 H219 N43 O54 P3	1575.6604	**QIR(ADP-Ribosyl)DLLTPP**	C62 H101 N18 O26 P2
296.1118	**RS(Phospho)-CO**	C8 H19 N5 O5 P1	841.8745^{+4}	**$MH-NH_3^{+4}$**	C132 H218 N42 O55 P3	1575.6604	**PQIR(ADP-Ribosyl)DLLTP**	C62 H101 N18 O26 P2

296.1241	**PTD-H_2O**	C13 H18 N3 O5	843.6947^{+3}	**b_{18}^{+3}**	C101 H160 N29 O43 P2	1576.2098^{+2}	**$y_{22}-H_2O^{+2}$**	C123 H207 N41 O50 P3
296.1241	**a_3-NH_3**	C13 H18 N3 O5	845.8473^{+2}	**b_{10}^{+2}**	C66 H106 N19 O29 P2	1576.6080	**b_9-NH_3**	C61 H96 N17 O28 P2
296.1605	**PPT**	C14 H22 N3 O4	845.9528^{+2}	**$y_{15}-H_3PO_4^{+2}$**	C71 H120 N25 O23	1576.7018^{+2}	**$y_{22}-NH_3^{+2}$**	C123 H206 N40 O51 P3
296.1605	**TPP**	C14 H22 N3 O4	846.1311^{+4}	**MH^{+4}**	C132 H221 N43 O55 P3	1576.7591	**$y_{13}-H_2O$**	C61 H107 N23 O24 P1
300.2282	**LLT-CO**	C15 H30 N3 O3	846.4149^{+2}	**y_{14}^{+2}**	C66 H116 N24 O26 P1	1577.7432	**$y_{13}-NH_3$**	C61 H106 N22 O25 P1
301.1143	**GQD**	C11 H17 N4 O6	848.3411	**GQDNRS(Phospho)K-H_2O**	C30 H51 N13 O14 P1	1581.1963^{+2}	**$a_{23}-H_2O^{+2}$**	C125 H205 N39 O51 P3
306.0962	**RS(Phospho)-H_2O**	C9 H17 N5 O5 P1	848.4876	**LLTPPTDK-H_2O**	C40 H66 N9 O11	1581.6883^{+2}	**$a_{23}-NH_3^{+2}$**	C125 H204 N38 O52 P3
307.0802	**RS(Phospho)-NH_3**	C9 H16 N4 O6 P1	849.3251	**GQDNRS(Phospho)K-NH_3**	C30 H50 N12 O15 P1	1584.7166	**TPPTDKPGQDNRS(Phospho)K-H_2O**	C63 H103 N21 O25 P1
308.9028^{+4}	**$y_{10}-H_2O^{+4}$**	C47 H83 N19 O18 P1	849.4716	**LLTPPTDK-NH_3**	C40 H65 N8 O12	1584.7530^{+2}	**$y_{23}-H_3PO_4^{+2}$**	C128 H213 N42 O48 P2
309.1489^{+4}	**$y_{10}-NH_3^{+4}$**	C47 H82 N18 O19 P1	850.4669	**LTPPTDKP**	C39 H64 N9 O12	1585.2151^{+2}	**y_{22}^{+2}**	C123 H209 N41 O51 P3
310.2125	**LLT-H_2O**	C16 H28 N3 O3	853.4302	**DLLTPPTD**	C38 H61 N8 O14	1585.7006	**TPPTDKPGQDNRS(Phospho)K-NH_3**	C63 H102 N20 O26 P1
311.2078	**PQI-CO**	C15 H27 N4 O3	853.4404	**y_6**	C31 H62 N14 O12 P1	1586.7686	**PPTDKPGQDNRS(Phospho)KL-CO**	C64 H109 N21 O24 P1
312.1918	**LTP**	C15 H26 N3 O4	856.3795	**TDKPGQDN**	C34 H54 N11 O15	1587.7163	**LTPPTDKPGQDNRS(Phospho)**	C63 H104 N20 O26 P1
313.1506	**a_3**	C13 H21 N4 O5	866.3516	**GQDNRS(Phospho)K**	C30 H53 N13 O15 P1	1590.2016^{+2}	**a_{23}^{+2}**	C125 H207 N39 O52 P3
313.1870	**DKP-CO**	C14 H25 N4 O4	866.3738^{+3}	**$a_{19}-H_2O^{+3}$**	C104 H164 N31 O43 P2	1593.6346	**b_9**	C61 H99 N18 O28 P2
313.4055^{+4}	**y_{10}^{+4}**	C47 H85 N19 O19 P1	866.4982	**LLTPPTDK**	C40 H68 N9 O12	1593.8456	**$y_{14}-H_3PO_4$**	C66 H113 N24 O22
314.1347	**PTD**	C13 H20 N3 O6	866.7018^{+3}	**$a_{19}-NH_3^{+3}$**	C104 H163 N30 O44 P2	1594.7697	**y_{13}**	C61 H109 N23 O25 P1
314.2074	**DLL-CO**	C15 H28 N3 O4	870.4904	**$y_7-H_3PO_4$**	C35 H64 N15 O11	1595.1938^{+2}	**$b_{23}-H_2O^{+2}$**	C126 H205 N39 O52 P3
317.1819	**TDK-CO**	C13 H25 N4 O5	871.3710^{+2}	**$a_{11}-H_2O^{+2}$**	C70 H111 N20 O28 P2	1595.6858^{+2}	**$b_{23}-NH_3^{+2}$**	C126 H204 N38 O53 P3
317.4904^{+3}	**$y_7-H_2O^{+3}$**	C35 H65 N15 O14 P1	871.8630^{+2}	**$a_{11}-NH_3^{+2}$**	C70 H110 N19 O29 P2	1596.7530	**PPTDKPGQDNRS(Phospho)KL-H_2O**	C65 H107 N21 O24 P1
317.8184^{+3}	**$y_7-NH_3^{+3}$**	C35 H64 N14 O15 P1	872.3773^{+3}	**a_{19}^{+3}**	C104 H166 N31 O44 P2	1597.7370	**PPTDKPGQDNRS(Phospho)KL-NH_3**	C65 H106 N20 O25 P1
320.9350^{+4}	**$y_{11}-H_3PO_4^{+4}$**	C53 H94 N21 O16	875.7055^{+3}	**$b_{19}-H_2O^{+3}$**	C105 H164 N31 O44 P2	1602.7272	**TPPTDKPGQDNRS(Phospho)K**	C63 H105 N21 O26 P1
321.2139^{+2}	**$y_5-H_3PO_4^{+2}$**	C27 H53 N12 O6	876.0335^{+3}	**$b_{19}-NH_3^{+3}$**	C105 H163 N30 O45 P2	1604.1991^{+2}	**b_{23}^{+2}**	C126 H207 N39 O53 P3
322.1761	**PQI-NH_3**	C16 H24 N3 O4	879.4934	**LTPPTDKPG-CO**	C40 H67 N10 O12	1613.2044^{+2}	**$b_{23}+H_2O^{+2}$**	C126 H209 N39 O54 P3
323.1350	**b_3-H_2O**	C14 H19 N4 O5	880.3762^{+2}	**a_{11}^{+2}**	C70 H113 N20 O29 P2	1614.7636	**PPTDKPGQDNRS(Phospho)KL**	C65 H109 N21 O25 P1
323.1714	**DKP-H_2O**	C15 H23 N4 O4	881.7090^{+3}	**b_{19}^{+3}**	C105 H166 N31 O45 P2	1620.8340	**DLLTPPTDKPGQDNR-CO**	C69 H114 N21 O24
323.4940^{+3}	**y_7^{+3}**	C35 H67 N15 O15 P1	883.4381	**DKPGQDNR-CO**	C35 H59 N14 O13	1624.7362^{+2}	**$y_{23}-H_2O^{+2}$**	C128 H214 N42 O51 P3
324.1067	**RS(Phospho)**	C9 H19 N5 O6 P1	885.3684^{+2}	**$b_{11}-H_2O^{+2}$**	C71 H111 N20 O29 P2	1625.2282^{+2}	**$y_{23}-NH_3^{+2}$**	C128 H213 N41 O52 P3
324.1190	**b_3-NH_3**	C14 H18 N3 O6	885.8604^{+2}	**$b_{11}-NH_3^{+2}$**	C71 H110 N19 O30 P2	1630.8184	**DLLTPPTDKPGQDNR-H_2O**	C70 H112 N21 O24
324.1554	**DKP-NH_3**	C15 H22 N3 O5	885.9360^{+2}	**$y_{15}-H_2O^{+2}$**	C71 H121 N25 O26 P1	1631.8024	**DLLTPPTDKPGQDNR-NH_3**	C70 H111 N20 O25
324.1918	**DLL-H_2O**	C16 H26 N3 O4	886.4280^{+2}	**$y_{15}-NH_3^{+2}$**	C71 H120 N24 O27 P1	1633.7414^{+2}	**y_{23}^{+2}**	C128 H216 N42 O52 P3
327.1663	**TDK-H_2O**	C14 H23 N4 O5	889.4778	**LTPPTDKPG-H_2O**	C41 H65 N10 O12	1635.6815	**IR(ADP-Ribosyl)DLLTPPTD-CO**	C64 H105 N18 O28 P2
328.1503	**TDK-NH_3**	C14 H22 N3 O6	890.4618	**LTPPTDKPG-NH_3**	C41 H64 N9 O13	1642.2665^{+2}	**$MH-H_3PO_4^{+2}$**	C132 H218 N43 O51 P2
328.2231	**LLT**	C16 H30 N3 O4	893.4224	**DKPGQDNR-H_2O**	C36 H57 N14 O13	1644.6819	**$a_{10}-H_2O$**	C65 H104 N19 O27 P2
330.1408	**QDN-CO**	C12 H20 N5 O6	894.3737^{+2}	**b_{11}^{+2}**	C71 H113 N20 O30 P2	1644.7183	**PQIR(ADP-Ribosyl)DLLTPP-CO**	C66 H108 N19 O26 P2
333.5212^{+3}	**$y_8-H_3PO_4^{+3}$**	C40 H72 N17 O13	894.4064	**DKPGQDNR-NH_3**	C36 H56 N13 O14	1645.6659	**IR(ADP-Ribosyl)DLLTPPTD-H_2O**	C65 H103 N18 O28 P2
339.2027	**PQI**	C16 H27 N4 O4	894.4193	**QDNRS(Phospho)KL-CO**	C33 H61 N13 O14 P1	1645.6659	**$a_{10}-NH_3$**	C65 H103 N18 O28 P2
340.1252	**QDN-H_2O**	C13 H18 N5 O6	894.4680	**TPPTDKPGQ-CO**	C39 H64 N11 O13	1646.6499	**IR(ADP-Ribosyl)DLLTPPTD-NH_3**	C65 H102 N17 O29 P2
340.9266^{+4}	**$y_{11}-H_2O^{+4}$**	C53 H95 N21 O19 P1	894.9413^{+2}	**y_{15}^{+2}**	C71 H123 N25 O27 P1	1648.7132	**QIR(ADP-Ribosyl)DLLTPPT-CO**	C65 H108 N19 O27 P2
341.1092	**QDN-NH_3**	C13 H17 N4 O7	896.4766^{+2}	**$y_{16}-H_3PO_4^{+2}$**	C75 H127 N26 O25	1648.8289	**DLLTPPTDKPGQDNR**	C70 H114 N21 O25
341.1456	**b_3**	C14 H21 N4 O6	898.2856	**R(ADP-Ribosyl)DL-CO**	C30 H50 N11 O17 P2	1650.6924	**R(ADP-Ribosyl)DLLTPPTDK-CO**	C64 H106 N19 O28 P2
341.1726^{+4}	**$y_{11}-NH_3^{+4}$**	C53 H94 N20 O20 P1	898.2856	**IR(ADP-Ribosyl)D-CO**	C30 H50 N11 O17 P2	1654.7026	**PQIR(ADP-Ribosyl)DLLTPP-H_2O**	C67 H106 N19 O26 P2
341.1819	**DKP**	C15 H25 N4 O5	904.4037	**QDNRS(Phospho)KL-H_2O**	C34 H59 N13 O14 P1	1655.6866	**PQIR(ADP-Ribosyl)DLLTPP-NH_3**	C67 H105 N18 O27 P2

342.2023	DLL	C16 H28 N3 O5	904.4523	TPPTDKPGQ-H_2O	C40 H62 N11 O13	1658.6975	QIR(ADP-Ribosyl)DLLTPPT-H_2O	C66 H106 N19 O27 P2
345.1769	TDK	C14 H25 N4 O6	905.3877	QDNRS(Phospho)KL-NH_3	C34 H58 N12 O15 P1	1659.6815	QIR(ADP-Ribosyl)DLLTPPT-NH_3	C66 H105 N18 O28 P2
345.4292^{+4}	y_{11}^{+4}	C53 H97 N21 O20 P1	905.4363	TPPTDKPGQ-NH_3	C40 H61 N10 O14	1660.6768	R(ADP-Ribosyl)DLLTPPTDK-H_2O	C65 H104 N19 O28 P2
349.6917^{+4}	y_{12}-$H_3PO_4^{+4}$	C57 H99 N22 O19	907.4884	LTPPTDKPG	C41 H67 N10 O13	1661.6608	R(ADP-Ribosyl)DLLTPPTDK-NH_3	C65 H103 N18 O29 P2
352.5283^{+3}	y_9-$H_3PO_4^{+3}$	C42 H75 N18 O14	908.2699	R(ADP-Ribosyl)DL-H_2O	C31 H48 N11 O17 P2	1662.6924	a_{10}	C65 H106 N19 O28 P2
358.1357	QDN	C13 H20 N5 O7	908.2699	IR(ADP-Ribosyl)D-H_2O	C31 H48 N11 O17 P2	1663.6765	IR(ADP-Ribosyl)DLLTPPTD	C65 H105 N18 O29 P2
358.1833	DNR-CO	C13 H24 N7 O5	908.4472	PPTDKPGQD-CO	C39 H62 N11 O14	1672.6768	b_{10}-H_2O	C66 H104 N19 O28 P2
360.1766^{+3}	y_8-H_2O^{+3}	C40 H73 N17 O16 P1	909.2540	R(ADP-Ribosyl)DL-NH_3	C31 H47 N10 O18 P2	1672.7132	PQIR(ADP-Ribosyl)DLLTPP	C67 H108 N19 O27 P2
360.5046^{+3}	y_8-NH_3^{+3}	C40 H72 N16 O17 P1	909.2540	IR(ADP-Ribosyl)D-NH_3	C31 H47 N10 O18 P2	1672.8054	LLTPPTDKPGQDNRS(Phospho)-CO	C68 H115 N21 O26 P1
361.6891^{+2}	y_5-NH_3^{+2}	C27 H53 N11 O10 P1	911.3172	QIR(ADP-Ribosyl)-CO	C31 H53 N12 O16 P2	1673.6608	b_{10}-NH_3	C66 H103 N18 O29 P2
366.1801^{+3}	y_8^{+3}	C40 H75 N17 O17 P1	911.4330	DKPGQDNR	C36 H59 N14 O14	1673.8119	y_{14}-H_2O	C66 H114 N24 O25 P1
368.1677	DNR-H_2O	C14 H22 N7 O5	918.4075^{+3}	a_{20}-H_2O^{+3}	C110 H176 N35 O44 P2	1674.7959	y_{14}-NH_3	C66 H113 N23 O26 P1
369.1517	DNR-NH_3	C14 H21 N6 O6	918.4316	PPTDKPGQD-H_2O	C40 H60 N11 O14	1676.7081	QIR(ADP-Ribosyl)DLLTPPT	C66 H108 N19 O28 P2
369.2132	TPPT-CO	C17 H29 N4 O5	918.7355^{+3}	a_{20}-NH_3^{+3}	C110 H175 N34 O45 P2	1678.6874	R(ADP-Ribosyl)DLLTPPTDK	C65 H106 N19 O29 P2
369.6833^{+4}	y_{12}-H_2O^{+4}	C57 H100 N22 O22 P1	919.4156	PPTDKPGQD-NH_3	C40 H59 N10 O15	1682.2496^{+2}	MH-H_2O^{+2}	C132 H219 N43 O54 P3
369.9293^{+4}	y_{12}-NH_3^{+4}	C57 H99 N21 O23 P1	921.8948^{+2}	a_{12}-H_2O^{+2}	C74 H118 N21 O30 P2	1682.7416^{+2}	MH-NH_3^{+2}	C132 H218 N42 O55 P3
370.1721	PGQD-CO	C15 H24 N5 O6	922.2856	QIR(ADP-Ribosyl)-NH_3	C32 H50 N11 O17 P2	1682.7898	LLTPPTDKPGQDNRS(Phospho)-H_2O	C69 H113 N21 O26 P1
370.2024^{+2}	y_5^{+2}	C27 H56 N12 O10 P1	922.3868^{+2}	a_{12}-NH_3^{+2}	C74 H117 N20 O31 P2	1683.7738	LLTPPTDKPGQDNRS(Phospho)-NH_3	C69 H112 N20 O27 P1
370.2085	DKPG-CO	C16 H28 N5 O5	922.4142	QDNRS(Phospho)KL	C34 H61 N13 O15 P1	1687.8163	TPPTDKPGQDNRS(Phospho)KL-CO	C68 H116 N22 O26 P1
374.1860^{+4}	y_{12}^{+4}	C57 H102 N22 O23 P1	922.4629	TPPTDKPGQ	C40 H64 N11 O14	1687.8163	LTPPTDKPGQDNRS(Phospho)K-CO	C68 H116 N22 O26 P1
374.9537^{+4}	y_{13}-$H_3PO_4^{+4}$	C61 H106 N23 O21	924.4110^{+3}	a_{20}^{+3}	C110 H178 N35 O45 P2	1690.6874	b_{10}	C66 H106 N19 O29 P2
378.2354^{+2}	y_6-$H_3PO_4^{+2}$	C31 H59 N14 O8	925.4374	PTDKPGQDN-CO	C38 H61 N12 O15	1690.8983	y_{15}-H_3PO_4	C71 H120 N25 O23
379.1838^{+3}	y_9-H_2O^{+3}	C42 H76 N18 O17 P1	926.2805	R(ADP-Ribosyl)DL	C31 H50 N11 O18 P2	1691.2549^{+2}	MH^{+2}	C132 H221 N43 O55 P3
379.1976	TPPT-H_2O	C18 H27 N4 O5	926.2805	IR(ADP-Ribosyl)D	C31 H50 N11 O18 P2	1691.8225	y_{14}	C66 H116 N24 O26 P1
379.5118^{+3}	y_9-NH_3^{+3}	C42 H75 N17 O18 P1	927.7392^{+3}	b_{20}-H_2O^{+3}	C111 H176 N35 O45 P2	1697.8007	TPPTDKPGQDNRS(Phospho)KL-H_2O	C69 H114 N22 O26 P1
380.1565	PGQD-H_2O	C16 H22 N5 O6	928.0672^{+3}	b_{20}-NH_3^{+3}	C111 H175 N34 O46 P2	1697.8007	LTPPTDKPGQDNRS(Phospho)K-H_2O	C69 H114 N22 O26 P1
380.1928	DKPG-H_2O	C17 H26 N5 O5	930.9001^{+2}	a_{12}^{+2}	C74 H120 N21 O31 P2	1698.7847	LTPPTDKPGQDNRS(Phospho)K-NH_3	C69 H113 N21 O27 P1
381.1405	PGQD-NH_3	C16 H21 N4 O7	933.7427^{+3}	b_{20}^{+3}	C111 H178 N35 O46 P2	1698.7847	TPPTDKPGQDNRS(Phospho)KL-NH_3	C69 H113 N21 O27 P1
381.1769	DKPG-NH_3	C17 H25 N4 O6	935.4095	PGQDNRS(Phospho)K-CO	C34 H60 N14 O15 P1	1700.8003	LLTPPTDKPGQDNRS(Phospho)	C69 H115 N21 O27 P1
381.1897	S(Phospho)KL-CO	C14 H30 N4 O6 P1	935.4095	KPGQDNRS(Phospho)-CO	C34 H60 N14 O15 P1	1715.8112	TPPTDKPGQDNRS(Phospho)KL	C69 H116 N22 O27 P1
381.2496	LTPP-CO	C19 H33 N4 O4	935.4217	PTDKPGQDN-H_2O	C39 H59 N12 O15	1715.8112	LTPPTDKPGQDNRS(Phospho)K	C69 H116 N22 O27 P1
383.1925	PPTD-CO	C17 H27 N4 O6	935.5560	LLTPPTDKP-CO	C44 H75 N10 O12	1741.7346	a_{11}-H_2O	C70 H111 N20 O28 P2
383.2401	KPGQ-CO	C17 H31 N6 O4	935.8923^{+2}	b_{12}-H_2O^{+2}	C75 H118 N21 O31 P2	1742.7187	a_{11}-NH_3	C70 H110 N19 O29 P2
384.8793^{+3}	y_{10}-$H_3PO_4^{+3}$	C47 H82 N19 O15	936.3843^{+2}	b_{12}-NH_3^{+2}	C75 H117 N20 O32 P2	1745.7659	PQIR(ADP-Ribosyl)DLLTPPT-CO	C70 H115 N20 O28 P2
385.1873^{+3}	y_9^{+3}	C42 H78 N18 O18 P1	936.4058	PTDKPGQDN-NH_3	C39 H58 N11 O16	1747.7452	R(ADP-Ribosyl)DLLTPPTDKP-CO	C69 H113 N20 O29 P2
386.1783	DNR	C14 H24 N7 O6	936.4421	PPTDKPGQD	C40 H62 N11 O15	1755.7503	PQIR(ADP-Ribosyl)DLLTPPT-H_2O	C71 H113 N20 O28 P2
387.1623	GQDN-CO	C14 H23 N6 O7	936.4598^{+2}	y_{16}-H_2O^{+2}	C75 H128 N26 O28 P1	1756.7343	PQIR(ADP-Ribosyl)DLLTPPT-NH_3	C71 H112 N19 O29 P2
391.1741	S(Phospho)KL-H_2O	C15 H28 N4 O6 P1	936.9518^{+2}	y_{16}-NH_3^{+2}	C75 H127 N25 O29 P1	1757.7296	R(ADP-Ribosyl)DLLTPPTDKP-H_2O	C70 H111 N20 O29 P2
391.2340	LTPP-H_2O	C20 H31 N4 O4	939.3121	QIR(ADP-Ribosyl)	C32 H53 N12 O17 P2	1758.7136	R(ADP-Ribosyl)DLLTPPTDKP-NH_3	C70 H110 N19 O30 P2
392.1581	S(Phospho)KL-NH_3	C15 H27 N3 O7 P1	944.1060^{+3}	y_{20}-$H_3PO_4^{+3}$	C112 H187 N38 O44 P2	1759.7452	a_{11}	C70 H113 N20 O29 P2
393.1769	PPTD-H_2O	C18 H25 N4 O6	944.8975^{+2}	b_{12}^{+2}	C75 H120 N21 O32 P2	1763.7401	QIR(ADP-Ribosyl)DLLTPPTD-CO	C69 H113 N20 O30 P2
394.2085	KPGQ-NH_3	C18 H28 N5 O5	945.3938	PGQDNRS(Phospho)K-H_2O	C35 H58 N14 O15 P1	1763.7765	IR(ADP-Ribosyl)DLLTPPTDK-CO	C70 H117 N20 O29 P2
394.9452^{+4}	y_{13}-H_2O^{+4}	C61 H107 N23 O24 P1	945.3938	KPGQDNRS(Phospho)-H_2O	C35 H58 N14 O15 P1	1769.7296	b_{11}-H_2O	C71 H111 N20 O29 P2

395.1912^{+4}	y_{13}-NH_3^{+4}	C61 H106 N22 O25 P1	945.4651^{+2}	y_{16}^{+2}	C75 H130 N26 O29 P1	1770.7136	b_{11}-NH_3	C71 H110 N19 O30 P2
397.1466	GQDN-H_2O	C15 H21 N6 O7	945.5404	LLTPPTDKP-H_2O	C45 H73 N10 O12	1770.8647	y_{15}-H_2O	C71 H121 N25 O26 P1
397.2082	TPPT	C18 H29 N4 O6	946.3778	PGQDNRS(Phospho)K-NH_3	C35 H57 N13 O16 P1	1771.8487	y_{15}-NH_3	C71 H120 N24 O27 P1
397.2809	LLTP-CO	C20 H37 N4 O4	946.3778	KPGQDNRS(Phospho)-NH_3	C35 H57 N13 O16 P1	1773.7245	QIR(ADP-Ribosyl)DLLTPPTD-H_2O	C70 H111 N20 O30 P2
398.1306	GQDN-NH_3	C15 H20 N5 O8	946.5244	LLTPPTDKP-NH_3	C45 H72 N9 O13	1773.7609	IR(ADP-Ribosyl)DLLTPPTDK-H_2O	C71 H115 N20 O29 P2
398.1670	PGQD	C16 H24 N5 O7	947.4182^{+3}	a_{21}-$H_3PO_4^{+3}$	C113 H181 N36 O46 P2	1773.7609	PQIR(ADP-Ribosyl)DLLTPPT	C71 H115 N20 O29 P2
398.2034	DKPG	C17 H28 N5 O6	950.4568	y_7-H_2O	C35 H65 N15 O14 P1	1774.7085	QIR(ADP-Ribosyl)DLLTPPTD-NH_3	C70 H110 N19 O31 P2
399.2169^{+4}	y_{14}-$H_3PO_4^{+4}$	C66 H113 N24 O22	951.4408	GQDNRS(Phospho)KL-CO	C35 H64 N14 O15 P1	1774.7449	IR(ADP-Ribosyl)DLLTPPTDK-NH_3	C71 H114 N19 O30 P2
399.2714	y_3-NH_3	C18 H35 N6 O4	951.4408	y_7-NH_3	C35 H64 N14 O15 P1	1775.7401	R(ADP-Ribosyl)DLLTPPTDKP	C70 H113 N20 O30 P2
399.4479^{+4}	y_{13}^{+4}	C61 H109 N23 O25 P1	953.0187^{+2}	y_{17}-$H_3PO_4^{+2}$	C81 H138 N27 O26	1787.7401	b_{11}	C71 H113 N20 O30 P2
407.2653	LLTP-H_2O	C21 H35 N4 O4	953.4323	PTDKPGQDN	C39 H61 N12 O16	1787.8324	DLLTPPTDKPGQDNRS(Phospho)-CO	C72 H120 N22 O29 P1
408.2241	a_4-H_2O	C19 H30 N5 O5	953.5302	DLLTPPTDK-CO	C43 H73 N10 O14	1788.8752	y_{15}	C71 H123 N25 O27 P1
409.1847	S(Phospho)KL	C15 H30 N4 O7 P1	956.7498^{+3}	b_{21}-$H_3PO_4^{+3}$	C114 H181 N36 O47 P2	1791.7350	QIR(ADP-Ribosyl)DLLTPPTD	C70 H113 N20 O31 P2
409.2082	a_4-NH_3	C19 H29 N4 O6	961.4251	GQDNRS(Phospho)KL-H_2O	C36 H62 N14 O15 P1	1791.7714	IR(ADP-Ribosyl)DLLTPPTDK	C71 H117 N20 O30 P2
409.2445	LTPP	C20 H33 N4 O5	962.4091	GQDNRS(Phospho)KL-NH_3	C36 H61 N13 O16 P1	1791.9460	y_{16}-H_3PO_4	C75 H127 N26 O25
410.1548	NRS(Phospho)-CO	C12 H25 N7 O7 P1	963.4044	PGQDNRS(Phospho)K	C35 H60 N14 O16 P1	1797.8167	DLLTPPTDKPGQDNRS(Phospho)-H_2O	C73 H118 N22 O29 P1
411.1874	PPTD	C18 H27 N4 O7	963.4044	KPGQDNRS(Phospho)	C35 H60 N14 O16 P1	1798.8007	DLLTPPTDKPGQDNRS(Phospho)-NH_3	C73 H117 N21 O30 P1
411.2350	KPGQ	C18 H31 N6 O5	963.5146	DLLTPPTDK-H_2O	C44 H71 N10 O14	1800.9004	LTPPTDKPGQDNRS(Phospho)KL-CO	C74 H127 N23 O27 P1
411.5347^{+3}	y_{10}-H_2O^{+3}	C47 H83 N19 O18 P1	963.5510	LLTPPTDKP	C45 H75 N10 O13	1800.9004	LLTPPTDKPGQDNRS(Phospho)K-CO	C74 H127 N23 O27 P1
411.8627^{+3}	y_{10}-NH_3^{+3}	C47 H82 N18 O19 P1	964.4986	DLLTPPTDK-NH_3	C44 H70 N9 O15	1804.7667	R(ADP-Ribosyl)DLLTPPTDKPG-CO	C71 H116 N21 O30 P2
414.2347	TDKP-CO	C18 H32 N5 O6	968.4673	y_7	C35 H67 N15 O15 P1	1810.8847	LTPPTDKPGQDNRS(Phospho)KL-H_2O	C75 H125 N23 O27 P1
414.2347	PTDK-CO	C18 H32 N5 O6	970.7614^{+3}	y_{20}-H_2O^{+3}	C112 H188 N38 O47 P3	1810.8847	LLTPPTDKPGQDNRS(Phospho)K-H_2O	C75 H125 N23 O27 P1
415.1572	GQDN	C15 H23 N6 O8	971.0894^{+3}	y_{20}-NH_3^{+3}	C112 H187 N37 O48 P3	1811.8688	LTPPTDKPGQDNRS(Phospho)KL-NH_3	C75 H124 N22 O28 P1
415.2551	DLLT-CO	C19 H35 N4 O6	974.0736^{+3}	a_{21}-H_2O^{+3}	C113 H182 N36 O49 P3	1811.8688	LLTPPTDKPGQDNRS(Phospho)K-NH_3	C75 H124 N22 O28 P1
416.2980	y_3	C18 H38 N7 O4	974.4016^{+3}	a_{21}-NH_3^{+3}	C113 H181 N35 O50 P3	1814.7510	R(ADP-Ribosyl)DLLTPPTDKPG-H_2O	C72 H114 N21 O30 P2
417.5382^{+3}	y_{10}^{+3}	C47 H85 N19 O19 P1	976.7649^{+3}	y_{20}^{+3}	C112 H190 N38 O48 P3	1815.7350	R(ADP-Ribosyl)DLLTPPTDKPG-NH_3	C72 H113 N20 O31 P2
418.7106^{+2}	y_6-NH_3^{+2}	C31 H59 N13 O12 P1	979.4083^{+2}	a_{13}-H_2O^{+2}	C78 H123 N22 O33 P2	1815.8273	DLLTPPTDKPGQDNRS(Phospho)	C73 H120 N22 O30 P1
419.2084^{+4}	y_{14}-H_2O^{+4}	C66 H114 N24 O25 P1	979.4357	GQDNRS(Phospho)KL	C36 H64 N14 O16 P1	1828.8953	LLTPPTDKPGQDNRS(Phospho)K	C75 H127 N23 O28 P1
419.4544^{+4}	y_{14}-NH_3^{+4}	C66 H113 N23 O26 P1	979.9003^{+2}	a_{13}-NH_3^{+2}	C78 H122 N21 O34 P2	1828.8953	LTPPTDKPGQDNRS(Phospho)KL	C75 H127 N23 O28 P1
420.1391	NRS(Phospho)-H_2O	C13 H23 N7 O7 P1	980.0772^{+3}	a_{21}^{+3}	C113 H184 N36 O50 P3	1832.7616	R(ADP-Ribosyl)DLLTPPTDKPG	C72 H116 N21 O31 P2
421.1231	NRS(Phospho)-NH_3	C13 H22 N6 O8 P1	981.5251	DLLTPPTDK	C44 H73 N10 O15	1842.7823	a_{12}-H_2O	C74 H118 N21 O30 P2
423.4800^{+4}	y_{15}-$H_3PO_4^{+4}$	C71 H120 N25 O23	981.8006^{+3}	y_{21}-$H_3PO_4^{+3}$	C118 H198 N39 O45 P2	1843.7663	a_{12}-NH_3	C74 H117 N20 O31 P2
423.7111^{+4}	y_{14}^{+4}	C66 H116 N24 O26 P1	983.4053^{+3}	b_{21}-H_2O^{+3}	C114 H182 N36 O50 P3	1860.7929	PQIR(ADP-Ribosyl)DLLTPPTD-CO	C74 H120 N21 O31 P2
424.2068	RS(Phospho)K-CO	C14 H31 N7 O6 P1	983.7333^{+3}	b_{21}-NH_3^{+3}	C114 H181 N35 O51 P3	1860.7929	a_{12}	C74 H120 N21 O31 P2
424.2191	TDKP-H_2O	C19 H30 N5 O6	984.4857	TDKPGQDNR-CO	C39 H66 N15 O15	1860.8293	IR(ADP-Ribosyl)DLLTPPTDKP-CO	C75 H124 N21 O30 P2
424.2191	PTDK-H_2O	C19 H30 N5 O6	988.4136^{+2}	a_{13}^{+2}	C78 H125 N22 O34 P2	1870.7772	b_{12}-H_2O	C75 H118 N21 O31 P2
425.2031	TDKP-NH_3	C19 H29 N4 O7	989.4088^{+3}	b_{21}^{+3}	C114 H184 N36 O51 P3	1870.7772	PQIR(ADP-Ribosyl)DLLTPPTD-H_2O	C75 H118 N21 O31 P2
425.2031	PTDK-NH_3	C19 H29 N4 O7	990.1165^{+3}	a_{22}-$H_3PO_4^{+3}$	C119 H193 N38 O47 P2	1870.8136	IR(ADP-Ribosyl)DLLTPPTDKP-H_2O	C76 H122 N21 O30 P2
425.2395	DLLT-H_2O	C20 H33 N4 O6	992.5775	LLTPPTDKPG-CO	C46 H78 N11 O13	1871.7613	b_{12}-NH_3	C75 H117 N20 O32 P2
425.2758	LLTP	C21 H37 N4 O5	993.0018^{+2}	y_{17}-H_2O^{+2}	C81 H139 N27 O29 P1	1871.7613	PQIR(ADP-Ribosyl)DLLTPPTD-NH_3	C75 H117 N20 O32 P2
426.2347	a_4	C19 H32 N5 O6	993.4057^{+2}	b_{13}-H_2O^{+2}	C79 H123 N22 O34 P2	1871.7976	IR(ADP-Ribosyl)DLLTPPTDKP-NH_3	C76 H121 N20 O31 P2
427.2238^{+2}	y_6^{+2}	C31 H62 N14 O12 P1	993.4939^{+2}	y_{17}-NH_3^{+2}	C81 H138 N26 O30 P1	1871.9124	y_{16}-H_2O	C75 H128 N26 O28 P1
427.5776^{+3}	y_{11}-$H_3PO_4^{+3}$	C53 H94 N21 O16	993.8977^{+2}	b_{13}-NH_3^{+2}	C79 H122 N21 O35 P2	1872.8964	y_{16}-NH_3	C75 H127 N25 O29 P1

434.1911	RS(Phospho)K-H_2O	C15 H29 N7 O6 P1	994.4701	TDKPGQDNR-H_2O	C40 H64 N15 O15	1888.7878	b_{12}	C75 H120 N21 O32 P2
435.1752	RS(Phospho)K-NH_3	C15 H28 N6 O7 P1	995.4541	TDKPGQDNR-NH_3	C40 H63 N14 O16	1888.7878	PQIR(ADP-Ribosyl)DLLTPPTD	C75 H120 N21 O32 P2
435.7489^{+2}	y_7-$H_3PO_4^{+2}$	C35 H64 N15 O11	998.5490	y_8-H_3PO_4	C40 H72 N17 O13	1888.8242	IR(ADP-Ribosyl)DLLTPPTDKP	C76 H124 N21 O31 P2
436.2191	b_4-H_2O	C20 H30 N5 O6	999.4482^{+3}	b_{22}-$H_3PO_4^{+3}$	C120 H193 N38 O48 P2	1889.9229	y_{16}	C75 H130 N26 O29 P1
437.2031	b_4-NH_3	C20 H29 N4 O7	1002.0071^{+2}	y_{17}^{+2}	C81 H141 N27 O30 P1	1891.8351	QIR(ADP-Ribosyl)DLLTPPTDK-CO	C75 H125 N22 O31 P2
438.1497	NRS(Phospho)	C13 H25 N7 O8 P1	1002.4110^{+2}	b_{13}^{+2}	C79 H125 N22 O35 P2	1901.8194	QIR(ADP-Ribosyl)DLLTPPTDK-H_2O	C76 H123 N22 O31 P2
442.2296	TDKP	C19 H32 N5 O7	1002.5619	LLTPPTDKPG-H_2O	C47 H76 N11 O13	1902.8035	QIR(ADP-Ribosyl)DLLTPPTDK-NH_3	C76 H122 N21 O32 P2
442.2296	PTDK	C19 H32 N5 O7	1003.5459	LLTPPTDKPG-NH_3	C47 H75 N10 O14	1905.0301	y_{17}-H_3PO_4	C81 H138 N27 O26
443.2500	DLLT	C20 H35 N4 O7	1007.5520	LTPPTDKPGQ-CO	C45 H75 N12 O14	1913.9845	LLTPPTDKPGQDNRS(Phospho)KL-CO	C80 H138 N24 O28 P1
443.4716^{+4}	y_{15}-H_2O^{+4}	C71 H121 N25 O26 P1	1008.3700	PQIR(ADP-Ribosyl)-CO	C36 H60 N13 O17 P2	1915.9273	DLLTPPTDKPGQDNRS(Phospho)K-CO	C78 H132 N24 O30 P1
443.7176^{+4}	y_{15}-NH_3^{+4}	C71 H120 N24 O27 P1	1008.4561^{+3}	y_{21}-H_2O^{+3}	C118 H199 N39 O48 P3	1917.8507	IR(ADP-Ribosyl)DLLTPPTDKPG-CO	C77 H127 N22 O31 P2
447.9743^{+4}	y_{15}^{+4}	C71 H123 N25 O27 P1	1008.7841^{+3}	y_{21}-NH_3^{+3}	C118 H198 N38 O49 P3	1919.8300	QIR(ADP-Ribosyl)DLLTPPTDK	C76 H125 N22 O32 P2
448.7420^{+4}	y_{16}-$H_3PO_4^{+4}$	C75 H127 N26 O25	1009.4949	TPPTDKPGQD-CO	C43 H69 N12 O16	1923.9688	LLTPPTDKPGQDNRS(Phospho)KL-H_2O	C81 H136 N24 O28 P1
452.2017	RS(Phospho)K	C15 H31 N7 O7 P1	1009.5607^{+2}	y_{18}-$H_3PO_4^{+2}$	C87 H149 N28 O27	1924.9528	LLTPPTDKPGQDNRS(Phospho)KL-NH_3	C81 H135 N23 O29 P1
454.2296	b_4	C20 H32 N5 O7	1011.3697	IR(ADP-Ribosyl)DL-CO	C36 H61 N12 O18 P2	1925.9117	DLLTPPTDKPGQDNRS(Phospho)K-H_2O	C79 H130 N24 O30 P1
454.2330^{+3}	y_{11}-H_2O^{+3}	C53 H95 N21 O19 P1	1011.3697	R(ADP-Ribosyl)DLL-CO	C36 H61 N12 O18 P2	1926.8957	DLLTPPTDKPGQDNRS(Phospho)K-NH_3	C79 H129 N23 O31 P1
454.5610^{+3}	y_{11}-NH_3^{+3}	C53 H94 N20 O20 P1	1012.4806	TDKPGQDNR	C40 H66 N15 O16	1927.8351	IR(ADP-Ribosyl)DLLTPPTDKPG-H_2O	C78 H125 N22 O31 P2
460.2365^{+3}	y_{11}^{+3}	C53 H97 N21 O20 P1	1014.4596^{+3}	y_{21}^{+3}	C118 H201 N39 O49 P3	1928.8191	IR(ADP-Ribosyl)DLLTPPTDKPG-NH_3	C78 H124 N21 O32 P2
465.9199^{+3}	y_{12}-$H_3PO_4^{+3}$	C57 H99 N22 O19	1016.7720^{+3}	a_{22}-H_2O^{+3}	C119 H194 N38 O50 P3	1932.8252	R(ADP-Ribosyl)DLLTPPTDKPGQ-CO	C76 H124 N23 O32 P2
468.7335^{+4}	y_{16}-H_2O^{+4}	C75 H128 N26 O28 P1	1017.1000^{+3}	a_{22}-NH_3^{+3}	C119 H193 N37 O51 P3	1941.9794	LLTPPTDKPGQDNRS(Phospho)KL	C81 H138 N24 O29 P1
468.9795^{+4}	y_{16}-NH_3^{+4}	C75 H127 N25 O29 P1	1017.5364	LTPPTDKPGQ-H_2O	C46 H73 N12 O14	1942.8096	R(ADP-Ribosyl)DLLTPPTDKPGQ-H_2O	C77 H122 N23 O32 P2
471.2562	TDKPG-CO	C20 H35 N6 O7	1018.5204	LTPPTDKPGQ-NH_3	C46 H72 N11 O15	1943.7936	R(ADP-Ribosyl)DLLTPPTDKPGQ-NH_3	C77 H121 N22 O33 P2
473.2362^{+4}	y_{16}^{+4}	C75 H130 N26 O29 P1	1019.3384	PQIR(ADP-Ribosyl)-NH_3	C37 H57 N12 O18 P2	1943.9222	DLLTPPTDKPGQDNRS(Phospho)K	C79 H132 N24 O31 P1
475.7320^{+2}	y_7-H_2O^{+2}	C35 H65 N15 O14 P1	1019.4793	TPPTDKPGQD-H_2O	C44 H67 N12 O16	1945.8456	IR(ADP-Ribosyl)DLLTPPTDKPG	C78 H127 N22 O32 P2
476.2240^{+2}	y_7-NH_3^{+2}	C35 H64 N14 O15 P1	1020.4633	TPPTDKPGQD-NH_3	C44 H66 N11 O17	1957.8093	a_{13}-H_2O	C78 H123 N22 O33 P2
477.0130^{+4}	y_{17}-$H_3PO_4^{+4}$	C81 H138 N27 O26	1020.5724	LLTPPTDKPG	C47 H78 N11 O14	1958.7933	a_{13}-NH_3	C78 H122 N21 O34 P2
481.2405	TDKPG-H_2O	C21 H33 N6 O7	1021.3540	R(ADP-Ribosyl)DLL-H_2O	C37 H59 N12 O18 P2	1960.8202	R(ADP-Ribosyl)DLLTPPTDKPGQ	C77 H124 N23 O33 P2
482.2245	TDKPG-NH_3	C21 H32 N5 O8	1021.3540	IR(ADP-Ribosyl)DL-H_2O	C37 H59 N12 O18 P2	1975.8198	a_{13}	C78 H125 N22 O34 P2
482.2973	LTPPT-CO	C23 H40 N5 O6	1022.3380	R(ADP-Ribosyl)DLL-NH_3	C37 H58 N11 O19 P2	1984.9964	y_{17}-H_2O	C81 H139 N27 O29 P1
484.2150	PGQDN-CO	C19 H30 N7 O8	1022.3380	IR(ADP-Ribosyl)DL-NH_3	C37 H58 N11 O19 P2	1985.8042	b_{13}-H_2O	C79 H123 N22 O34 P2
484.2402	TPPTD-CO	C21 H34 N5 O8	1022.4901	PPTDKPGQDN-CO	C43 H68 N13 O16	1985.9804	y_{17}-NH_3	C81 H138 N26 O30 P1
484.7373^{+2}	y_7^{+2}	C35 H67 N15 O15 P1	1022.7755^{+3}	a_{22}^{+3}	C119 H196 N38 O51 P3	1986.7882	b_{13}-NH_3	C79 H122 N21 O35 P2
485.3194	y_4-H_3PO_4	C21 H41 N8 O5	1024.4868^{+3}	y_{22}-$H_3PO_4^{+3}$	C123 H206 N41 O47 P2	1988.8878	QIR(ADP-Ribosyl)DLLTPPTDKP-CO	C80 H132 N23 O32 P2
486.2419	QDNR-CO	C18 H32 N9 O7	1026.1036^{+3}	b_{22}-H_2O^{+3}	C120 H194 N38 O51 P3	1988.8878	PQIR(ADP-Ribosyl)DLLTPPTDK-CO	C80 H132 N23 O32 P2
492.2817	LTPPT-H_2O	C24 H38 N5 O6	1026.3442	QIR(ADP-Ribosyl)D-CO	C35 H58 N13 O19 P2	1998.8722	QIR(ADP-Ribosyl)DLLTPPTDKP-H_2O	C81 H130 N23 O32 P2
492.5753^{+3}	y_{12}-H_2O^{+3}	C57 H100 N22 O22 P1	1026.4316^{+3}	b_{22}-NH_3^{+3}	C120 H193 N37 O52 P3	1998.8722	PQIR(ADP-Ribosyl)DLLTPPTDK-H_2O	C81 H130 N23 O32 P2
492.9033^{+3}	y_{12}-NH_3^{+3}	C57 H99 N21 O23 P1	1027.8112^{+3}	a_{23}-$H_3PO_4^{+3}$	C125 H204 N39 O48 P2	1999.8562	QIR(ADP-Ribosyl)DLLTPPTDKP-NH_3	C81 H129 N22 O33 P2
494.1994	PGQDN-H_2O	C20 H28 N7 O8	1032.1071^{+3}	b_{22}^{+3}	C120 H196 N38 O52 P3	1999.8562	PQIR(ADP-Ribosyl)DLLTPPTDK-NH_3	C81 H129 N22 O33 P2
494.2245	TPPTD-H_2O	C22 H32 N5 O8	1032.4745	PPTDKPGQDN-H_2O	C44 H66 N13 O16	2003.0070	y_{17}	C81 H141 N27 O30 P1
494.3337	LLTPP-CO	C25 H44 N5 O5	1033.4585	PPTDKPGQDN-NH_3	C44 H65 N12 O17	2003.8147	b_{13}	C79 H125 N22 O35 P2
495.1834	PGQDN-NH_3	C20 H27 N6 O9	1035.5469	LTPPTDKPGQ	C46 H75 N12 O15	2016.8828	QIR(ADP-Ribosyl)DLLTPPTDKP	C81 H132 N23 O33 P2
496.2263	QDNR-H_2O	C19 H30 N9 O7	1036.3285	QIR(ADP-Ribosyl)D-H_2O	C36 H56 N13 O19 P2	2016.8828	PQIR(ADP-Ribosyl)DLLTPPTDK	C81 H132 N23 O33 P2
497.0046^{+4}	y_{17}-H_2O^{+4}	C81 H139 N27 O29 P1	1036.3649	PQIR(ADP-Ribosyl)	C37 H60 N13 O18 P2	2018.1141	y_{18}-H_3PO_4	C87 H149 N28 O27

497.2103	**QDNR-NH$_3$**	C19 H29 N8 O8	1037.1428^{+3}	**b$_{23}$-H$_3$PO$_4^{+3}$**	C126 H204 N39 O49 P2	2029.0114	**DLLTPPTDKPGQDNRS(Phospho)KL-CO**	C84 H143 N25 O31 P1
497.2506^{+4}	**y$_{17}$-NH$_3^{+4}$**	C81 H138 N26 O30 P1	1037.3125	**QIR(ADP-Ribosyl)D-NH$_3$**	C36 H55 N12 O20 P2	2038.9957	**DLLTPPTDKPGQDNRS(Phospho)KL-H$_2$O**	C85 H141 N25 O31 P1
498.2671	**KPGQD-CO**	C21 H36 N7 O7	1037.4898	**TPPTDKPGQD**	C44 H69 N12 O17	2039.9798	**DLLTPPTDKPGQDNRS(Phospho)KL-NH$_3$**	C85 H140 N24 O32 P1
498.2671	**DKPGQ-CO**	C21 H36 N7 O7	1038.1106^{+3}	**b$_{22}$+H$_2$O^{+3}**	C120 H198 N38 O53 P3	2045.9093	**IR(ADP-Ribosyl)DLLTPPTDKPGQ-CO**	C82 H135 N24 O33 P2
498.5789^{+3}	**y$_{12}^{+3}$**	C57 H102 N22 O23 P1	1039.3646	**IR(ADP-Ribosyl)DL**	C37 H61 N12 O19 P2	2045.9093	**QIR(ADP-Ribosyl)DLLTPPTDKPG-CO**	C82 H135 N24 O33 P2
499.2511	**TDKPG**	C21 H35 N6 O8	1039.3646	**R(ADP-Ribosyl)DLL**	C37 H61 N12 O19 P2	2047.8522	**R(ADP-Ribosyl)DLLTPPTDKPGQD-CO**	C80 H129 N24 O35 P2
499.6025^{+3}	**y$_{13}$-H$_3$PO$_4^{+3}$**	C61 H106 N23 O21	1043.4558^{+2}	**a$_{14}$-H$_2$O^{+2}**	C84 H135 N24 O34 P2	2055.8937	**QIR(ADP-Ribosyl)DLLTPPTDKPG-H$_2$O**	C83 H133 N24 O33 P2
499.7781^{+2}	**y$_8$-H$_3$PO$_4^{+2}$**	C40 H72 N17 O13	1043.9478^{+2}	**a$_{14}$-NH$_3^{+2}$**	C84 H134 N23 O35 P2	2055.8937	**IR(ADP-Ribosyl)DLLTPPTDKPGQ-H$_2$O**	C83 H133 N24 O33 P2
501.5072^{+4}	**y$_{17}^{+4}$**	C81 H141 N27 O30 P1	1048.4935	**PGQDNRS(Phospho)KL-CO**	C40 H71 N15 O16 P1	2056.8777	**QIR(ADP-Ribosyl)DLLTPPTDKPG-NH$_3$**	C83 H132 N23 O34 P2
504.3180	**LLTPP-H$_2$O**	C26 H42 N5 O5	1049.5439^{+2}	**y$_{18}$-H$_2$O^{+2}**	C87 H150 N28 O30 P1	2056.8777	**IR(ADP-Ribosyl)DLLTPPTDKPGQ-NH$_3$**	C83 H132 N23 O34 P2
505.2840^{+4}	**y$_{18}$-H$_3$PO$_4^{+4}$**	C87 H149 N28 O27	1050.0359^{+2}	**y$_{18}$-NH$_3^{+2}$**	C87 H149 N27 O31 P1	2057.0063	**DLLTPPTDKPGQDNRS(Phospho)KL**	C85 H143 N25 O32 P1
508.2514	**DKPGQ-H$_2$O**	C22 H34 N7 O7	1050.4364	**DKPGQDNRS(Phospho)-CO**	C38 H65 N15 O18 P1	2057.8365	**R(ADP-Ribosyl)DLLTPPTDKPGQD-H$_2$O**	C81 H127 N24 O35 P2
508.2514	**KPGQD-H$_2$O**	C22 H34 N7 O7	1050.4851	**PPTDKPGQDN**	C44 H68 N13 O17	2058.8206	**R(ADP-Ribosyl)DLLTPPTDKPGQD-NH$_3$**	C81 H126 N23 O36 P2
509.2354	**KPGQD-NH$_3$**	C22 H33 N6 O8	1050.5830	**DLLTPPTDKP-CO**	C48 H80 N11 O15	2073.9042	**QIR(ADP-Ribosyl)DLLTPPTDKPG**	C83 H135 N24 O34 P2
509.2354	**DKPGQ-NH$_3$**	C22 H33 N6 O8	1051.1423^{+3}	**y$_{22}$-H$_2$O^{+3}**	C123 H207 N41 O50 P3	2073.9042	**IR(ADP-Ribosyl)DLLTPPTDKPGQ**	C83 H135 N24 O34 P2
510.2922	**LTPPT**	C24 H40 N5 O7	1051.4703^{+3}	**y$_{22}$-NH$_3^{+3}$**	C123 H206 N40 O51 P3	2075.8471	**R(ADP-Ribosyl)DLLTPPTDKPGQD**	C81 H129 N24 O36 P2
511.2875	**PTDKP-CO**	C23 H39 N6 O7	1052.4610^{+2}	**a$_{14}^{+2}$**	C84 H137 N24 O35 P2	2085.9042	**a$_{14}$-H$_2$O**	C84 H135 N24 O34 P2
511.2875	**PPTDK-CO**	C23 H39 N6 O7	1054.3391	**QIR(ADP-Ribosyl)D**	C36 H58 N13 O20 P2	2085.9406	**PQIR(ADP-Ribosyl)DLLTPPTDKP-CO**	C85 H139 N24 O33 P2
512.2100	**PGQDN**	C20 H30 N7 O9	1054.4667^{+3}	**a$_{23}$-H$_2$O^{+3}**	C125 H205 N39 O51 P3	2086.8882	**a$_{14}$-NH$_3$**	C84 H134 N23 O35 P2
512.2351	**TPPTD**	C22 H34 N5 O9	1054.7947^{+3}	**a$_{23}$-NH$_3^{+3}$**	C125 H204 N38 O52 P3	2095.9250	**PQIR(ADP-Ribosyl)DLLTPPTDKP-H$_2$O**	C86 H137 N24 O33 P2
512.3079	**DLLTP-CO**	C24 H42 N5 O7	1055.5705	**y$_9$-H$_3$PO$_4$**	C42 H75 N18 O14	2096.9090	**PQIR(ADP-Ribosyl)DLLTPPTDKP-NH$_3$**	C86 H136 N23 O34 P2
514.2368	**QDNR**	C19 H32 N9 O8	1056.8378^{+3}	**y$_{23}$-H$_3$PO$_4^{+3}$**	C128 H213 N42 O48 P2	2098.0805	**y$_{18}$-H$_2$O**	C87 H150 N28 O30 P1
521.2718	**PTDKP-H$_2$O**	C24 H37 N6 O7	1057.1458^{+3}	**y$_{22}^{+3}$**	C123 H209 N41 O51 P3	2099.0645	**y$_{18}$-NH$_3$**	C87 H149 N27 O31 P1
521.2718	**PPTDK-H$_2$O**	C24 H37 N6 O7	1057.4532^{+2}	**b$_{14}$-H$_2$O^{+2}**	C85 H135 N24 O35 P2	2103.9148	**a$_{14}$**	C84 H137 N24 O35 P2
522.2558	**PTDKP-NH$_3$**	C24 H36 N5 O8	1057.9452^{+2}	**b$_{14}$-NH$_3^{+2}$**	C85 H134 N23 O36 P2	2113.8991	**b$_{14}$-H$_2$O**	C85 H135 N24 O35 P2
522.2558	**PPTDK-NH$_3$**	C24 H36 N5 O8	1058.4779	**PGQDNRS(Phospho)KL-H$_2$O**	C41 H69 N15 O16 P1	2113.9355	**PQIR(ADP-Ribosyl)DLLTPPTDKP**	C86 H139 N24 O34 P2
522.2922	**DLLTP-H$_2$O**	C25 H40 N5 O7	1058.5492^{+2}	**y$_{18}^{+2}$**	C87 H152 N28 O31 P1	2114.8832	**b$_{14}$-NH$_3$**	C85 H134 N23 O36 P2
522.3286	**LLTPP**	C26 H44 N5 O6	1059.4619	**PGQDNRS(Phospho)KL-NH$_3$**	C41 H68 N14 O17 P1	2116.0910	**y$_{18}$**	C87 H152 N28 O31 P1
525.1817	**DNRS(Phospho)-CO**	C16 H30 N8 O10 P1	1060.4208	**DKPGQDNRS(Phospho)-H$_2$O**	C39 H63 N15 O18 P1	2131.9097	**b$_{14}$**	C85 H137 N24 O36 P2
525.2756^{+4}	**y$_{18}$-H$_2$O^{+4}**	C87 H150 N28 O30 P1	1060.4702^{+3}	**a$_{23}^{+3}$**	C125 H207 N39 O52 P3	2133.1411	**y$_{19}$-H$_3$PO$_4$**	C91 H154 N29 O30
525.5216^{+4}	**y$_{18}$-NH$_3^{+4}$**	C87 H149 N27 O31 P1	1060.5673	**DLLTPPTDKP-H$_2$O**	C49 H78 N11 O15	2142.9621	**PQIR(ADP-Ribosyl)DLLTPPTDKPG-CO**	C87 H142 N25 O34 P2
526.2579^{+3}	**y$_{13}$-H$_2$O^{+3}**	C61 H107 N23 O24 P1	1061.4048	**DKPGQDNRS(Phospho)-NH$_3$**	C39 H62 N14 O19 P1	2152.9464	**PQIR(ADP-Ribosyl)DLLTPPTDKPG-H$_2$O**	C88 H140 N25 O34 P2
526.2620	**KPGQD**	C22 H36 N7 O8	1061.5514	**DLLTPPTDKP-NH$_3$**	C49 H77 N10 O16	2153.9304	**PQIR(ADP-Ribosyl)DLLTPPTDKPG-NH$_3$**	C88 H139 N24 O35 P2
526.2620	**DKPGQ**	C22 H36 N7 O8	1063.5044	**KPGQDNRS(Phospho)K-CO**	C40 H72 N16 O16 P1	2160.9363	**IR(ADP-Ribosyl)DLLTPPTDKPGQD-CO**	C86 H140 N25 O36 P2
526.5859^{+3}	**y$_{13}$-NH$_3^{+3}$**	C61 H106 N22 O25 P1	1063.7983^{+3}	**b$_{23}$-H$_2$O^{+3}**	C126 H205 N39 O52 P3	2161.8951	**R(ADP-Ribosyl)DLLTPPTDKPGQDN-CO**	C84 H135 N26 O37 P2
528.2889^{+2}	**y$_9$-H$_3$PO$_4^{+2}$**	C42 H75 N18 O14	1064.1263^{+3}	**b$_{23}$-NH$_3^{+3}$**	C126 H204 N38 O53 P3	2170.9206	**IR(ADP-Ribosyl)DLLTPPTDKPGQD-H$_2$O**	C87 H138 N25 O36 P2
529.7782^{+4}	**y$_{18}^{+4}$**	C87 H152 N28 O31 P1	1066.4585^{+2}	**b$_{14}^{+2}$**	C85 H137 N24 O36 P2	2170.9570	**PQIR(ADP-Ribosyl)DLLTPPTDKPG**	C88 H142 N25 O35 P2
531.9534^{+3}	**y$_{14}$-H$_3$PO$_4^{+3}$**	C66 H113 N24 O22	1067.0742^{+2}	**y$_{19}$-H$_3$PO$_4^{+2}$**	C91 H154 N29 O30	2171.8795	**R(ADP-Ribosyl)DLLTPPTDKPGQDN-H$_2$O**	C85 H133 N26 O37 P2
532.2614^{+3}	**y$_{13}^{+3}$**	C61 H109 N23 O25 P1	1069.8018^{+3}	**b$_{23}^{+3}$**	C126 H207 N39 O53 P3	2171.9046	**IR(ADP-Ribosyl)DLLTPPTDKPGQD-NH$_3$**	C87 H137 N24 O37 P2
534.0407^{+4}	**y$_{19}$-H$_3$PO$_4^{+4}$**	C91 H154 N29 O30	1073.4888	**KPGQDNRS(Phospho)K-H$_2$O**	C41 H70 N16 O16 P1	2172.8635	**R(ADP-Ribosyl)DLLTPPTDKPGQDN-NH$_3$**	C85 H132 N25 O38 P2
535.1661	**DNRS(Phospho)-H$_2$O**	C17 H28 N8 O10 P1	1074.4728	**KPGQDNRS(Phospho)K-NH$_3$**	C41 H69 N15 O17 P1	2173.9679	**QIR(ADP-Ribosyl)DLLTPPTDKPGQ-CO**	C87 H143 N26 O35 P2
536.1501	**DNRS(Phospho)-NH$_3$**	C17 H27 N7 O11 P1	1075.8053^{+3}	**b$_{23}$+H$_2$O^{+3}**	C126 H209 N39 O54 P3	2182.9570	**a$_{15}$-H$_2$O**	C89 H142 N25 O35 P2
537.2909	**RS(Phospho)KL-CO**	C20 H42 N8 O7 P1	1076.4884	**PGQDNRS(Phospho)KL**	C41 H71 N15 O17 P1	2183.9410	**a$_{15}$-NH$_3$**	C89 H141 N24 O36 P2

538.2497	NRS(Phospho)K-CO	C18 H37 N9 O8 P1	1078.4313	DKPGQDNRS(Phospho)	C39 H65 N15 O19 P1	2183.9522	QIR(ADP-Ribosyl)DLLTPPTDKPGQ-H_2O	C88 H141 N26 O35 P2
539.2824	PTDKP	C24 H39 N6 O8	1078.5153	y_8-H_2O	C40 H73 N17 O16 P1	2184.9363	QIR(ADP-Ribosyl)DLLTPPTDKPGQ-NH_3	C88 H140 N25 O36 P2
539.2824	PPTDK	C24 H39 N6 O8	1078.5779	DLLTPPTDKP	C49 H80 N11 O16	2188.9312	IR(ADP-Ribosyl)DLLTPPTDKPGQD	C87 H140 N25 O37 P2
539.7613^{+2}	y_8-H_2O^{+2}	C40 H73 N17 O16 P1	1079.4993	y_8-NH_3	C40 H72 N16 O17 P1	2189.8900	R(ADP-Ribosyl)DLLTPPTDKPGQDN	C85 H135 N26 O38 P2
540.2533^{+2}	y_8-NH_3^{+2}	C40 H72 N16 O17 P1	1081.5385	PTDKPGQDNR-CO	C44 H73 N16 O16	2200.9676	a_{15}	C89 H144 N25 O36 P2
540.3028	DLLTP	C25 H42 N5 O8	1083.4932^{+3}	y_{23}-H_2O^{+3}	C128 H214 N42 O51 P3	2201.9628	QIR(ADP-Ribosyl)DLLTPPTDKPGQ	C88 H143 N26 O36 P2
543.2634	GQDNR-CO	C20 H35 N10 O8	1083.8212^{+3}	y_{23}-NH_3^{+3}	C128 H213 N41 O52 P3	2210.9519	b_{15}-H_2O	C90 H142 N25 O36 P2
547.2752	RS(Phospho)KL-H_2O	C21 H40 N8 O7 P1	1089.4967^{+3}	y_{23}^{+3}	C128 H216 N42 O52 P3	2211.9359	b_{15}-NH_3	C90 H141 N24 O37 P2
548.2341	NRS(Phospho)K-H_2O	C19 H35 N9 O8 P1	1091.4993	KPGQDNRS(Phospho)K	C41 H72 N16 O17 P1	2213.1074	y_{19}-H_2O	C91 H155 N29 O33 P1
548.2592	RS(Phospho)KL-NH_3	C21 H39 N7 O8 P1	1091.5228	PTDKPGQDNR-H_2O	C45 H71 N16 O16	2214.0914	y_{19}-NH_3	C91 H154 N28 O34 P1
548.7666^{+2}	y_8^{+2}	C40 H75 N17 O17 P1	1091.9821^{+2}	a_{15}-H_2O^{+2}	C89 H142 N25 O35 P2	2228.9625	b_{15}	C90 H144 N25 O37 P2
549.2181	NRS(Phospho)K-NH_3	C19 H34 N8 O9 P1	1092.4741^{+2}	a_{15}-NH_3^{+2}	C89 H141 N24 O36 P2	2231.1180	y_{19}	C91 H157 N29 O34 P1
553.1766	DNRS(Phospho)	C17 H30 N8 O11 P1	1092.5069	PTDKPGQDNR-NH_3	C45 H70 N15 O17	2239.9785	a_{16}-H_2O	C91 H145 N26 O36 P2
553.1968^{+2}	a_5-H_2O^{+2}	C40 H63 N14 O19 P2	1095.1801^{+3}	MH-$H_3PO_4^{+3}$	C132 H218 N43 O51 P2	2240.9625	a_{16}-NH_3	C91 H144 N25 O37 P2
553.2477	GQDNR-H_2O	C21 H33 N10 O8	1096.5259	y_8	C40 H75 N17 O17 P1	2257.9890	a_{16}	C91 H147 N26 O37 P2
553.6888^{+2}	a_5-NH_3^{+2}	C40 H62 N13 O20 P2	1100.9874^{+2}	a_{15}^{+2}	C89 H144 N25 O36 P2	2267.9734	b_{16}-H_2O	C92 H145 N26 O37 P2
554.0323^{+4}	y_{19}-H_2O^{+4}	C91 H155 N29 O33 P1	1105.3864	a_5-H_2O	C40 H63 N14 O19 P2	2268.9574	b_{16}-NH_3	C92 H144 N25 O38 P2
554.2318	GQDNR-NH_3	C21 H32 N9 O9	1105.9796^{+2}	b_{15}-H_2O^{+2}	C90 H142 N25 O36 P2	2271.0207	PQIR(ADP-Ribosyl)DLLTPPTDKPGQ-CO	C92 H150 N27 O36 P2
554.2783^{+4}	y_{19}-NH_3^{+4}	C91 H154 N28 O34 P1	1106.3704	a_5-NH_3	C40 H62 N13 O20 P2	2274.9792	IR(ADP-Ribosyl)DLLTPPTDKPGQDN-CO	C90 H146 N27 O38 P2
558.5350^{+4}	y_{19}^{+4}	C91 H157 N29 O34 P1	1106.4716^{+2}	b_{15}-NH_3^{+2}	C90 H141 N24 O37 P2	2281.0050	PQIR(ADP-Ribosyl)DLLTPPTDKPGQ-H_2O	C93 H148 N27 O36 P2
558.6088^{+3}	y_{14}-H_2O^{+3}	C66 H114 N24 O25 P1	1107.0573^{+2}	y_{19}-H_2O^{+2}	C91 H155 N29 O33 P1	2281.9890	PQIR(ADP-Ribosyl)DLLTPPTDKPGQ-NH_3	C93 H147 N26 O37 P2
558.9368^{+3}	y_{14}-NH_3^{+3}	C66 H113 N23 O26 P1	1107.5494^{+2}	y_{19}-NH_3^{+2}	C91 H154 N28 O34 P1	2284.9635	IR(ADP-Ribosyl)DLLTPPTDKPGQDN-H_2O	C91 H144 N27 O38 P2
562.2021^{+2}	a_5^{+2}	C40 H65 N14 O20 P2	1107.6045	DLLTPPTDKPG-CO	C50 H83 N12 O16	2285.9475	IR(ADP-Ribosyl)DLLTPPTDKPGQDN-NH_3	C91 H143 N26 O39 P2
564.3043^{+3}	y_{15}-$H_3PO_4^{+3}$	C71 H120 N25 O23	1109.5334	PTDKPGQDNR	C45 H73 N16 O17	2285.9839	b_{16}	C92 H147 N26 O38 P2
564.6123^{+3}	y_{14}^{+3}	C66 H116 N24 O26 P1	1112.4173	R(ADP-Ribosyl)DLLT-CO	C40 H68 N13 O20 P2	2288.9948	QIR(ADP-Ribosyl)DLLTPPTDKPGQD-CO	C91 H148 N27 O38 P2
565.2858	RS(Phospho)KL	C21 H42 N8 O8 P1	1114.9849^{+2}	b_{15}^{+2}	C90 H144 N25 O37 P2	2298.9792	QIR(ADP-Ribosyl)DLLTPPTDKPGQD-H_2O	C92 H146 N27 O38 P2
566.2446	NRS(Phospho)K	C19 H37 N9 O9 P1	1116.0626^{+2}	y_{19}^{+2}	C91 H157 N29 O34 P1	2299.0156	PQIR(ADP-Ribosyl)DLLTPPTDKPGQ	C93 H150 N27 O37 P2
566.2698	y_4-NH_3	C21 H41 N7 O9 P1	1117.5888	DLLTPPTDKPG-H_2O	C51 H81 N12 O16	2299.9632	QIR(ADP-Ribosyl)DLLTPPTDKPGQD-NH_3	C92 H145 N26 O39 P2
567.1943^{+2}	b_5-H_2O^{+2}	C41 H63 N14 O20 P2	1118.5728	DLLTPPTDKPG-NH_3	C51 H80 N11 O17	2302.9741	IR(ADP-Ribosyl)DLLTPPTDKPGQDN	C91 H146 N27 O39 P2
567.6863^{+2}	b_5-NH_3^{+2}	C41 H62 N13 O21 P2	1120.4929^{+2}	a_{16}-H_2O^{+2}	C91 H145 N26 O36 P2	2316.9897	QIR(ADP-Ribosyl)DLLTPPTDKPGQD	C92 H148 N27 O39 P2
568.2720^{+2}	y_9-H_2O^{+2}	C42 H76 N18 O17 P1	1120.6361	LLTPPTDKPGQ-CO	C51 H86 N13 O15	2317.9962	R(ADP-Ribosyl)DLLTPPTDKPGQDNR-CO	C90 H147 N30 O38 P2
568.3089	PTDKPG-CO	C25 H42 N7 O8	1120.9849^{+2}	a_{16}-NH_3^{+2}	C91 H144 N25 O37 P2	2327.9806	R(ADP-Ribosyl)DLLTPPTDKPGQDNR-H_2O	C91 H145 N30 O38 P2
568.7640^{+2}	y_9-NH_3^{+2}	C42 H75 N17 O18 P1	1121.8355^{+3}	MH-H_2O^{+3}	C132 H219 N43 O54 P3	2328.9646	R(ADP-Ribosyl)DLLTPPTDKPGQDNR-NH_3	C91 H144 N29 O39 P2
571.2583	GQDNR	C21 H35 N10 O9	1122.1635^{+3}	MH-NH_3^{+3}	C132 H218 N42 O55 P3	2345.9911	R(ADP-Ribosyl)DLLTPPTDKPGQDNR	C91 H147 N30 O39 P2
576.1996^{+2}	b_5^{+2}	C41 H65 N14 O21 P2	1122.4017	R(ADP-Ribosyl)DLLT-H_2O	C41 H66 N13 O20 P2	2368.0370	a_{17}-H_2O	C96 H153 N28 O38 P2
576.8153^{+2}	y_{10}-$H_3PO_4^{+2}$	C47 H82 N19 O15	1122.5790	LTPPTDKPGQD-CO	C49 H80 N13 O17	2369.0210	a_{17}-NH_3	C96 H152 N27 O39 P2
577.2773^{+2}	y_9^{+2}	C42 H78 N18 O18 P1	1123.3857	R(ADP-Ribosyl)DLLT-NH_3	C41 H65 N12 O21 P2	2386.0476	a_{17}	C96 H155 N28 O39 P2
578.2933	PTDKPG-H_2O	C26 H40 N7 O8	1123.3969	PQIR(ADP-Ribosyl)D-CO	C40 H65 N14 O20 P2	2386.0476	PQIR(ADP-Ribosyl)DLLTPPTDKPGQD-CO	C96 H155 N28 O39 P2
579.2773	PTDKPG-NH_3	C26 H39 N6 O9	1123.3969	a_5	C40 H65 N14 O20 P2	2396.0319	b_{17}-H_2O	C97 H153 N28 O39 P2
583.2963	y_4	C21 H44 N8 O9 P1	1123.5378	TPPTDKPGQDN-CO	C47 H75 N14 O18	2396.0319	PQIR(ADP-Ribosyl)DLLTPPTDKPGQD-H_2O	C97 H153 N28 O39 P2
590.9597^{+3}	y_{15}-H_2O^{+3}	C71 H121 N25 O26 P1	1124.4537	IR(ADP-Ribosyl)DLL-CO	C42 H72 N13 O19 P2	2397.0160	b_{17}-NH_3	C97 H152 N27 O40 P2
591.2877^{+3}	y_{15}-NH_3^{+3}	C71 H120 N24 O27 P1	1127.8390^{+3}	MH^{+3}	C132 H221 N43 O55 P3	2397.0160	PQIR(ADP-Ribosyl)DLLTPPTDKPGQD-NH_3	C97 H152 N27 O40 P2
595.3814	LLTPPT-CO	C29 H51 N6 O7	1129.4981^{+2}	a_{16}^{+2}	C91 H147 N26 O37 P2	2403.0378	QIR(ADP-Ribosyl)DLLTPPTDKPGQDN-CO	C95 H154 N29 O40 P2

596.3039	**PTDKPG**	C26 H42 N7 O9	1130.6204	**LLTPPTDKPGQ-H_2O**	C52 H84 N13 O15	2413.0221	**QIR(ADP-Ribosyl)DLLTPPTDKPGQDN-H_2O**	C96 H152 N29 O40 P2
596.9633^{+3}	**y_{15}^{+3}**	C71 H123 N25 O27 P1	1131.6045	**LLTPPTDKPGQ-NH_3**	C52 H83 N12 O16	2414.0061	**QIR(ADP-Ribosyl)DLLTPPTDKPGQDN-NH_3**	C96 H151 N28 O41 P2
597.3243	**LTPPTD-CO**	C27 H45 N6 O9	1132.5633	**LLTPPTDKPGQD-H_2O**	C50 H78 N13 O17	2414.0425	**b_{17}**	C97 H155 N28 O40 P2
597.9869^{+3}	**y_{16}-$H_3PO_4^{+3}$**	C75 H127 N26 O25	1133.3813	**b_5-H_2O**	C41 H63 N14 O20 P2	2414.0425	**PQIR(ADP-Ribosyl)DLLTPPTDKPGQD**	C97 H155 N28 O40 P2
599.3148	**TDKPGQ-CO**	C25 H43 N8 O9	1133.3813	**PQIR(ADP-Ribosyl)D-H_2O**	C41 H63 N14 O20 P2	2431.0327	**QIR(ADP-Ribosyl)DLLTPPTDKPGQDN**	C96 H154 N29 O41 P2
605.3657	**LLTPPT-H_2O**	C30 H49 N6 O7	1133.5222	**TPPTDKPGQDN-H_2O**	C48 H73 N14 O18	2431.0803	**IR(ADP-Ribosyl)DLLTPPTDKPGQDNR-CO**	C96 H158 N31 O39 P2
607.3086	**LTPPTD-H_2O**	C28 H43 N6 O9	1133.5473	**LTPPTDKPGQD-NH_3**	C50 H77 N12 O18	2441.0646	**IR(ADP-Ribosyl)DLLTPPTDKPGQDNR-H_2O**	C97 H156 N31 O39 P2
608.3402	**PPTDKP-CO**	C28 H46 N7 O8	1134.3653	**b_5-NH_3**	C41 H62 N13 O21 P2	2442.0487	**IR(ADP-Ribosyl)DLLTPPTDKPGQDNR-NH_3**	C97 H155 N30 O40 P2
609.2991	**TDKPGQ-H_2O**	C26 H41 N8 O9	1134.3653	**PQIR(ADP-Ribosyl)D-NH_3**	C41 H62 N13 O21 P2	2459.0752	**IR(ADP-Ribosyl)DLLTPPTDKPGQDNR**	C97 H158 N31 O40 P2
609.3606	**DLLTPP-CO**	C29 H49 N6 O8	1134.4381	**IR(ADP-Ribosyl)DLL-H_2O**	C43 H70 N13 O19 P2	2483.0640	**a_{18}-H_2O**	C100 H158 N29 O41 P2
610.2831	**TDKPGQ-NH_3**	C26 H40 N7 O10	1134.4903^{+2}	**b_{16}-H_2O^{+2}**	C92 H145 N26 O37 P2	2484.0480	**a_{18}-NH_3**	C100 H157 N28 O42 P2
610.7103^{+2}	**a_6-H_2O^{+2}**	C44 H68 N15 O22 P2	1134.5062	**TPPTDKPGQDN-NH_3**	C48 H72 N13 O19	2484.9946	**R(ADP-Ribosyl)DLLTPPTDKPGQDNRS(Phospho)-CO**	C93 H153 N31 O43 P3
611.2023^{+2}	**a_6-NH_3^{+2}**	C44 H67 N14 O23 P2	1134.9823^{+2}	**b_{16}-NH_3^{+2}**	C92 H144 N25 O38 P2	2494.9789	**R(ADP-Ribosyl)DLLTPPTDKPGQDNRS(Phospho)-H_2O**	C94 H151 N31 O43 P3
612.3100	**KPGQDN-CO**	C25 H42 N9 O9	1135.4221	**IR(ADP-Ribosyl)DLL-NH_3**	C43 H69 N12 O20 P2	2495.9630	**R(ADP-Ribosyl)DLLTPPTDKPGQDNRS(Phospho)-NH_3**	C94 H150 N30 O44 P3
612.3352	**TPPTDK-CO**	C27 H46 N7 O9	1135.5368	**y_9-H_2O**	C42 H76 N18 O17 P1	2500.0905	**PQIR(ADP-Ribosyl)DLLTPPTDKPGQDN-CO**	C100 H161 N30 O41 P2
613.2940	**DKPGQD-CO**	C25 H41 N8 O10	1135.5994	**DLLTPPTDKPG**	C51 H83 N12 O17	2501.0745	**a_{18}**	C100 H160 N29 O42 P2
616.7984^{+2}	**y_{10}-H_2O^{+2}**	C47 H83 N19 O18 P1	1136.5208	**y_9-NH_3**	C42 H75 N17 O18 P1	2510.0749	**PQIR(ADP-Ribosyl)DLLTPPTDKPGQDN-H_2O**	C101 H159 N30 O41 P2
617.2904^{+2}	**y_{10}-NH_3^{+2}**	C47 H82 N18 O19 P1	1139.4282	**QIR(ADP-Ribosyl)DL-CO**	C41 H69 N14 O20 P2	2511.0589	**b_{18}-H_2O**	C101 H158 N29 O42 P2
618.3246	**PPTDKP-H_2O**	C29 H44 N7 O8	1140.4122	**R(ADP-Ribosyl)DLLT**	C41 H68 N13 O21 P2	2511.0589	**PQIR(ADP-Ribosyl)DLLTPPTDKPGQDN-NH_3**	C101 H158 N29 O42 P2
619.3086	**PPTDKP-NH_3**	C29 H43 N6 O9	1143.4956^{+2}	**b_{16}^{+2}**	C92 H147 N26 O38 P2	2512.0429	**b_{18}-NH_3**	C101 H157 N28 O43 P2
619.3450	**DLLTPP-H_2O**	C30 H47 N6 O8	1148.6310	**LLTPPTDKPGQ**	C52 H86 N13 O16	2512.9895	**R(ADP-Ribosyl)DLLTPPTDKPGQDNRS(Phospho)**	C94 H153 N31 O44 P3
619.7156^{+2}	**a_6^{+2}**	C44 H70 N15 O23 P2	1149.4126	**QIR(ADP-Ribosyl)DL-H_2O**	C42 H67 N14 O20 P2	2528.0854	**PQIR(ADP-Ribosyl)DLLTPPTDKPGQDN**	C101 H161 N30 O42 P2
622.2944	**KPGQDN-H_2O**	C26 H40 N9 O9	1150.3966	**QIR(ADP-Ribosyl)DL-NH_3**	C42 H66 N13 O21 P2	2529.0695	**b_{18}**	C101 H160 N29 O43 P2
622.3195	**TPPTDK-H_2O**	C28 H44 N7 O9	1150.5739	**LTPPTDKPGQD**	C50 H80 N13 O18	2559.1389	**QIR(ADP-Ribosyl)DLLTPPTDKPGQDNR-CO**	C101 H166 N33 O41 P2
623.2784	**KPGQDN-NH_3**	C26 H39 N8 O10	1151.3918	**PQIR(ADP-Ribosyl)D**	C41 H65 N14 O21 P2	2569.1232	**QIR(ADP-Ribosyl)DLLTPPTDKPGQDNR-H_2O**	C102 H164 N33 O41 P2
623.2784	**DKPGQD-H_2O**	C26 H39 N8 O10	1151.3918	**b_5**	C41 H65 N14 O21 P2	2570.1072	**QIR(ADP-Ribosyl)DLLTPPTDKPGQDNR-NH_3**	C102 H163 N32 O42 P2
623.3035	**TPPTDK-NH_3**	C28 H43 N6 O10	1151.4841	**TDKPGQDNRS(Phospho)-CO**	C42 H72 N16 O20 P1	2587.1338	**QIR(ADP-Ribosyl)DLLTPPTDKPGQDNR**	C102 H166 N33 O42 P2
623.3763	**LLTPPT**	C30 H51 N6 O8	1151.5327	**TPPTDKPGQDN**	C48 H75 N14 O19	2597.1069	**a_{19}-H_2O**	C104 H164 N31 O43 P2
624.2624	**DKPGQD-NH_3**	C26 H38 N7 O11	1152.4486	**IR(ADP-Ribosyl)DLL**	C43 H72 N13 O20 P2	2598.0787	**IR(ADP-Ribosyl)DLLTPPTDKPGQDNRS(Phospho)-CO**	C99 H164 N32 O44 P3
624.6423^{+3}	**y_{16}-H_2O^{+3}**	C75 H128 N26 O28 P1	1152.6232	**y_{10}-H_3PO_4**	C47 H82 N19 O15	2598.0909	**a_{19}-NH_3**	C104 H163 N30 O44 P2
624.7077^{+2}	**b_6-H_2O^{+2}**	C45 H68 N15 O23 P2	1153.5474	**y_9**	C42 H78 N18 O18 P1	2608.0630	**IR(ADP-Ribosyl)DLLTPPTDKPGQDNRS(Phospho)-H_2O**	C100 H162 N32 O44 P3
624.9703^{+3}	**y_{16}-NH_3^{+3}**	C75 H127 N25 O29 P1	1161.4684	**TDKPGQDNRS(Phospho)-H_2O**	C43 H70 N16 O20 P1	2609.0470	**IR(ADP-Ribosyl)DLLTPPTDKPGQDNRS(Phospho)-NH_3**	C100 H161 N31 O45 P3
625.1998^{+2}	**b_6-NH_3^{+2}**	C45 H67 N14 O24 P2	1162.4525	**TDKPGQDNRS(Phospho)-NH_3**	C43 H69 N15 O21 P1	2613.0896	**R(ADP-Ribosyl)DLLTPPTDKPGQDNRS(Phospho)K-CO**	C99 H165 N33 O44 P3
625.3192	**LTPPTD**	C28 H45 N6 O10	1167.4231	**QIR(ADP-Ribosyl)DL**	C42 H69 N14 O21 P2	2615.1175	**a_{19}**	C104 H166 N31 O44 P2
625.8037^{+2}	**y_{10}^{+2}**	C47 H85 N19 O19 P1	1176.5885	**KPGQDNRS(Phospho)KL-CO**	C46 H83 N17 O17 P1	2623.0739	**R(ADP-Ribosyl)DLLTPPTDKPGQDNRS(Phospho)K-H_2O**	C100 H163 N33 O44 P3
627.3097	**TDKPGQ**	C26 H43 N8 O10	1178.5314	**DKPGQDNRS(Phospho)K-CO**	C44 H77 N17 O19 P1	2624.0579	**R(ADP-Ribosyl)DLLTPPTDKPGQDNRS(Phospho)K-NH_3**	C100 H162 N32 O45 P3
630.6458^{+3}	**y_{16}^{+3}**	C75 H130 N26 O29 P1	1178.5913	**PPTDKPGQDNR-CO**	C49 H80 N17 O17	2625.1018	**b_{19}-H_2O**	C105 H164 N31 O44 P2
633.7130^{+2}	**b_6^{+2}**	C45 H70 N15 O24 P2	1179.4790	**TDKPGQDNRS(Phospho)**	C43 H72 N16 O21 P1	2626.0736	**IR(ADP-Ribosyl)DLLTPPTDKPGQDNRS(Phospho)**	C100 H164 N32 O45 P3
635.6815^{+3}	**y_{17}-$H_3PO_4^{+3}$**	C81 H138 N27 O26	1184.5222^{+2}	**a_{17}-H_2O^{+2}**	C96 H153 N28 O38 P2	2626.0858	**b_{19}-NH_3**	C105 H163 N30 O45 P2
636.3352	**PPTDKP**	C29 H46 N7 O9	1185.0142^{+2}	**a_{17}-NH_3^{+2}**	C96 H152 N27 O39 P2	2641.0845	**R(ADP-Ribosyl)DLLTPPTDKPGQDNRS(Phospho)K**	C100 H165 N33 O45 P3
637.3556	**DLLTPP**	C30 H49 N6 O9	1186.5728	**KPGQDNRS(Phospho)KL-H_2O**	C47 H81 N17 O17 P1	2643.1124	**b_{19}**	C105 H166 N31 O45 P2
640.3049	**KPGQDN**	C26 H42 N9 O10	1187.5569	**KPGQDNRS(Phospho)KL-NH_3**	C47 H80 N16 O18 P1	2656.1916	**PQIR(ADP-Ribosyl)DLLTPPTDKPGQDNR-CO**	C106 H173 N34 O42 P2
640.3161	**PGQDNR-CO**	C25 H42 N11 O9	1188.5157	**DKPGQDNRS(Phospho)K-H_2O**	C45 H75 N17 O19 P1	2666.1760	**PQIR(ADP-Ribosyl)DLLTPPTDKPGQDNR-H_2O**	C107 H171 N34 O42 P2
640.3301	**TPPTDK**	C28 H46 N7 O10	1188.5756	**PPTDKPGQDNR-H_2O**	C50 H78 N17 O17	2667.1600	**PQIR(ADP-Ribosyl)DLLTPPTDKPGQDNR-NH_3**	C107 H170 N33 O43 P2

640.8627^{+2}	y_{11}-$H_3PO_4^{+2}$	C53 H94 N21 O16	1189.4997	DKPGQDNRS(Phospho)K-NH_3	C45 H74 N16 O20 P1	2684.1865	PQIR(ADP-Ribosyl)DLLTPPTDKPGQDNR	C107 H173 N34 O43 P2
641.2889	DKPGQD	C26 H41 N8 O11	1189.5596	PPTDKPGQDNR-NH_3	C50 H77 N16 O18	2726.1372	QIR(ADP-Ribosyl)DLLTPPTDKPGQDNRS(Phospho)-CO	C104 H172 N34 O46 P3
641.4206	y_5-H_3PO_4	C27 H53 N12 O6	1193.5274^{+2}	a_{17}^{+2}	C96 H155 N28 O39 P2	2726.1736	IR(ADP-Ribosyl)DLLTPPTDKPGQDNRS(Phospho)K-CO	C105 H176 N34 O45 P3
650.3005	PGQDNR-H_2O	C26 H40 N11 O9	1198.5196^{+2}	b_{17}-H_2O^{+2}	C97 H153 N28 O39 P2	2726.1736	R(ADP-Ribosyl)DLLTPPTDKPGQDNRS(Phospho)KL-CO	C105 H176 N34 O45 P3
651.2845	PGQDNR-NH_3	C26 H39 N10 O10	1199.0116^{+2}	b_{17}-NH_3^{+2}	C97 H152 N27 O40 P2	2736.1216	QIR(ADP-Ribosyl)DLLTPPTDKPGQDNRS(Phospho)-H_2O	C105 H170 N34 O46 P3
651.3338	NRS(Phospho)KL-CO	C24 H48 N10 O9 P1	1204.5834	KPGQDNRS(Phospho)KL	C47 H83 N17 O18 P1	2736.1580	IR(ADP-Ribosyl)DLLTPPTDKPGQDNRS(Phospho)K-H_2O	C106 H174 N34 O45 P3
653.2403	QDNRS(Phospho)-CO	C21 H38 N10 O12 P1	1206.5263	DKPGQDNRS(Phospho)K	C45 H77 N17 O20 P1	2736.1580	R(ADP-Ribosyl)DLLTPPTDKPGQDNRS(Phospho)KL-H_2O	C106 H174 N34 O45 P3
653.2767	DNRS(Phospho)K-CO	C22 H42 N10 O11 P1	1206.5862	PPTDKPGQDNR	C50 H80 N17 O18	2737.1056	QIR(ADP-Ribosyl)DLLTPPTDKPGQDNRS(Phospho)-NH_3	C105 H169 N33 O47 P3
661.3181	NRS(Phospho)KL-H_2O	C25 H46 N10 O9 P1	1207.5249^{+2}	b_{17}^{+2}	C97 H155 N28 O40 P2	2737.1420	IR(ADP-Ribosyl)DLLTPPTDKPGQDNRS(Phospho)K-NH_3	C106 H173 N33 O46 P3
662.3022	NRS(Phospho)KL-NH_3	C25 H45 N9 O10 P1	1209.4701	R(ADP-Ribosyl)DLLTP-CO	C45 H75 N14 O21 P2	2737.1420	R(ADP-Ribosyl)DLLTPPTDKPGQDNRS(Phospho)KL-NH_3	C106 H173 N33 O46 P3
662.3370^{+3}	y_{17}-H_2O^{+3}	C81 H139 N27 O29 P1	1219.4544	R(ADP-Ribosyl)DLLTP-H_2O	C46 H73 N14 O21 P2	2753.2080	a_{20}-H_2O	C110 H176 N35 O44 P2
662.6650^{+3}	y_{17}-NH_3^{+3}	C81 H138 N26 O30 P1	1220.4133	a_6-H_2O	C44 H68 N15 O22 P2	2754.1321	QIR(ADP-Ribosyl)DLLTPPTDKPGQDNRS(Phospho)	C105 H172 N34 O47 P3
663.2246	QDNRS(Phospho)-H_2O	C22 H36 N10 O12 P1	1220.4385	R(ADP-Ribosyl)DLLTP-NH_3	C46 H72 N13 O22 P2	2754.1685	IR(ADP-Ribosyl)DLLTPPTDKPGQDNRS(Phospho)K	C106 H176 N34 O46 P3
663.2610	DNRS(Phospho)K-H_2O	C23 H40 N10 O11 P1	1221.3973	a_6-NH_3	C44 H67 N14 O23 P2	2754.1685	R(ADP-Ribosyl)DLLTPPTDKPGQDNRS(Phospho)KL	C106 H176 N34 O46 P3
664.2086	QDNRS(Phospho)-NH_3	C22 H35 N9 O13 P1	1225.5014	IR(ADP-Ribosyl)DLLT-CO	C46 H79 N14 O21 P2	2754.1920	a_{20}-NH_3	C110 H175 N34 O45 P2
664.2450	DNRS(Phospho)K-NH_3	C23 H39 N9 O12 P1	1232.5896	y_{10}-H_2O	C47 H83 N19 O18 P1	2771.2186	a_{20}	C110 H178 N35 O45 P2
665.3617	PPTDKPG-CO	C30 H49 N8 O9	1233.5736	y_{10}-NH_3	C47 H82 N18 O19 P1	2781.2029	b_{20}-H_2O	C111 H176 N35 O45 P2
667.2523^{+2}	a_7-H_2O^{+2}	C50 H79 N16 O23 P2	1235.4857	IR(ADP-Ribosyl)DLLT-H_2O	C47 H77 N14 O21 P2	2782.1869	b_{20}-NH_3	C111 H175 N34 O46 P2
667.7443^{+2}	a_7-NH_3^{+2}	C50 H78 N15 O24 P2	1235.6630	LLTPPTDKPGQD-CO	C55 H91 N14 O18	2799.2135	b_{20}	C111 H178 N35 O46 P2
668.3111	PGQDNR	C26 H42 N11 O10	1235.6630	DLLTPPTDKPGQ-CO	C55 H91 N14 O18	2823.1900	PQIR(ADP-Ribosyl)DLLTPPTDKPGQDNRS(Phospho)-CO	C109 H179 N35 O47 P3
668.3405^{+3}	y_{17}^{+3}	C81 H141 N27 O30 P1	1236.4698	IR(ADP-Ribosyl)DLLT-NH_3	C47 H76 N13 O22 P2	2830.3033	y_{20}-H_3PO_4	C112 H187 N38 O44 P2
673.3762^{+3}	y_{18}-$H_3PO_4^{+3}$	C87 H149 N28 O27	1236.4810	PQIR(ADP-Ribosyl)DL-CO	C46 H76 N15 O21 P2	2833.1743	PQIR(ADP-Ribosyl)DLLTPPTDKPGQDNRS(Phospho)-H_2O	C110 H177 N35 O47 P3
675.3461	PPTDKPG-H_2O	C31 H47 N8 O9	1236.6219	LTPPTDKPGQDN-CO	C53 H86 N15 O19	2834.1584	PQIR(ADP-Ribosyl)DLLTPPTDKPGQDNRS(Phospho)-NH_3	C110 H176 N34 O48 P3
676.2576^{+2}	a_7^{+2}	C50 H81 N16 O24 P2	1237.4650	R(ADP-Ribosyl)DLLTP	C46 H75 N14 O22 P2	2839.2577	IR(ADP-Ribosyl)DLLTPPTDKPGQDNRS(Phospho)KL-CO	C111 H187 N35 O46 P3
676.3301	PPTDKPG-NH_3	C31 H46 N7 O10	1238.4239	a_6	C44 H70 N15 O23 P2	2840.2400	a_{21}-H_3PO_4	C113 H181 N36 O46 P2
679.3287	NRS(Phospho)KL	C25 H48 N10 O10 P1	1242.0356^{+2}	a_{18}-H_2O^{+2}	C100 H158 N29 O41 P2	2849.2420	IR(ADP-Ribosyl)DLLTPPTDKPGQDNRS(Phospho)KL-H_2O	C112 H185 N35 O46 P3
680.8459^{+2}	y_{11}-H_2O^{+2}	C53 H95 N21 O19 P1	1242.5276^{+2}	a_{18}-NH_3^{+2}	C100 H157 N28 O42 P2	2850.2260	IR(ADP-Ribosyl)DLLTPPTDKPGQDNRS(Phospho)KL-NH_3	C112 H184 N34 O47 P3
681.2352	QDNRS(Phospho)	C22 H38 N10 O13 P1	1245.6474	LLTPPTDKPGQD-H_2O	C56 H89 N14 O18	2851.1849	PQIR(ADP-Ribosyl)DLLTPPTDKPGQDNRS(Phospho)	C110 H179 N35 O48 P3
681.2498^{+2}	b_7-H_2O^{+2}	C51 H79 N16 O24 P2	1245.6474	DLLTPPTDKPGQ-H_2O	C56 H89 N14 O18	2854.2322	QIR(ADP-Ribosyl)DLLTPPTDKPGQDNRS(Phospho)K-CO	C110 H184 N36 O47 P3
681.2716	DNRS(Phospho)K	C23 H42 N10 O12 P1	1246.4653	PQIR(ADP-Ribosyl)DL-H_2O	C47 H74 N15 O21 P2	2864.2165	QIR(ADP-Ribosyl)DLLTPPTDKPGQDNRS(Phospho)K-H_2O	C111 H182 N36 O47 P3
681.3379^{+2}	y_{11}-NH_3^{+2}	C53 H94 N20 O20 P1	1246.6062	LTPPTDKPGQDN-H_2O	C54 H84 N15 O19	2865.2006	QIR(ADP-Ribosyl)DLLTPPTDKPGQDNRS(Phospho)K-NH_3	C111 H181 N35 O48 P3
681.7418^{+2}	b_7-NH_3^{+2}	C51 H78 N15 O25 P2	1246.6314	LLTPPTDKPGQD-NH_3	C56 H88 N13 O19	2867.2526	IR(ADP-Ribosyl)DLLTPPTDKPGQDNRS(Phospho)KL	C112 H187 N35 O47 P3
689.0575^{+4}	a_{20}-H_2O^{+4}	C110 H176 N35 O44 P2	1246.6314	DLLTPPTDKPGQ-NH_3	C56 H88 N13 O19	2868.2350	b_{21}-H_3PO_4	C114 H181 N36 O47 P2
689.3035^{+4}	a_{20}-NH_3^{+4}	C110 H175 N34 O45 P2	1247.4494	PQIR(ADP-Ribosyl)DL-NH_3	C47 H73 N14 O22 P2	2882.2271	QIR(ADP-Ribosyl)DLLTPPTDKPGQDNRS(Phospho)K	C111 H184 N36 O48 P3
689.8512^{+2}	y_{11}^{+2}	C53 H97 N21 O20 P1	1247.5903	LTPPTDKPGQDN-NH_3	C54 H83 N14 O20	2910.2696	y_{20}-H_2O	C112 H188 N38 O47 P3
690.2551^{+2}	b_7^{+2}	C51 H81 N16 O25 P2	1248.4082	b_6-H_2O	C45 H68 N15 O23 P2	2911.2537	y_{20}-NH_3	C112 H187 N37 O48 P3
693.3566	PPTDKPG	C31 H49 N8 O10	1248.5369	PTDKPGQDNRS(Phospho)-CO	C47 H79 N17 O21 P1	2920.2064	a_{21}-H_2O	C113 H182 N36 O49 P3
693.5601^{+4}	a_{20}^{+4}	C110 H178 N35 O45 P2	1249.3922	b_6-NH_3	C45 H67 N14 O24 P2	2921.1904	a_{21}-NH_3	C113 H181 N35 O50 P3
695.9729^{+3}	a_{14}-H_2O^{+3}	C84 H135 N24 O34 P2	1250.6001	y_{10}	C47 H85 N19 O19 P1	2928.2802	y_{20}	C112 H190 N38 O48 P3
696.0562^{+4}	b_{20}-H_2O^{+4}	C111 H176 N35 O45 P2	1251.0409^{+2}	a_{18}^{+2}	C100 H160 N29 O42 P2	2938.2169	a_{21}	C113 H184 N36 O50 P3
696.3009^{+3}	a_{14}-NH_3^{+3}	C84 H134 N23 O35 P2	1252.5123	QIR(ADP-Ribosyl)DLL-CO	C47 H80 N15 O21 P2	2943.3874	y_{21}-H_3PO_4	C118 H198 N39 O45 P2
696.3022^{+4}	b_{20}-NH_3^{+4}	C111 H175 N34 O46 P2	1253.4963	IR(ADP-Ribosyl)DLLT	C47 H79 N14 O22 P2	2948.2013	b_{21}-H_2O	C114 H182 N36 O50 P3
696.3675	PTDKPGQ-CO	C30 H50 N9 O10	1256.0331^{+2}	b_{18}-H_2O^{+2}	C101 H158 N29 O42 P2	2949.1853	b_{21}-NH_3	C114 H181 N35 O51 P3

698.3762^{+2}	y_{12}-$H_3PO_4^{+2}$	C57 H99 N22 O19	1256.5251^{+2}	b_{18}-NH_3^{+2}	C101 H157 N28 O43 P2	2951.2850	PQIR(ADP-Ribosyl)DLLTPPTDKPGQDNRS(Phospho)K-CO	C115 H191 N37 O48 P3
700.0317^{+3}	y_{18}-H_2O^{+3}	C87 H150 N28 O30 P1	1258.5212	PTDKPGQDNRS(Phospho)-H_2O	C48 H77 N17 O21 P1	2961.2693	PQIR(ADP-Ribosyl)DLLTPPTDKPGQDNRS(Phospho)K-H_2O	C116 H189 N37 O48 P3
700.3597^{+3}	y_{18}-NH_3^{+3}	C87 H149 N27 O31 P1	1259.5052	PTDKPGQDNRS(Phospho)-NH_3	C48 H76 N16 O22 P1	2962.2533	PQIR(ADP-Ribosyl)DLLTPPTDKPGQDNRS(Phospho)K-NH_3	C116 H188 N36 O49 P3
700.5588^{+4}	b_{20}^{+4}	C111 H178 N35 O46 P2	1262.4966	QIR(ADP-Ribosyl)DLL-H_2O	C48 H78 N15 O21 P2	2966.2119	b_{21}	C114 H184 N36 O51 P3
701.9764^{+3}	a_{14}^{+3}	C84 H137 N24 O35 P2	1263.4807	QIR(ADP-Ribosyl)DLL-NH_3	C48 H77 N14 O22 P2	2967.3163	QIR(ADP-Ribosyl)DLLTPPTDKPGQDNRS(Phospho)KL-CO	C116 H195 N37 O48 P3
705.3046^{+3}	b_{14}-H_2O^{+3}	C85 H135 N24 O35 P2	1263.6579	LLTPPTDKPGQD	C56 H91 N14 O19	2968.3350	a_{22}-H_3PO_4	C119 H193 N38 O47 P2
705.6326^{+3}	b_{14}-NH_3^{+3}	C85 H134 N23 O36 P2	1263.6579	DLLTPPTDKPGQ	C56 H91 N14 O19	2977.3006	QIR(ADP-Ribosyl)DLLTPPTDKPGQDNRS(Phospho)KL-H_2O	C117 H193 N37 O48 P3
706.0352^{+3}	y_{18}^{+3}	C87 H152 N28 O31 P1	1264.4759	PQIR(ADP-Ribosyl)DL	C47 H76 N15 O22 P2	2978.2846	QIR(ADP-Ribosyl)DLLTPPTDKPGQDNRS(Phospho)KL-NH_3	C117 H192 N36 O49 P3
706.3519	PTDKPGQ-H_2O	C31 H48 N9 O10	1264.6168	LTPPTDKPGQDN	C54 H86 N15 O20	2979.2799	PQIR(ADP-Ribosyl)DLLTPPTDKPGQDNRS(Phospho)K	C116 H191 N37 O49 P3
707.3359	PTDKPGQ-NH_3	C31 H47 N8 O11	1265.0384^{+2}	b_{18}^{+2}	C101 H160 N29 O43 P2	2995.3112	QIR(ADP-Ribosyl)DLLTPPTDKPGQDNRS(Phospho)KL	C117 H195 N37 O49 P3
708.3313^{+4}	y_{20}-$H_3PO_4^{+4}$	C112 H187 N38 O44 P2	1266.4188	b_6	C45 H70 N15 O24 P2	2996.3299	b_{22}-H_3PO_4	C120 H193 N38 O48 P2
709.3879	TPPTDKP-CO	C32 H53 N8 O10	1276.5318	PTDKPGQDNRS(Phospho)	C48 H79 N17 O22 P1	3023.3537	y_{21}-H_2O	C118 H199 N39 O48 P3
710.2617	GQDNRS(Phospho)-CO	C23 H41 N11 O13 P1	1279.5791	TDKPGQDNRS(Phospho)K-CO	C48 H84 N18 O21 P1	3024.3377	y_{21}-NH_3	C118 H198 N38 O49 P3
710.4083	DLLTPPT-CO	C33 H56 N7 O10	1279.6389	TPPTDKPGQDNR-CO	C53 H87 N18 O19	3041.3643	y_{21}	C118 H201 N39 O49 P3
710.4083	LLTPPTD-CO	C33 H56 N7 O10	1280.5072	QIR(ADP-Ribosyl)DLL	C48 H80 N15 O22 P2	3048.3013	a_{22}-H_2O	C119 H194 N38 O50 P3
710.8155^{+4}	a_{21}-$H_3PO_4^{+4}$	C113 H181 N36 O46 P2	1280.7182	y_{11}-H_3PO_4	C53 H94 N21 O16	3049.2854	a_{22}-NH_3	C119 H193 N37 O51 P3
711.3081^{+3}	b_{14}^{+3}	C85 H137 N24 O36 P2	1289.5634	TDKPGQDNRS(Phospho)K-H_2O	C49 H82 N18 O21 P1	3064.3690	PQIR(ADP-Ribosyl)DLLTPPTDKPGQDNRS(Phospho)KL-CO	C121 H202 N38 O49 P3
711.7185^{+3}	y_{19}-$H_3PO_4^{+3}$	C91 H154 N29 O30	1289.6233	TPPTDKPGQDNR-H_2O	C54 H85 N18 O19	3066.3119	a_{22}	C119 H196 N38 O51 P3
714.3417	TDKPGQD-CO	C29 H48 N9 O12	1290.5474	TDKPGQDNRS(Phospho)K-NH_3	C49 H81 N17 O22 P1	3071.4460	y_{22}-H_3PO_4	C123 H206 N41 O47 P2
717.8142^{+4}	b_{21}-$H_3PO_4^{+4}$	C114 H181 N36 O47 P2	1290.6073	TPPTDKPGQDNR-NH_3	C54 H84 N17 O20	3074.3534	PQIR(ADP-Ribosyl)DLLTPPTDKPGQDNRS(Phospho)KL-H_2O	C122 H200 N38 O49 P3
719.3723	TPPTDKP-H_2O	C33 H51 N8 O10	1291.6154	DKPGQDNRS(Phospho)KL-CO	C50 H88 N18 O20 P1	3075.3374	PQIR(ADP-Ribosyl)DLLTPPTDKPGQDNRS(Phospho)KL-NH_3	C122 H199 N37 O50 P3
720.2461	GQDNRS(Phospho)-H_2O	C24 H39 N11 O13 P1	1299.0571^{+2}	a_{19}-H_2O^{+2}	C104 H164 N31 O43 P2	3076.2963	b_{22}-H_2O	C120 H194 N38 O51 P3
720.3563	TPPTDKP-NH_3	C33 H50 N7 O11	1299.5491^{+2}	a_{19}-NH_3^{+2}	C104 H163 N30 O44 P2	3077.2803	b_{22}-NH_3	C120 H193 N37 O52 P3
720.3927	DLLTPPT-H_2O	C34 H54 N7 O10	1301.5998	DKPGQDNRS(Phospho)KL-H_2O	C51 H86 N18 O20 P1	3081.4191	a_{23}-H_3PO_4	C125 H204 N39 O48 P2
720.3927	LLTPPTD-H_2O	C34 H54 N7 O10	1302.5838	DKPGQDNRS(Phospho)KL-NH_3	C51 H85 N17 O21 P1	3092.3639	PQIR(ADP-Ribosyl)DLLTPPTDKPGQDNRS(Phospho)KL	C122 H202 N38 O50 P3
721.2301	GQDNRS(Phospho)-NH_3	C24 H38 N10 O14 P1	1306.5229	R(ADP-Ribosyl)DLLTPP-CO	C50 H82 N15 O22 P2	3094.3068	b_{22}	C120 H196 N38 O52 P3
722.3709	y_5-NH_3	C27 H53 N11 O10 P1	1307.5740	TDKPGQDNRS(Phospho)K	C49 H84 N18 O22 P1	3109.4140	b_{23}-H_3PO_4	C126 H204 N39 O49 P2
723.7944^{+2}	a_8-H_2O^{+2}	C56 H90 N17 O24 P2	1307.6339	TPPTDKPGQDNR	C54 H87 N18 O20	3112.3174	b_{22}+H_2O	C120 H198 N38 O53 P3
724.2864^{+2}	a_8-NH_3^{+2}	C56 H89 N16 O25 P2	1308.0624^{+2}	a_{19}^{+2}	C104 H166 N31 O44 P2	3151.4123	y_{22}-H_2O	C123 H207 N41 O50 P3
724.3260	TDKPGQD-H_2O	C30 H46 N9 O12	1313.0545^{+2}	b_{19}-H_2O^{+2}	C105 H164 N31 O44 P2	3152.3963	y_{22}-NH_3	C123 H206 N40 O51 P3
724.3624	PTDKPGQ	C31 H50 N9 O11	1313.5466^{+2}	b_{19}-NH_3^{+2}	C105 H163 N30 O45 P2	3161.3854	a_{23}-H_2O	C125 H205 N39 O51 P3
725.3101	TDKPGQD-NH_3	C30 H45 N8 O13	1316.5072	R(ADP-Ribosyl)DLLTPP-H_2O	C51 H80 N15 O22 P2	3162.3694	a_{23}-NH_3	C125 H204 N38 O52 P3
725.4192	LTPPTDK-CO	C33 H57 N8 O10	1317.4912	R(ADP-Ribosyl)DLLTPP-NH_3	C51 H79 N14 O23 P2	3168.4987	y_{23}-H_3PO_4	C128 H213 N42 O48 P2
727.3369	DKPGQDN-CO	C29 H47 N10 O12	1319.6104	DKPGQDNRS(Phospho)KL	C51 H88 N18 O21 P1	3169.4228	y_{22}	C123 H209 N41 O51 P3
728.3229^{+4}	y_{20}-H_2O^{+4}	C112 H188 N38 O47 P3	1322.0598^{+2}	b_{19}^{+2}	C105 H166 N31 O45 P2	3179.3960	a_{23}	C125 H207 N39 O52 P3
728.3238^{+3}	a_{15}-H_2O^{+3}	C89 H142 N25 O35 P2	1322.5542	IR(ADP-Ribosyl)DLLTP-CO	C51 H86 N15 O22 P2	3189.3803	b_{23}-H_2O	C126 H205 N39 O52 P3
728.5689^{+4}	y_{20}-NH_3^{+4}	C112 H187 N37 O48 P3	1332.5385	IR(ADP-Ribosyl)DLLTP-H_2O	C52 H84 N15 O22 P2	3190.3643	b_{23}-NH_3	C126 H204 N38 O53 P3
728.6519^{+3}	a_{15}-NH_3^{+3}	C89 H141 N24 O36 P2	1333.4974	a_7-H_2O	C50 H79 N16 O23 P2	3207.3909	b_{23}	C126 H207 N39 O53 P3
730.8071^{+4}	a_{21}-H_2O^{+4}	C113 H182 N36 O49 P3	1333.5225	IR(ADP-Ribosyl)DLLTP-NH_3	C52 H83 N14 O23 P2	3225.4014	b_{23}+H_2O	C126 H209 N39 O54 P3
731.0531^{+4}	a_{21}-NH_3^{+4}	C113 H181 N35 O50 P3	1334.4814	a_7-NH_3	C50 H78 N15 O24 P2	3248.4650	y_{23}-H_2O	C128 H214 N42 O51 P3
732.7996^{+2}	a_8^{+2}	C56 H92 N17 O25 P2	1334.5178	R(ADP-Ribosyl)DLLTPP	C51 H82 N15 O23 P2	3249.4491	y_{23}-NH_3	C128 H213 N41 O52 P3
732.8255^{+4}	y_{20}^{+4}	C112 H190 N38 O48 P3	1345.5896	PPTDKPGQDNRS(Phospho)-CO	C52 H86 N18 O22 P1	3266.4756	y_{23}	C128 H216 N42 O52 P3
734.3274^{+3}	a_{15}^{+3}	C89 H144 N25 O36 P2	1349.5651	PQIR(ADP-Ribosyl)DLL-CO	C52 H87 N16 O22 P2	3283.5257	MH-H_3PO_4	C132 H218 N43 O51 P2

735.3097^{+4}	**a_{21}^{+4}**	C113 H184 N36 O50 P3	1349.7060	**LLTPPTDKPGQDN-CO**	C59 H97 N16 O20	3363.4920	**MH-H_2O**	C132 H219 N43 O54 P3
735.4036	**LTPPTDK-H_2O**	C34 H55 N8 O10	1350.5491	**IR(ADP-Ribosyl)DLLTP**	C52 H86 N15 O23 P2	3364.4760	**MH-NH_3**	C132 H218 N42 O55 P3
736.3876	**LTPPTDK-NH_3**	C34 H54 N7 O11	1350.6900	**DLLTPPTDKPGQD-CO**	C59 H96 N15 O21	3381.5026	**MH**	C132 H221 N43 O55 P3
